# A Cluster Randomised Controlled Trial of a Pharmacist-Led Collaborative Intervention to Improve Statin Prescribing and Attainment of Cholesterol Targets in Primary Care

**DOI:** 10.1371/journal.pone.0113370

**Published:** 2014-11-18

**Authors:** Richard Lowrie, Suzanne M. Lloyd, Alex McConnachie, Jill Morrison

**Affiliations:** 1 Pharmacy and Prescribing Support Unit, NHS Greater Glasgow and Clyde, Glasgow, Scotland, United Kingdom; 2 Robertson Centre for Biostatistics, Boyd Orr Building, University of Glasgow, Glasgow, Scotland, United Kingdom; 3 General Practice and Primary Care, Institute of Health and Wellbeing, University of Glasgow, Glasgow, Scotland, United Kingdom; University of Milan, Italy

## Abstract

**Background:**

Small trials with short term follow up suggest pharmacists’ interventions targeted at healthcare professionals can improve prescribing. In comparison with clinical guidance, contemporary statin prescribing is sub-optimal and achievement of cholesterol targets falls short of accepted standards, for patients with atherosclerotic vascular disease who are at highest absolute risk and who stand to obtain greatest benefit. We hypothesised that a pharmacist-led complex intervention delivered to doctors and nurses in primary care, would improve statin prescribing and achievement of cholesterol targets for incident and prevalent patients with vascular disease, beyond one year.

**Methods:**

We allocated general practices to a 12-month Statin Outreach Support (SOS) intervention or usual care. SOS was delivered by one of 11 pharmacists who had received additional training. SOS comprised academic detailing and practical support to identify patients with vascular disease who were not prescribed a statin at optimal dose or did not have cholesterol at target, followed by individualised recommendations for changes to management. The primary outcome was the proportion of patients achieving cholesterol targets. Secondary outcomes were: the proportion of patients prescribed simvastatin 40 mg with target cholesterol achieved; cholesterol levels; prescribing of simvastatin 40 mg; prescribing of any statin and the proportion of patients with cholesterol tested. Outcomes were assessed after an average of 1.7 years (range 1.4–2.2 years), and practice level simvastatin 40 mg prescribing was assessed after 10 years.

**Findings:**

We randomised 31 practices (72 General Practitioners (GPs), 40 nurses). Prior to randomisation a subset of eligible patients were identified to characterise practices; 40% had cholesterol levels below the target threshold. Improvements in data collection procedures allowed identification of all eligible patients (n = 7586) at follow up. Patients in practices allocated to SOS were significantly more likely to have cholesterol at target (69.5% vs 63.5%; OR 1.11, CI 1.00–1.23; p = 0.043) as a result of improved simvastatin prescribing. Subgroup analysis showed the primary outcome was achieved by prevalent but not incident patients. Statistically significant improvements occurred in all secondary outcomes for prevalent patients and all but one secondary outcome (the proportion of patients with cholesterol tested) for incident patients. SOS practices prescribed more simvastatin 40 mg than usual care practices, up to 10 years later.

**Interpretation:**

Through a combination of educational and organisational support, a general practice based pharmacist led collaborative intervention can improve statin prescribing and achievement of cholesterol targets in a high-risk primary care based population.

**Trial Registration:**

International Standard Randomised Controlled Trials Register ISRCTN61233866

## Introduction

Pharmacists from across the world aim to improve prescribing and patient outcomes directly by consulting with patients through pharmacist-led medication review [1], [2], or indirectly by delivering educational prescribing support to healthcare professionals [3]–[6]. Collaborative care models involving pharmacists targeting primary care physicians have existed for over 15 years [7], [8], and systematic reviews of randomised studies suggest a reduction in cardiovascular risk through pharmacist intervention at patient [9] and healthcare professional level [10]. However, trials involve few participants or pharmacists, have shortcomings in design, inclusion criteria limit generalisability, outcomes are confined to prescribing change and follow up is limited to one year at most [11], [12]. Increasing demands from an aging population with more long term conditions and an ongoing need for quality improvement in prescribing has intensified the need for better evidence of long term effects of pharmacists’ expanded professional (clinical) roles [1], [4], [13], [14].

Landmark studies involving patients with established atherosclerotic disease show that statin prescribing, with or without achievement of target cholesterol, reduces morbidity and mortality [15], [16]. The largest trial to date (the Heart Protection Study), tested the effect of Simvastatin 40 mg on all cause mortality and found a statistically significant reduction from 14.7% to 12.9% in patients with the following conditions: Previous Myocardial Infarction;

Pre- or post-Coronary Artery Bypass Graft; Pre- or post-Angioplasty; Angina; Ischaemic Heart Disease; Angiographic coronary artery disease; Ischaemic stroke or Transient Ischaemic Attack; Peripheral Arterial Disease; diabetes aged ≥ 40 years and those with treated hypertension aged at least 65 years [15]. Despite the availability of guidance [17], [18] and an understanding of how to integrate evidence into practice [19], clinical practice lags behind clinical trial evidence and more implementation research is needed [20]. Identification of eligible patients, prescribing, dosing and achievement of cholesterol goals all remain suboptimal or, at best, highly variable, across healthcare systems [19]–[25]. Together, this suggests there may be merit in an intervention combining educational and organisational support, targeted at prescribers and the processes used in primary care general practices to offer statins and achieve cholesterol targets in patients at highest risk of vascular events.

In the UK, patients with atherosclerotic vascular disease are regarded as a priority for treatment with statins regardless of baseline cholesterol levels [17], [26]. In practice, audit standards recommend target cholesterol levels of less than 5 mmol/l (or less than 4.2 mmol/l for patients with a Coronary Artery Bypass Graft) [26]–[28].

In a large-scale, cluster randomised controlled trial within the National Health Service (NHS) in Scotland, we tested the hypothesis that a multifaceted Statin Outreach Support (SOS) intervention targeted at healthcare professionals, by general practice-attached pharmacists promoting the uptake of Simvastatin 40 mg and the prescribing of other statins for patients with atherosclerotic vascular disease, improves attainment of cholesterol targets and statin prescribing.

## Methods

Trial design is published [29] and consistent with Consolidated Standards of Reporting Trials [30]. The protocol for this trial and supporting CONSORT checklist are available as supporting information; see Checklist S1 and Protocol S1. The study was funded and sponsored by NHS Greater Glasgow and Clyde. The study sponsor had no role in the study design, delivery, analyses or preparation of the manuscript.

The study is registered, number ISRCTN61233866.

### Ethics statement

The study was approved by Greater Glasgow Community/Primary Care Local Research Ethics Committee. In accordance with Ethical committee approval, informed, written consent was required from each participating practice; individual patient consent was not required.

### Study design

#### Practices

All primary care practices (n = 238, population 962,106) in Greater Glasgow Health Board (GGHB) were eligible to participate. Sixty nine were single handed (SH; with only one General Practitioner (GP) and 169 were group (G; with more than one GP) practices. On average, practices served approximately 4,250 patients and had three salaried GPs, two salaried nurses and attached staff e.g. district nurses [31].

Forty nine practices (25 SH and 24 G) were randomly selected and invited to participate. After a face-to-face meeting with the principal investigator to explain study procedures, 31 (15 SH, 16 G) practices provided written informed consent [29].

#### Patients

Patient level inclusion criteria were similar but not identical to the largest statin study to date (the Heart Protection Study), published before the SOS trial commenced [15]. We identified patients who had confirmed atherosclerotic vascular disease (secondary prevention), on the basis of at least one of the following diagnoses appearing in the form of a Read Code (the hierarchical clinical coding system used in the UK) in the primary care medical records which contain the complete set of patients’ health and prescribing information:

Previous Myocardial Infarction;

Pre- or post-Coronary Artery Bypass Graft;

Pre- or post-Angioplasty;

Angina;

Ischaemic Heart Disease;

Angiographic coronary artery disease;

Ischaemic stroke or Transient Ischaemic Attack;

Peripheral Arterial Disease;

Patients with diabetes aged ≥ 40 years.

Patients with these diagnoses were included because they formed part of the inclusion criteria for the Heart Protection Study (which showed the benefits of simvastatin 40 mg) [15] and they are regarded as a priority group in clinical guidelines and practice [17], [26], [28]. We chose not to include patients with treated hypertension (if also male and aged at least 65 years) because patients with this entry criteria alone constituted only 1% of patients in HPS and Scottish National Health Service policy assumes they would be identified and managed through routine primary prevention activity and guidance.

We conducted two cross sectional surveys of patient level data: one at baseline to characterise the practice populations, and the other at follow up for collection of study outcomes. This approach precluded individual patient follow up although it was possible, at follow up, to identify which patients had been eligible at baseline (prevalent) or newly diagnosed or registered with the practice since randomisation (incident).

#### Baseline data collection

Baseline data were collected before randomisation in each participating practice as described previously [29], and as follows. Using computerised Read code searches in each practice, we produced lists of eligible patients, to minimise selection and observation bias. In 26 practices, each eligible patient’s electronic and paper-based record was consulted and in the remaining five, largest practices, because of time constraints, every third patient’s record was accessed. Baseline data was therefore collected on a subset of eligible patients rather than all eligible patients. Patient level baseline data (File S1, Page 27) were collected in a cross-sectional sample of 4,040 patients’ records in all 31 participating general practices (clusters) at baseline.

We categorised recruited General Practices depending on whether they were a Group practice (n = 16) with several GPs, or a Single Handed practice (n = 15) with only one GP in the practice. We then separated practices into two groups according to whether they were G or SH.

#### Ordering

Within G or SH strata we ordered practices using the following ratio, which was calculated from a summary of each practice’s baseline data:

Number of patients with cholesterol in the target range/Number of patients with vascular disease.

Within each stratum, we arranged practices in ascending order of ratio (from lowest to highest) and numbered each practice sequentially.

#### Pairing (matching)

Ordered and numbered within SH and G strata, we paired practices so that those with similar ratios were matched. This generated eight pairs of G practices and six pairs of SH practices with one triplet of SH practices (31 practices in total).

#### Randomisation

We then randomly allocated (using a table of random numbers) one practice from each matched pair into the SOS arm and the other practice into the usual care arm. In the triplet, two practices were randomly allocated to the SOS arm and one to usual care.

This type of matched cluster design (‘matched pair’ in which one of two matched clusters in a stratum are randomly assigned to each intervention) is frequently adopted in cluster randomised trials [32]. The allocation was, therefore, based on clusters rather than on individuals and the identity of all the practices was concealed until after allocation to SOS intervention (described below) or usual care.

#### SOS intervention

The intervention was delivered from January through December 2004.

#### Pharmacists delivering the intervention

Eleven NHS employee pharmacists received 41 contact hours of training (File S1, page 5) focusing on academic detailing; therapeutics and general practice call/recall procedures. Training was delivered by Cardiologists, Practice Nurses, GPs, and the research team. None of the pharmacists had previous experience delivering the intervention. Seven pharmacists delivered the SOS intervention to one practice each and four pharmacists were allocated two practices each.

#### Content and delivery of the intervention

Pharmacists worked one day per week in their allocated practice(s) for one year [29]. Using patient-level and summary prescribing information, pharmacists provided organisational support comprising identification of patients with potential for statin initiation and optimization, by screening medical records. Through discussion with GPs and Nurses, they identified individuals’ barriers to prescribing change e.g. scepticism about the strength of evidence for initiating a statin. Pharmacists subsequently devised ways to help GPs and Nurses overcome these barriers then provided individualized support to enact plans. Pharmacists aimed to improve practices’ patient call and recall by systematically identifying and categorising all eligible patients in the disease register according to what the practice needed to do for those patients who were not prescribed simvastatin 40 mg (or another statin at sufficient dose, if that was the individual GP’s strong preference (File S1, page 4, Table 1) or if recommended by local guidance (File S1, Page 7). Using information from patients’ records and comparing with management as described in local guidance, pharmacists prepared individualised, written, evidence based recommendations for each patient and passed these to the GP for consideration and implementation. Practices subsequently contacted patients to advise of changes to their prescription and/or invite for a cholesterol test. Pharmacists systematically followed up each patient’s plan to maximise patient uptake and minimise dropout.

**Table 1:** Baseline patient characteristics.

	SOS (15 practices; n = 2373 patients)	Usual Care (15 practices; n = 1667 patients)	P-value
Age (years; mean (SD)	68.2 (12.1)	68.5 (12.0)	0.311
Sex, male	1207/2373 (52.9%)	890/1667 (53.4%)	0.192
**Qualifying diagnosis (No (%) of patients with each disease)**			
Angina/Ischaemic Heart Disease	1170/2373 (49.3%)	674/1667 (40.4%)	<0.001
Diabetes Mellitus, age ≥45 years	825/2373 (34.8%)	647/1667 (38.8%)	0.342
Myocardial Infarction	495/2373 (20.8%)	355/1667 (21.2%)	0.332
Cerebrovascular event	334/2373 (14.1%)	236/1667 (14.1%)	0.112
Peripheral Vascular Disease	286/2373 (12.0%)	161/1667 (9.6%)	0.202
Transient Ischaemic Attack	223/2373 (9.4%)	121/1667 (7.2%)	0.162
Coronary Artery Bypass Graft	200/2373 (8.4%)	144/1667 (8.6%)	0.542
Angioplasty	104/2373 (4.4%)	67/1667 (4.0%)	0.672
Vascular co-morbidities (mean (SD)	1.53 (0.8)	1.44 (0.7)	<0.001
Vascular co-morbidities excepting angina	0.81 (0.7)	0.84 (0.7)	0.761
**Statin prescribing and cholesterol**			
Cholesterol target achieved	878/1768 (49.7%)	680/1307 (52.0%)	0.482
Simvastatin 40 mg and target cholesterol achieved	91/2373	72/1667	0.432
Cholesterol level (mean, SD)	5.08 mmol/l (1.1 mmol/l)	5.01 mmol/l (1.1 mmol/l)	0.141
Prescribed Simvastatin 40 mg	211/2373 (8.9%)	157/1667 (9.4%)	0.892
Prescribed Simvastatin any dose	529/2373 (22.3%)	443/1667 (26.6%)	0.152
Prescribed any statin	917/2373 (38.6%)	738/1667 (44.3%)	<0.001
Cholesterol tested	1768/2373 (74.5%)	1307/1667 (78.4%)	0.012
Cholesterol level, all patients with a statin	4.79 mmol/l (1.2 mmol/l)	4.71 mmol/l (1.1 mmol/l)	0.201
Cholesterol level, all patients without a statin	5.11 mmol/l (1.1 mmol/l)	5.08 mmol/l (1.0 mmol/l)	0.821
Statin prescribed at optimal dose	520/2373 (21.9%)	408/1667 (24.5%)	<0.001

Pharmacists also provided educational support. This comprised three face-to-face, one-to-one meetings between pharmacist and GP, and pharmacist and nurse, at four-month intervals. Pharmacists provided individualised, unbiased information (in response to learning needs identified during the first face to face meeting) about statins, statin trials, feedback on the practice’s progress with offering a statin to eligible patients, and the cost effectiveness of improving statin prescribing (File S1, page 3). Pharmacists recommended the prescription of Simvastatin 40 mg (or other statins, if indicated) for patients who were not receiving one, and dose-intensification for patients who were prescribed a statin at a sub-optimal dose. Practices were free to choose to prescribe a statin other than simvastatin e.g. atorvastatin, pravastatin or fluvastatin, but these choices were countered in the public interest, by the pharmacist describing the lower cost of simvastatin and greater weight of evidence. Evidence based statin prescribing and dosing was encouraged through repetition and reinforcement of key learning points.

These recommendations were given regardless of patients’ baseline cholesterol levels consistent with local guidance (File S1, page 7), National guidance [17], [18] and best available evidence [15]. Pharmacists recommended no changes when patients were prescribed Simvastatin at a dose of at least 40 mg and target cholesterol was achieved. Local opinion leaders’ views were sought for questions arising from discussions that could not be routinely addressed (File S1, page 3) [69].

After working in the practice one day per week for a year, delivering the intervention, Pharmacists left their respective practices and were not replaced, until after follow-up data were collected from the last practice in March 2007.

#### Usual care

**Figure 1:**
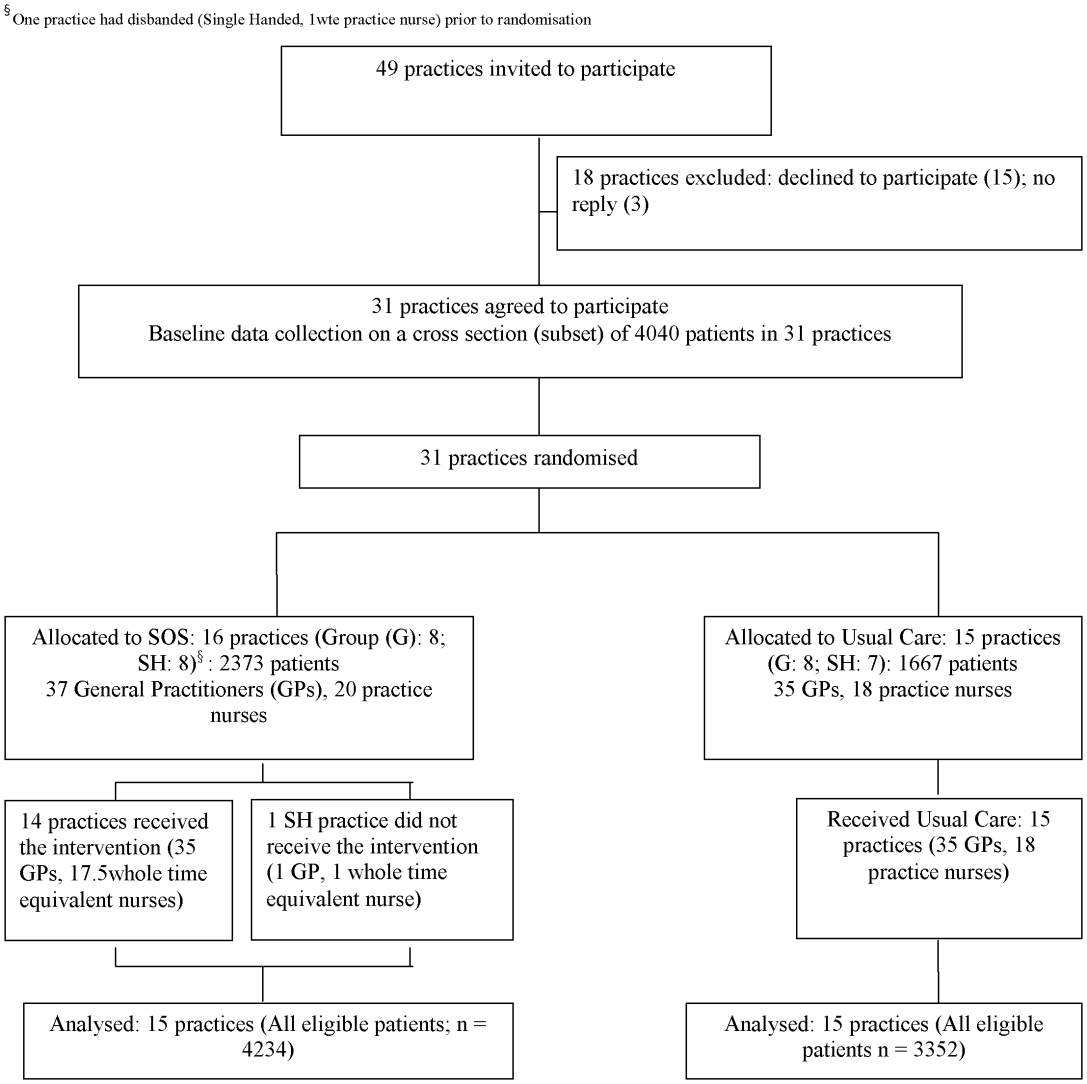
Trial profile.

Usual Care practices received no pharmacist support during the study. All practices in the study area including those participating, received a printed copy of local cholesterol/statin guidelines (File S1, Figure 1) at randomisation.

#### Outcomes

The primary outcome was the proportion of patients achieving the cholesterol target in the intervention group compared with the usual care group. Cholesterol targets were total cholesterol <5 mmol/l (except in patients with prior Coronary Artery Bypass Graft who were required to have cholesterol <4.2 mmol/l), in line with local (File S1, Figure 1) and United Kingdom audit standards [26], [34].

Secondary outcomes were: the proportion of patients prescribed simvastatin 40 mg with target cholesterol achieved; cholesterol levels; prescribing of simvastatin 40 mg; prescribing of any statin and the proportion of patients with cholesterol tested.

#### Measurement of outcomes

Two independent researchers who were blinded to treatment allocation, collected outcomes by extracting relevant electronic data from eligible patients’ electronic records in general practices (File S1, Page 29). Follow-up coincided with financial incentivisation for general practices to create and maintain accurate disease registers for patients with conditions including coronary heart disease or diabetes [33] and therefore data recording in practices’ computers had improved to the point where some practices were paperless and others were paper-light. Due to these improvements, at follow up, researchers were able to collect more patient outcome data during each visit to a practice, compared with baseline data collection. Follow up data were therefore collected for all 7586 eligible patients’ records in 30 general practices (Fig. 1 Trial profile), rather than from a subset as was the case at baseline. However, due to limited availability of opportunities to access practice computers, simultaneous collection of data from 30 general practices was not possible. Scheduling of different time slots for practice visits by researchers, led to different dates of follow up between pairs. Outcome data were collected for all eligible patients in 30 general practices between 1.4 and 2.2 years (mean 1.7 years) after randomisation. Data collection within each pair started in the same week, to ensure comparable follow up duration within pairs (File S1, Page 8).

Follow up analyses compared data in SOS with Usual Care practices. Monthly, practice level prescribing data is routinely collated across Scottish practices by the Information Statistics Division, Scotland and forwarded to local level for onward dissemination to practices. Simvastatin 40 mg prescribing data from participating practices were summarised from January 2003 through January 2013, to ascertain any long term intervention effects.

#### Sample size

Pilot work in five practices during 2002 found 50% of patients to have cholesterol targets achieved with an intraclass correlation coefficient (ICC) of 0.4. Ninety percent had cholesterol at target after the intervention. Pilot practices did not take part in the main study. Assuming that data for 40 patients per practice would be collected, we estimated that approximately 20 practices (10 per group) would be required to have 80% power, allowing for the cluster randomisation, to detect an increase in the proportion of patients with cholesterol at target to 90% in intervention practices.

#### Statistical analyses

Analysis was by intention to treat. Descriptive statistics for continuous variables are presented as mean and standard deviation, and for categorical variables as percentages. Due to the non-normal distribution of cholesterol levels, a log-transformation was applied; therefore geometric means describe cholesterol level outcome measures. The logged values for this measure are used for all analyses. Linear and logistic regression models were used to test for differences in the primary and secondary outcomes between SOS and Usual Care groups for continuous and binary outcomes respectively. Fixed effects models were adjusted for practice pair as a covariate to account for the matching at randomisation. Results are presented as intervention effect or odds ratio (OR), 95% confidence interval (CI) and p-value. Secondary analyses investigated the sensitivity of the results to adjustments for age and sex. ICCs are presented for all outcomes. A sensitivity analysis was carried out for the primary outcome, adjusting for any statin prescribing at follow up, to assess whether the treatment effect could be explained by increased statin prescribing.

Subgroup analyses were carried out according to the following variables: age; gender; practice-level socioeconomic deprivation; practice type (SH/G); patient type (prevalent or incident); number of co-morbidities (one vs. ≥2); ischaemic heart disease (IHD) comorbidity; statin prescribed/not prescribed and cholesterol at target or not, at baseline. Note that these baseline characteristics were defined by data collected at follow-up, since data collected before randomisation could not be linked to data collected at follow-up because matching required each patient’s Community Health Index number (CHI) and these were not collected at baseline. Subgroup analyses were performed by adding subgroup and the interaction between subgroup and treatment to the models described above. Results are presented graphically as the treatment effect (and corresponding 95% confidence interval) for each subgroup; the p-value for the interaction term is also presented.

We consider p-values less than 0.05 to be statistically significant. Data was managed and stored in the Robertson Centre for Biostatistics (RCB), which is part of the Glasgow Clinical Trials Unit and remains on the secure RCB network. Summarised data are available through the principal investigator. All analysis was carried out using SAS v9.1 for Windows (SAS Institute Inc, Cary, North Carolina, USA).

## Results

Recruitment secured the participation of 31 practices representing 12% (116,558) of the Greater Glasgow Health Board population. Sixteen practices (8 Group practices and 8 Single Handed practices) comprising 37 GPs and 20 nurses (19.5 whole time equivalents) were allocated to SOS. Fifteen practices (8 Group practices and 7 Single handed practices) comprising 35 GPs and 18 nurses were allocated to Usual Care. Figure 1 Trial profile, illustrates the flow of practices through the study. One SH practice had disbanded prior to randomisation, unknown to investigators at the time, leaving 15 practices allocated to the SOS arm. Of these 15 practices, 14 received the intervention.

We included all 31 practices in baseline analyses. Follow up analyses did not include the practice that had disbanded, leaving 30 practices. Practice level characteristics were comparable at baseline [29].

There were some differences at baseline between patients in SOS and patients in Usual care practices (Table 1). These differences were in the proportions of patients with Angina/Ischaemic Heart Disease; mean number of vascular co-morbidities; prescribing of any statin and optimal dose statin. The direction of the differences suggested it might be more difficult for the intervention to have an effect e.g. fewer patients in SOS practices were prescribed any statin and had their statin at optimal dose at baseline. One SH practice withdrew after the first meeting, but remained in the study, for analysis according to the intention to treat principle. The intervention was implemented as intended in 14 practices. At follow up, in response to the introduction of updated electronic patient records at the time of the new General Medical Services contract [33] we obtained data for all eligible patients (n = 7586) from all 30 participating practices: 4,234 patients were identified from 15 SOS practices and 3,352 patients from 15 Usual Care practices.

### Primary outcome

A greater proportion of patients in the SOS arm achieved cholesterol targets (2942/4234; 69.5% vs. 2130/3352; 63.5%, OR 1.11, 95% CI [1.00, 1.23]; p = 0.043. Table 2). ICCs were similar to those estimated from baseline data.

**Table 2:** Primary and secondary outcomes.

Outcome	SOS(15 practices; n = 4234 patients)	Usual Care(15 practices; n = 3352 patients)	ICC	Planned analysis		Age & sex adjusted	
				Treatment effect(95% CI)	P-value	Treatment effect(95% CI)	P-value
Cholesterol target achieved	2942 (69.5%)	2130 (63.5%)	0.005	1.11 (1.00, 1.23)	0.043	1.12 (1.01, 1.25)	0.027
Prescribed simvastatin 40 mg and targetcholesterol achieved	1898 (44.8%)	935 (27.9%)	0.024	1.78 (1.61, 1.98)	<0.001	1.81 (1.63, 2.01)	<0.001
Cholesterol level (mmol/l)*	4.22	4.36	0.038	0.98 (0.97, 0.99)	0.004	0.98 (0.97, 1.00)	0.006
Prescribed simvastatin 40 mg	2497 (59.0%)	1267 (37.8%)	0.025	2.06 (1.87, 2.28)	<0.001	2.12 (1.92, 2.34)	<0.001
Prescribed any statin	3682 (87.0%)	2509 (74.9%)	0.014	1.82 (1.60, 2.06)	<0.001	1.87 (1.65, 2.13)	<0.001
Cholesterol tested	3892 (91.9%)	2945 (87.9%)	0.050	1.30 (1.11, 1.54)	0.002	1.33 (1.13, 1.57)	<0.001

For binary outcomes, summaries are presented as number (percent) and treatment effects as odds ratios (SOS vs Usual Care) with corresponding 95% confidence intervals estimated from logistic regression models, adjusted for matched pairs. For continuous outcomes (*), summaries are presented as geometric means and treatment effects are presented as ratios (SOS vs Usual Care) with corresponding 95% confidence intervals estimated from linear regression models of the logged values, adjusted for matched pairs.

### Sensitivity analysis

The treatment effect was null after adjusting for any statin prescribing at follow-up (OR 1.00 [0.90, 1.11]; p = 0.89) suggesting the improvement in attainment of cholesterol targets in the SOS arm can be explained by the increase in statin prescribing.

### Secondary outcomes

All secondary outcomes were in favour of SOS. Simvastatin 40 mg prescribing was greater in SOS practices (2497/4234; 59.0%) than in usual care practices (1267/3352; 37.8%): OR 2.06 [1.87, 2.28]; p<0.001. Table 2). This SOS effect was statistically significant despite an increase in simvastatin 40 mg prescribing in Usual Care practices from 9.4% at baseline (Table 1) to 37.8% at follow-up. In SOS practices, the corresponding increase was from 8.9% (Table 1) to 59.0%.

All intervention effects were robust to adjustment for age and sex. Adjusting for practice level ratio and practice type, instead of adjusting for pair as covariate, gave similar results.

As planned, we have presented results from fixed effects models that do not explicitly account for the clustering of the data. Random (mixed) effects results were calculated, and due to the small amount of variation between practices, the results are similar to those from the fixed effects models (File S1, Page 21).

### Subgroup analysis

SOS practices performed better in most subgroups in relation to the primary and secondary outcomes (File S1, Pages 10–15).

### Prevalent and incident patients

At follow up, categorisation of patients as ‘incident’ or ‘prevalent’ enabled evaluation of any differential intervention effects. The intervention improved all outcomes in prevalent patients. The intervention appeared to be relatively less effective in incident compared with prevalent patients. Compared with incident patients in usual care practices, the improvement in prescribing and cholesterol for incident patients from SOS arm practices reached statistical significance in relation to four outcomes: prescribing of simvastatin 40 mg and target cholesterol achieved; cholesterol level; simvastatin 40 mg prescribing and prescribing of any statin. Incident patients in SOS arm practices also fared better than incident comparators in usual care, for the remaining two outcomes (cholesterol target achieved and cholesterol tested) but the extent of these improvements did not reach statistical significance (Table 3).

**Table 3:** Primary and secondary outcomes in incident and prevalent patients.

	Prevalent prescribing (30 practices; n = 5660 patients)				Incident prescribing (30 practices; n = 1926 patients)				P-value forinteraction
Outcome	SOS(15 practices: n = 3235)	Usual Care(15 practices: n = 2425)	Treatmenteffect (95% CI)	P-value	SOS(15 practices;n = 999)	Usual Care(15 practices;n = 927)	Treatmenteffect(95% CI)	P-value	
Cholesterol target achieved	2305 (71.3%)	1565 (64.5%)	1.19 (1.05, 1.34)	0.005	637 (63.8%)	565 (60.9%)	0.92 (0.76, 1.11)	0.381	0.025
Prescribed simvastatin 40 mg & targetcholesterol achieved	1485 (45.9%)	688 (28.4%)	1.89 (1.68, 2.13)	<0.001	414 (41.4%)	247 (26.6%)	1.51 (1.23–1.84)	<0.001	0.056
Cholesterol level (mmol/l)*	4.19	4.33	0.97 (0.96, 0.98)	<0.001	4.31	4.44	0.97 (0.95, 0.99)	0.006	0.920
Prescribed simvastatin 40 mg	1918 (59.3%)	923 (38.1%)	2.16 (1.92, 2.42)	<0.001	579 (58.0%)	344 (37.1%)	1.82 (1.50, 2.21)	<0.001	0.140
Prescribed any statin	2878 (89.0%)	1868 77.0%)	2.00 (1.72, 2.33)	<0.001	804 (80.5%)	641 (69.1%)	1.43 (1.14, 1.78)	0.002	0.013
Cholesterol tested	3008 (93.0%)	2137 (88.1%)	1.58 (1.30, 1.92)	<0.001	884 (88.5%)	808 (87.2%)	0.80 (0.60, 1.08)	0.140	<0.001

For binary outcomes, summaries are presented as number (percent) and treatment effects as odds ratios (SOS vs Usual Care) with corresponding 95% confidence intervals estimated from logistic regression models, adjusted for matched pairs. For continuous outcomes (*), summaries are presented as geometric means and treatment effects are presented as ratios (SOS vs Usual Care) with corresponding 95% confidence intervals estimated from linear regression models of the logged values, adjusted for matched pairs.

### Variation in outcomes in SOS versus usual care

Across SOS practices, the percentage of patients with Simvastatin 40 mg achieving cholesterol targets at follow up ranged from 27.4% to 56.5% compared with 6.45 to 55.6% in Usual Care practices. This finding of a smaller range of outcome values in SOS practices was noted in all other outcomes (Table 4).

**Table 4:** Range of primary and secondary outcomes.

Outcome	SOS (15 practices; n = 4234 patients)N (%; range)†	Usual care (15 practices; n = 3352 patients)N (%; range) †
Cholesterol target achieved	2942 (69.5%; 49.0–77.5%)	2130 (63.5%; 35.6–83.4%)
Simvastatin 40 mg and cholesterol target achieved	1898 (44.8%; 27.4–56.5%)	935 (27.9%; 6.4–55.6%)
Cholesterol level (mmol/l)	4.22 (4.03–4.49)	4.36 (3.82–4.95)
Prescribed simvastatin 40 mg	2497 (59.0%; 30.7–71.8%)	1267 (37.8%; 15.2–65.7%)
Prescribed any statin	3682 (87.0%; 68.0–93.7%)	2509 (74.9%; 47.1–93.8%)
Cholesterol tested	3892 (91.9%; 73.3–98.8%)	2945 (87.9%; 65.8–97.4%)

† Cholesterol level given as geometric mean (range) mmol/l.

### Long term follow up with routine data

**Figure 2:**
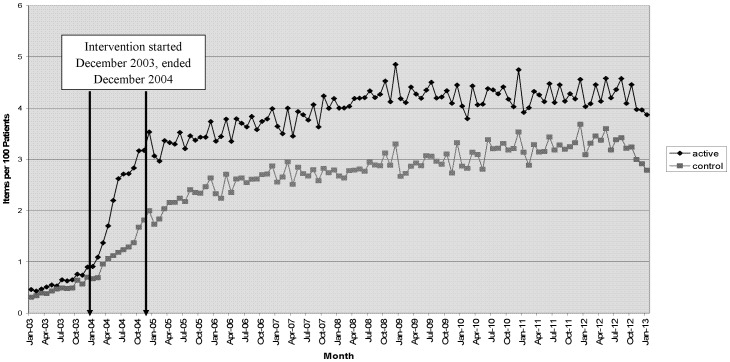
Long term Simvastatin 40 mg prescribing in Intervention vs. Usual Care practices.

Simvastatin 40 mg prescribing data (for all patients, with or without atherosclerotic disease) was available for each practice on a monthly basis from January 2003 to January 2013 (Figure 2 Long term Simvaststin 40 mg prescribing). Despite an upward trend in prescribing during the intervention period, prescribing in SOS practices is seen to increase on commencement of the intervention, and the difference persisted up to 10 years later.

## Interpretation

Pharmacists’ practice-level educational and organisational intervention lowered the risk of atherosclerotic events by increasing the proportion of patients achieving target cholesterol levels, and improving statin prescribing. The intervention effect persisted despite increases in statin prescribing during the study period. Similar increases were noted in other healthcare systems over the same period [24], [66].

In considering whether statin prescribing improvements were key to the primary outcome being favourable, we adjusted for statin prescribing as a covariate at follow up. When we did this, the treatment effect was neutralised, suggesting the difference observed in the primary outcome was driven mainly by the prescribing of statins. There are other plausible although less likely explanations for the favourable primary outcome: practices in the SOS arm may have responded more positively to the General Medical Services (GMS) contract Quality and Outcomes Framework which asked all practices to create a register of patients with CHD, Stroke and Diabetes and manage patients’ cholesterol without any influence from the pharmacist intervention [34]; or patients in SOS practices may have developed improved statin adherence during the study period.

The results show practices in the SOS arm had significantly improved prescribing for incident patients, but not achievement of cholesterol targets. Looking at the secondary outcomes, this could be due to the lack of intervention effect on having cholesterol tested, which may reflect the shorter time period that the intervention could affect the management of the incident patients.

We randomly allocated practices to SOS or Usual Care while maintaining blinding, to minimise the chance of performance and detection bias. However, we could not ensure ‘quadruple blinding’ [77] because practices were notified of their allocation in writing. Performance bias was therefore possible through usual care practices trying harder to achieve the outcomes for which their SOS group counterparts received support. Patients remained blinded to allocation throughout the study, because there was no requirement for written consent at patient level and the intervention was mediated through practices. The Hawthorne effect was unavoidable for participating GPs and Nurses, as is the case in all randomised controlled trials involving an educational component that cannot be masked by design.

Patients in the SOS arm had lower cholesterol levels at follow up, suggesting adherence to prescribing changes, despite a known tendency for non-adherence in the first year of prescribing and following dose-intensification [56], [57]. In recommending Simvastatin 40 mg for patients regardless of cholesterol levels, SOS accommodates a ‘fire and forget’ management strategy for a fixed dose, generically available statin [58]. We did not collect information on clinical events in view of the established and robust link between cholesterol lowering, simvastatin prescribing and reduced vascular events [15], [16]. However, the difference in simvastatin 40 mg prescribing (59% SOS vs. 38% usual care (Table 2) approximates to the number needed to treat for 5 years, to prevent one vascular event [15].

Since this study concluded, multinational surveys have consistently shown clinically important variations in patient identification, prescribing, dosing and lack of attainment of cholesterol targets [21]–[24], [59]. In UK general practice, due to features of the pay-for performance contract such as threshold targets (after reaching upper payment thresholds for a defined proportion of the eligible population, practices do not receive additional payments for target achievement in additional patients) and exception reporting (practices can except (exclude) patients from payment denominators, due to a variety of reasons e.g. no response to invitations to attend the practice) [27], uncertainty exists over whether target cholesterol is achieved in approximately 20% of eligible patients [60]. These features of the UK contract underscore the need to develop and test collaborative interventions aiming to increase the uptake of evidence based practice. As far as we are aware, our results provide, for the first time, empirical evidence of patient [9] or healthcare professional [10] level pharmacist-led interventions generating improvements in disease markers or prescribing for longer than one year [6], [61]–[65].

### Differences between SOS study and other work

Previous work has shown pharmacists’ patient facing interventions are capable of lowering cholesterol levels [66], [70], [71] or improving the prescription of lipid lowering medicines [72] but we are not aware of reports of improved surrogate clinical outcomes from pharmacist intervention targeted at general practitioners.

The number of patients included was larger than previous pharmacy led intervention studies. Follow up was longer than in previous studies of this kind [11], [12] which gives some assurance that changes made in the study, had a lasting effect although incident patients in SOS practices did not fare any better than those in usual care practices in relation to the primary outcome. Measuring outcomes over the long term is important for patients with vascular disease because clinical benefits from statin use are accrued over the long term [15], [73]. As far as we are aware, follow up in previous educational outreach type research was up to 12 months [72], [74] and there are no previous reports of an effect possibly lasting up to 10 years (Figure 2).

### Strengths

The size of the treatment effect in previous studies involving pharmacists delivering academic detailing/educational outreach type interventions are generally lower than our finding [1], [3], [5], [11], [35], [36]. SOS may have had positive outcomes because of the relatively narrow focus on statins (Simvastatin 40 mg in particular) and cholesterol management for patients with vascular disease that enabled GPs and the practice team to identify and follow up a defined group of patients with a specific intervention. Other investigators have delivered a broader spectrum of educational messages with mixed success e.g. hypertension [37]; antibiotic prescribing for acute conditions [38]–[43]; multiple prescribing topics [6]; and potentially inappropriate prescriptions [44].

Prolonged contact time and regular, repeated visits enabled pharmacists to develop working relationships with practice staff, understand individual GP, Nurse and practice’s needs and then provide individual educational and organisational support accordingly. Tailored interventions are thought to be powerful predictors of effectiveness [45], [46], and fewer contacts are thought to predict lower levels of success [47]. Key educational messages were repeated and pharmacists facilitated change whenever this was possible. These features of the SOS intervention can be aligned to markers of quality in patient education interventions [48]. The combination of organisational and educational support is likely to have been synergistic. Organisational support included providing practices with a list of recommendations for each patient who was found to be sub-optimally managed, an approach previously found to be successful [49], [50] while others have cited an inability to address organisational barriers as one reason for a neutral result [51]. Collaboration and repeated intensive support to identify and follow up eligible patients, together with timely communication, are also likely to have been important features [8], [10]. SOS intervention comprised weekly visits to practices over one year, which exceeds the number of contacts studied previously. Soumerai observed an approximate doubling of the magnitude of changes to targeted medicines when the number of visits doubled [52] while others have suggested a minimum of three visits to enable change to occur [53]. The Cochrane collaboration considered the impact of the number of visits on the success of academic detailing and found a wide range, from once weekly for seven months [54] to single visits [55] with examples of successful and unsuccessful outcomes across the spectrum [3].

The intervention was delivered as planned and effects lasted longer than those seen in previous pharmacist led educational intervention trials [4], and case management approaches [66]. SOS was delivered by pharmacists who had minimal additional training. Participating practices were representative of practices in the largest Health Board area in Scotland [29]. More pharmacists delivered the intervention than in previous educational outreach or organisational level interventions targeting cardiovascular disease management. Data were collected from a greater number of patients than in any previous complex intervention trial involving pharmacists and prescribing [3], [5], [11], [35]. The clinical profiles of eligible patients were similar to participants in landmark statin trials and surveys (File S1, Pages 23 and 24). Optimal dose statin initiation and cholesterol lowering are of proven benefit for patients with established vascular disease [15], [16]. Sub-optimal management of cholesterol and sub-optimal statin prescribing are common [67], [68] and the SOS intervention may offer a means of addressing this public health need. In contrast with previous pharmacist led trials [2], [6], [35], [66], we did not select practices on the basis of their capacity to benefit from the intervention, or only include patients who had capacity to benefit, in our denominators. By randomly selecting practices for inclusion in the trial, we recruited a representative sample from Scotland’s largest health board, and a large patient cohort to maximise transferability. Together, these features support implementation in healthcare systems with sub-optimal statin prescribing and cholesterol management [19]–[25].

The SOS effect appears to be greater in practices where baseline levels of statin prescribing and cholesterol target achievement were lowest (File S1, Pages 15–20). The intervention supported the least well-performing practices to achieve a higher level of performance but did not boost performance of the top practices beyond what they could achieve without the intervention. In addition, variation in outcomes was minimised at follow up in SOS practices compared to usual care (File S1, Pages 15–20). This result may be due to the systematic approach to call and recall introduced by pharmacists, ensuring all patients were contacted by their practice on several occasions, by letter, phone or opportunistically and offered a statin or cholesterol test. In this respect, because of the additional time spent working through lists of patients, the pharmacists may have enabled practices to reach patients who had previously not responded to written invitations. SOS may be more appropriately used in practices with outlying (low levels) of statin prescribing and cholesterol target achievement. Because of the volume of work involved, usual care practices were unlikely to have had the time to adopt this approach, and the GMS contract does not incentivise the additional work involved in persistently following up patients to increase uptake of statins or other interventions among patients who do not respond to initial invitations [34], [60].

### Limitations

The directions of baseline imbalances in two variables which were related to the outcome (a reduced proportion of SOS arm patients prescribed a statin and a reduced proportion prescribed an optimal dose) are likely to have led to there being a greater burden of work for pharmacists and practices in the SOS arm than for practices in the Usual Care arm. The additional work comes from there being proportionately more patients requiring changes to their statin prescribing and cholesterol management, for tasks such as patient identification, engagement and appointment booking, prescribing, test ordering and booking follow up appointments. Pharmacists supported practices to carry out all of these tasks, and as SOS practices had a proportionately greater burden at baseline, additional work was required to overcome the burden. If practices were balanced at baseline in relation to these variables, the pharmacists would have had less of these tasks to address, and more time to focus on other tasks e.g. providing relevant educational support to GPs.

The clinical significance of the finding that achievement of cholesterol targets was not statistically significantly greater in incident SOS practices is unclear, given that cholesterol lowering may not form a necessary part of the causal chain between statin prescribing, consumption and improved clinical outcomes [15], [58]. However, recent UK consensus recommendations suggest statins should be prescribed to achieve values of at least <2.5 mmol/l for non HDL cholesterol [78]. Any improvement in clinical outcomes resulting from this study are likely to be conditional on improved statin prescribing persisting in both prevalent and incident patients over the long term.

Baseline cholesterol data was only collected for a sample of the prevalent patients at this time. The method of data collection did not allow for linkage to follow-up data, therefore we did not have baseline cholesterol levels for all prevalent patients identified at follow-up.

As with other trials of complex, multifaceted interventions, the effective components remain unknown without testing each component individually [35], [75].

Due to improvements in statin prescribing since the study completed, if implemented today, to retain effect size, the intervention may need to be targeted at underperforming practices rather than a random selection.

We did not conduct an economic analysis and therefore the policy cost-effectiveness of the SOS intervention remains unknown. The cost of delivering the intervention over a single year could be weighed against any longer term washover effect on statin prescribing, and a comparison made with usual care (in the UK) where general practices are financially incentivised annually for continuing to achieve targets for prevalent and incident patients. Previous economic analyses of pharmacist led outreach interventions have shown mixed results [6], [76].

By design, we did not track individual patients from baseline to follow up, which precluded interpretation of results relative to pre-intervention characteristics in the same patients. Follow up analysis did not adjust for baseline differences however, these were in a direction that acted against the intervention effect, and so would not be expected to change findings. Improved achievement of cholesterol targets at follow up in the intervention group may have resulted from a combination of patients becoming more adherent with their statins and more patients receiving statins and retaining the same level of adherence. A greater proportion of SOS group patients were prescribed optimal dose statins, which suggests the latter, but we did not capture data on adherence which limits interpretation of the impact of the intervention on patients’ behaviour.

It is possible that other healthcare professionals could deliver SOS, and the model could generate improved outcomes in other therapeutic areas, but these hypotheses require further testing. The possibility of a null effect in the primary outcome cannot be ignored because of the narrow difference between the observed p-value and the a-priori threshold value of 0.05, and because the lower limit of the confidence interval included 1.00. However, all other outcomes were statistically significantly different and in a direction which favours the intervention. In particular, cholesterol levels were reduced in the SOS group, indicating some degree of change in practice by patients.

In 2004, coinciding with the study commencing, UK general practitioners’ national contract was revised to financially incentivise attainment of evidence based clinical quality indicators including statin prescribing and cholesterol targets [33]. While this raised practices’ awareness of the need to address this area, the effect applied equally to all participating practices. Outcomes remained statistically significant despite confounding introduced by the UK pay-for-performance contract.

## Conclusions

Given the challenges of an ageing population with multiple long term conditions and sub-optimal prescribing, healthcare providers require effective approaches to improve prescribing and therapeutic targets that are effective over the long-term. Morbidity and mortality from cardiovascular disease remains high, and statin prescribing remains sub-optimal. SOS offers a pragmatic model for improving long term evidence-based statin prescribing, and attainment of cholesterol targets, in a high risk population.

## Supporting Information

File S1 **Additional information on intervention, analyses, comparison with previous work.**
(DOCX)

Checklist S1 **CONSORT 2010 checklist of information to include when reporting a cluster randomised trial.**
(DOCX)

Protocol S1 **Study protocol.**
(DOC)
