# Supplementary material for: A Cluster Randomised Controlled Trial of a Pharmacist-Led Collaborative Intervention to Improve Statin Prescribing and Attainment of Cholesterol Targets in Primary Care
Source: PLoS One. 2014 Nov 18;9(11):e113370. doi: 10.1371/journal.pone.0113370 (PMC4236200; doi:10.1371/journal.pone.0113370)
Supplement: File S1 — Additional information on intervention, analyses, comparison with previous work. (DOCX) [file pone.0113370.s001.docx]

**Supporting information File S1**

**Table of Contents**

1. SOS intervention………………………….…………………….…………………………….....3
2. Pharmacists’ training………………………………………………………………………..…..5
3. Statin prescribing guideline……………………………………………………………………..7
4. Duration of follow up by practice pairs.................................................……………………........8
5. Subgroup analyses of the primary outcome……..……………………………………………...9
6. Subgroup analyses of secondary outcomes………………………………………….… ….….11
7. Progression from baseline to follow up in outcomes, by practice……… …………….….…..16

8. Primary and secondary outcomes (mixed effects)......................................................................21

9. Comparison of SOS baseline characteristics with landmark statin trials……………………....22

10. Comparison of SOS baseline characteristics with statin surveys………………………………23

11. Comparison of Simvastatin 40mg prescribing across Scotland, within the study period...........24

12. Comparison of % Simvastatin 40mg prescribing (as a proportion of all statins)………………25

13. Patient level baseline and outcome Clinical Research Forms………………………………….26

14. References……………………………………………………………………………………...30

1. **SOS intervention**

**Educational outreach**

Educational Outreach is defined as a personal visit by a trained person to healthcare professionals in their own settings.^1^ It commonly incorporates the provision of feedback on prescribing issues. The following key features are described in previous work, based upon a social marketing framework:^2^

- Focussing on a targeted, small group of clinicians;
- Defining clear educational and behavioural objectives;
- Establishing credibility;
- Stimulating active participation;
- Using concise graphic educational material;
- Highlighting and repeating essential messages;
- Providing positive reinforcement in follow up visits.

The pharmacists delivering the SOS intervention adhered to these principles during their discussion time with GPs and Nurses. Three face to face meetings between the pharmacist and GP or Nurse, were scheduled to take place over one year.

The first (one to one) meeting explored current statin prescribing habits, awareness of the evidence from landmark statin trials including the Heart Protection Study (HPS) and an understanding of statin effectiveness. The pharmacist facilitated agreement within each practice, on the potential benefits of improving statin prescribing and the usefulness of introducing simvastatin 40mg for patients fulfilling the entry criteria of the HPS and local guidelines. The opinions of local cardiologists or biochemists were sought by the pharmacist if the GP or nurse had questions that could not be answered by the pharmacist or the research team.

In the second meeting (four months after the first, involving all GPs and nurses together), the pharmacist used information gathered during the first meeting to prepare and deliver an interactive presentation. The presentation covered the evidence base for prescribing simvastatin 40mg for patients with vascular disease together with a recommendation (specific to each practice and each GP or nurse) about how the pharmacist could help to systematically identify and offer simvastatin 40mg to eligible patients.

Twelve months after the first meeting, the pharmacist met face to face with each GP and nurse. This enabled feedback of changes made at individual and practice level in the previous months. Agreement was reached on how the practice could sustain change and ensure eligible incident (newly diagnosed) patients could receive the offer of simvastatin 40mg. Evidence based statin prescribing and dosing was encouraged through repetition and reinforcement of key learning points.

**Organisational support**

Practical support for case finding and call/recall was included in the SOS intervention because of the inevitable increase in workload associated with the pharmacist identifying unmet prescribing need, on top of a background of increased workload in general practices. Recommendations related to statin initiation/dose titration or ordering of overdue laboratory tests for lipids or liver function tests (Table 1). Recommendations were passed to all GPs for approval. Practices were free to choose to prescribe a statin other than simvastatin e.g. atorvastatin, pravastatin or fluvastatin, but these choices were countered in the public interest, by the pharmacist describing the lower cost of simvastatin and greater weight of evidence.

If recommendations were agreed, the pharmacist supported implementation by drafting a letter (individualised for each patient), with the GP’s signature for issue by the practice. The letter communicated the need, nature and reasons for any change to prescribing or additional blood tests. Recommendations were followed up systematically for each patient, by the pharmacist, collaborating with practice staff in their usual approach to call/recall, to ensure implementation. This included the pharmacist triaging and reassessing changes to each plan over time if necessary. The pharmacist provided help to schedule appointments for patients with GPs or practice nurses and updated practice disease registers as appropriate. By providing summaries of progress and reminders, the pharmacist helped practice staff to track non attendees by letter, phone or opportunistically. This approach aimed to minimize dropout and maximise uptake, aiming to counter anticipated high non-attendance at appointments for preventative interventions, particularly in areas of socioeconomic deprivation. ^ref^ Pharmacists encouraged practice staff to telephone patients if there was no response to initial letters or provide scheduled appointments for non attendees. Practices were encouraged to sustain this systematic approach for incident patients.

**Table 1. Summary of pharmacists’ recommendations to practices**

| **Category of patient with vascular disease** | **Recommended intervention by nurse or GP** |
| --- | --- |
| Prescribed low potency^§^ statin, cholesterol and LFTs known and at target | Switch to simvastatin 40mg by contacting patient on phone then confirming by letter |
| Prescribed low potency statin, cholesterol and LFTs not known/not at target | Letter for blood test then switch to simvastatin 40mg |
| Prescribed potent statin, cholesterol not at target | Phone call to discuss concordance |
| Prescribed potent statin, cholesterol and LFTs not known/not at target | Letter for blood test then increase dose if necessary |
| Prescribed potent statin, cholesterol at target | No action |
| Prescribed low potency statin, cholesterol at target | No action *  Check LFTs |
| No statin; LFTs and cholesterol known | Letter for appointment for consideration of statin |
| No statin, LFTs and cholesterol not known | Letter for appointment for bloods then discussion of need (GP/nurse), leading to statin prescription |
| Started on simvastatin 40mg (or other sufficiently potent statin), cholesterol not known | Letter for appointment for bloods |

§ Potency relates to the cholesterol lowering ability of the statin. Low potency statins include Fluvastatin and Pravastatin. Potent statins are all others e.g. simvastatin, Atorvastatin, Rosuvastatin. * Some practices decided, on the weight of the HPS evidence base, to substitute low potency statins with simvastatin. LFTs = Liver Function Tests

1. **Pharmacists’ training**

Based on their availability over the duration of the study, 11 prescribing support, practice-attached pharmacists were selected from 23 employed by NHS GG&C in 2003. All worked in general practices as prescribing support pharmacists, on a full time or part time basis, shared the same job description, and were remunerated at the same level under NHS terms and conditions. Key characteristics are given in Table 2.

**Table 2. Pharmacists’ characteristics**

| **Pharmacist**  **(Male (M) / Female (F)** | **Number of years post qualification** | **Number of years prescribing support experience*** | **Role prior to general practice prescribing support** | **Postgraduate clinical pharmacy qualification** | **Part time (PT) / Full Time (FT)** |
| --- | --- | --- | --- | --- | --- |
| #1 (M) | 5 | 4 | Community Pharmacy | Y | FT |
| #2 (F) | 2 | 1 | Community Pharmacy | N | FT |
| #3 (F) | 7 | 4 | Community Pharmacy | N | PT (0.6wte) |
| #4 (F) | 7 | 4 | Community Pharmacy | N | FT |
| #5 (M) | 4 | 1 | Community Pharmacy | N | PT (0.2wte) |
| #6 (F) | 5 | 4 | Community Pharmacy | N | PT (0.6wte) |
| #7 (F) | 15 | 3 | Community Pharmacy and Hospital pharmacy (Psychiatry) | Y | PT (0.4wte) |
| #8 (F) | 13 | 3 | Community Pharmacy | N | FT |
| #9 (F) | 6 | 3 | Academia | Y | PT (0.8wte) |
| #10 (F) | 6 | 3 | Community Pharmacy | N | PT (0.6wte) |
| #11 (F) | 12 | 4 | Community Pharmacy | N | FT |

* experience was mainly clinical medication review based in general practices, for patients receiving polypharmacy.

To ensure a minimum level of knowledge and skills tailored to the SOS intervention, all pharmacists attended six and a half training days between September 2003 and January 2004. Pre-requisites for delivering the SOS intervention were agreed amongst the pharmacists and included:

- Attendance at all of the study days and achievement of a satisfactory standard (of motivation and performance; assessed by the principle investigator) during each training day;
- Commitment to delivering the SOS intervention in the following 12 months.

Forty one contact hours of training were accrued by each pharmacist. The training was specific to the delivery of the three meeting approach to the SOS intervention. A mixture of academic GPs, GPs experienced in postgraduate education, a consultant cardiologist, a cardiac nurse and a senior lecturer delivered the training sessions. Training covered the following topics:

- Evidence base for use of statins;
- Educational outreach;
- Study protocol;
- Pharmacological actions of statins;
- Aetiology of vascular disease;
- Primary/secondary prevention thresholds;
- Communication skills required to deliver the SOS intervention through three, linked meetings;
- Practitioner and patient level barriers to systematic uptake of simvastatin prescribing;
- Adult learning theory.

All pharmacists were competent in the operation and searching of practice computer systems. All 11 pharmacists attended the training events and performed satisfactorily. None of those recruited had previously delivered the intervention.

**3. Statin prescribing guideline (NHS Greater Glasgow and Clyde)**

## Fig 1. Secondary prevention of coronary heart disease and stroke

##### Patients with established atherosclerotic disease are at high risk and should be treated with a statin regardless of total blood cholesterol concentration

##### i.e. Previous MI / pre- or post-CABG / pre- or post-Angioplasty / Angina / IHD / Angiographic coronary artery disease / Ischaemic stroke or TIA / Peripheral Arterial Disease / Patients with diabetes aged ≥ 40 years

**^‡^ *Consider referral to lipid clinic in resistant cases, if liver transaminases > 2x normal, or if statin not tolerated.***

^†^ *^Discuss with lipid clinic if in doubt^*

**Do within 24 hours of onset of acute MI*

Random non-fasting test for total cholesterol* and LFT’s

Consider secondary causes & familial hyperlipidaemia if Cholesterol >8.0 mmol/L^†^

***See BNF for cautions, contra-indications and clinically important interactions.***

# Re-test at 1 month

Random non-fasting total cholesterol + triglycerides + LFT’s

# Goals of Treatment by Three Months

# Total cholesterol concentration <5.00 mmol/l

# (<4.2 mmol/L for post CABG patients)

# and

# Reduce cholesterol concentration by ≥ 25%

Cholesterol goals not achieved Discuss concordance. Switch to Atorvastatin 40 – 80mg depending on response. Consider addition of ezetemibe. The use of other lipid-lowering agents is not recommended

Trigs>4.0mmol/l Check fasting sample^‡^

Cholesterol Goals Achieved

Annual review to ensure continued concordance.

**Treat all patients with statin regardless of baseline cholesterol concentration**

**Recommended drug & daily dose**

**Simvastatin 40mg daily**

Consider **Atorvastatin 80mg** in acute coronary syndrome with elevated troponin

**Table 3. Duration of follow up by practice pairs**

| Practice (G = Group / S = Single Handed) | Active / control | Pair | Duration of follow up for primary and secondary endpoints (yrs) † |
| --- | --- | --- | --- |
| G | Intervention | 1a | 1.4 |
| G | Usual care | 1c |  |
| G | Intervention | 2a | 1.7 |
| G | Usual care | 2c |  |
| G | Intervention | 3a | 1.5 |
| G | Usual care | 3c |  |
| G | Intervention | 4a | 1.8 |
| G | Usual care | 4c |  |
| G | Intervention | 5a | 1.4 |
| G | Usual care | 5c |  |
| G | Intervention | 6a | 2.1 |
| G | Usual care | 6c |  |
| G | Intervention | 7a | 1.8 |
| G | Usual care | 7c |  |
| G | Intervention | 8a | 1.4 |
| G | Usual care | 8c |  |
| S | Usual care | 1c | 1.6 |
| S | Intervention | 1a |  |
| S | Intervention | 2a | 1.4 |
| S | Usual care | 2c |  |
| S | Intervention | 3a | 1.7 |
| S | Usual care | 3c |  |
| S | Intervention | 4a | 1.4 |
| S | Usual care | 4c |  |
| S | Intervention | 5a | 2.2 |
| S | Usual care | 5c |  |
| S | Intervention | 6a | 1.5 |
| S | Usual care | 6c |  |
| S | Intervention | 7a | 2.2 |
| S | Usual care | 7c |  |
| Mean (range): 1.7years (1.4years – 2.2years) | | | |

^†^ period between randomisation (4^th^ November 2003) and start date of follow up data collection in each pair

**Fig 2.**

**5. Subgroup analyses of primary outcome**

**Fig 3.**

**6. Subgroup analyses of secondary outcomes**

**Fig 4.**

**Fig 5.**

**Fig 6.**

**Fig 7.**

**7. Progression from baseline to follow up in outcomes, by practice**

**Figure 9.**

**Table 4. Primary and secondary outcomes (mixed effects)**

| **Outcome** | **SOS**  **(n = 4,234)** | **Usual Care**  **(n = 3,352)** | **ICC** | **Planned analysis** | | **Age & sex adjusted** | |
| --- | --- | --- | --- | --- | --- | --- | --- |
|  |  |  |  | **Treatment effect**  **(95% CI)** | **p-value** | **Treatment effect**  **(95% CI)** | **p-value** |
| Cholesterol target achieved | 2,942 (69.5%) | 2,130 (63.5%) | 0.005 | 1.13 (1.02, 1.25) | 0.020 | 1.14 (1.03, 1.26) | 0.012 |
| Prescribed simvastatin 40mg and target cholesterol achieved | 1,898 (44.8%) | 935 (27.9%) | 0.024 | 1.80 (1.63, 2.00) | < 0.001 | 1.83 (1.65, 2.03) | < 0.001 |
| Cholesterol level (mmol/l)* | 4.22 | 4.36 | 0.038 | 0.98 (0.97, 0.99) | 0.002 | 0.98 (0.97, 0.99) | 0.002 |
| Prescribed simvastatin 40mg | 2,497 (59.0%) | 1,267 (37.8%) | 0.025 | 2.08 (1.88, 2.30) | < 0.001 | 2.13 (1.93, 2.36) | < 0.001 |
| Prescribed any statin | 3682 (87.0%) | 2509 (74.9%) | 0.014 | 1.84 (1.62, 2.08) | < 0.001 | 1.89 (1.66, 2.14) | < 0.001 |
| Cholesterol tested | 3,892 (91.9%) | 2,945 (87.9%) | 0.050 | 1.31 (1.12, 1.55) | 0.001 | 1.34 (1.13, 1.57) | < 0.001 |

For binary outcomes, summaries are presented as number (percent) and treatment effects as odds ratios (SOS vs Usual Care) with corresponding 95% confidence intervals estimated from mixed logistic regression models, adjusted for matched pairs as a random effect.For continuous outcomes (*), summaries are presented as geometric means and treatment effects are presented as ratios (SOS vs Usual Care) with corresponding 95% confidence intervals estimated from mixed linear regression models of the logged values, adjusted for matched pairs as a random effect.

**9. Comparison of baseline characteristics with landmark statin trials**

**Table 5. Baseline clinical characteristics of patients in the SOS study compared with landmark clinical trials of statins**

|  | **SOS** | **PROSPER** | **CARE** | **LIPID** | **HPS** |
| --- | --- | --- | --- | --- | --- |
|  | Vascular disease | Vascular disease / multiple risk factors | MI | CHD | Vascular disease |
| n | 4,040 | 5,804 | 4,159 | 9,014 | 20,536 |
| Mean follow up (years) | 1.7 | 3.2 | 5 | 6.1 | 5 |
| **Patient characteristics** |  |  |  |  |  |
| Age (yr) | 68 | 75 | 59 | 62 | 40 – 80^×^ |
| Sex (male) % | 70 | 48 | 86 | 83 | 75 |
| *Physiological measures*  Mean Cholesterol (mmol/l)* | 5.1 | 5.7 | 4.5^§^ | 5.8 | 5.9 |
| *Co-morbidities (%)*  Angina/IHD  MI  Stroke  TIA  PVD  Diabetes  CABG  Angioplasty | 46  21  14  9  11  36  9  4 | 27  13  9  17  9  11  6  4 | 21  100  -  -  -  14  27  34 | 36  64  4  4  10  9  27  11 | 24  41  16^†^  ^-^  33  29  -  - |

^†^ combined with TIA ^‡^ combined with angioplasty § entry criteria < 4.5mmol/l * statin free ^×^46%

**Table 6. Baseline clinical characteristics of patients in the SOS study compared with statin surveys**

**10. Comparison of SOS baseline characteristics with statin surveys**

|  | **SOS**  **(2003/4)** | **EUROASPIRE**  **I (1995/6)** | **EUROASPIRE II (1999/00)** | **ASPIRE**  **(1994/5)** | **L –TAP**  **(1996/7)** |
| --- | --- | --- | --- | --- | --- |
| n | 4040 | 3569 | 3379 | 2583 | 4888 |
| Location | Scotland | Europe (excluding UK) | Europe (excluding UK) | UK | North America |
| Patient characteristics | MI, CHD, Angioplasty, CABG, Diabetes, PVD, Stroke, TIA | MI, CHD, Angioplasty or CABG | MI, CHD, Angioplasty or CABG | MI, CHD, Angioplasty or CABG | Hyperlipidaemic^§^ |
| Cholesterol with statin | 4.75 | **-** | **-** | **-** | **-** |
| Cholesterol without statin | 5.10 | **-** | **-** | **-** | **-** |
| *Cholesterol above target range (%) ^§^* | 61 | 86 | 59 | 78 | 82 |
| *Any lipid lowering (%)* | 41 | 32 | 63 | 10 | 27 |
| *Any statin (%)* | 41 | 19 | 58 | **-** | 21 |
| *Any statin, any dose, below target cholesterol (%)* | 21 | 21 | 49 | < 5 | 39 |

^§^ Data shown for CHD participants only

**11. Comparison of Simvastatin 40mg prescribing across Scottish Health Board areas, within the study period**

**Figure 14.**

**12. Comparison of % Simvastatin 40mg prescribing (as a proportion of all statins) in intervention vs control practices**

**Figure 15**

**13. Clinical Research form***

**Practice ID: …………………………… Today’s date:……………………………………**

**Patient I.D.: …………………………… DOB: ……………… M / F**

**1. Relevant Diagnoses**

| **Secondary prevention** | **definite** | **possible** | **Primary Prevention** | **yes** | **no** | **unclear** |
| --- | --- | --- | --- | --- | --- | --- |
| Previous MI |  |  | Hypertension: Syst >160 or Diast >90 or on antihypertensive therapy |  |  |  |
| Pre/Post CABG |  |  |  |  |  |  |
| Pre/Post Angioplasty |  |  | FH of CHD: Father <45yrs or Mother <55yrs |  |  |  |
| Angina / IHD |  |  |  |  |  |  |
| PVD/ Intermittent claudication |  |  | Smoker |  |  |  |
| Diabetes |  |  | Other (specify): | | | |
| Stroke |  |  |  |  |  |  |
| TIA |  |  |  |  |  |  |

**Statin prescription…**

| **Statin:** | **Dose** | **Current Cholesterol level :**  **………………mmol/l (LDL ….….. ...........HDL…………….)**  **Date……………………………………………**  **or**  **None** |
| --- | --- | --- |
| None |  |  |
| Simvastatin | 10mg |  |
| Pravastatin | 20mg |  |
| Atorvastatin | 40mg |  |
| Fluvastatin | 80mg |  |
| Other: …………………. | Other: …………… |  |

*Clinical Research Form used at follow up differed from this by checkbox asking whether patient was new to practice since date of baseline data collection.

All data was transcribed directly onto an ACCESS database

**Note on baseline and follow up data collection**

In the UK, prescriptions in general practices’ computer systems are categorised and filed as either ‘acute’ (intended as a single, one-off treatment issue for sufficient quantity to last one month; not to be repeated; in response to the patient presenting with an acute, short lived problem) or ‘repeat’ (a medicine for a long term condition which is issued every month, automatically, by the practice, for the patient to collect then redeem at a community pharmacy). GPs initiate and maintain these records. Each entry represents at least one month’s supply of the medicine, as the repeat prescribing cycle is monthly, with the practice automatically issuing and the patient collecting their repeat prescription every month. As is the case on initiation of other long term medicines, the first statin prescription usually appears on the acute file. After the patient collects and takes the statin for a month without any problems, the GP will then transfer the prescription into the repeat file. Repeat medicines that have been discontinued are clearly marked with an ‘inactive’ flag on the practice computer system. All others are current and have not been cancelled by GPs and are labelled ‘active’.

At baseline (and follow up), researchers restricted their searches of patients’ records to the ‘active’ prescriptions in the repeat prescription file in order that we collected outcomes only for those patients who had statins prescribed as a repeat prescription that was active on the date of data collection. One month’s supply of statin was the minimum quantity which led to researchers identifying patients as receiving a prescription for a statin but only if this was recorded as ‘active’ and in the practice’s repeat prescribing file.

The frequency and timing of cholesterol testing differed between and within practices, and patients were called for tests at the discretion of the GP. Patients without any record of a cholesterol test were assumed to have their cholesterol not at target (this assumption is widely accepted by UK practices and is used in the GP General Medical Services Contract Quality and Outcomes Framework). ^3^

**References**

1. O’Brien MA, Rogers S, Jamtvedt G, Oxman AD et al. Educational outreach visits: effects on professional practice and health care outcomes. Cochrane Database of Systematic Reviews 2007, Issue 4. Art. No.: CD000409. DOI: 10.1002/14651858.CD000409.pub2.

2.Avorn J, Soumerai SB. Improving drug therapy decisions through educational outreach. A randomized controlled trial of academically based “detailing”. N Eng J Med 1983;308: 1457-1463

**3.** Department of Health: New GMS contract 2003. Investing in General practice. NHS Confederation and the British Medical Association. London 2003.
