# Supplementary material for: A Cluster Randomised Controlled Trial of a Pharmacist-Led Collaborative Intervention to Improve Statin Prescribing and Attainment of Cholesterol Targets in Primary Care
Source: PLoS One. 2014 Nov 18;9(11):e113370. doi: 10.1371/journal.pone.0113370 (PMC4236200; doi:10.1371/journal.pone.0113370)
Supplement: Protocol S1 — Study protocol. (DOC) [file pone.0113370.s003.doc]

**Study protocol File S1**

**A cluster randomised controlled trial of a pharmacist-led collaborative intervention to improve statin prescribing and attainment of cholesterol targets in primary care**

**Research Team**

Richard Lowrie,^1^ Alex McConnachie,^2^ Suzanne Lloyd, ^2^ Jill Morrison. ^3^

**Affiliations**

^1^NHS Greater Glasgow and Clyde, Scotland, UK; ^2^The Robertson Centre for Biostatistics,

University of Glasgow, Scotland, UK; ^3^General Practice and Primary Care, Centre for

Population and Health Sciences College of Medical, Veterinary and Life Sciences, University

of Glasgow, Scotland, UK

**Contents**

**1. Critical review of literature and rationale for SOS study**

**1.1 Overview and origins of prescribing support in Primary Care**……….......5

1.1.1 ‘Rational’ prescribing and support……….........................................................5

1.1.2 The prescribing knowledge – practice gap ………............................................5

**1.2 Prescribing Support at practice level: prescribing support pharmacists**..6

1.2.1 Prescribing Support models………..................................................................7

1.2.2 Passive dissemination of prescribing information………................................7

1.2.3 Prescribing audit, analysis and feedback………...............................................8

1.2.4 Prescribing formularies……….........................................................................10

**1.3 Educational outreach**……….........................................................................10

1.3.1 Limitations of current educational outreach models……….............................11

1.3.2 Theoretical principles underpinning prescribing support models……….........12

1.3.3 Information transfer………...............................................................................12

1.3.4 Patient perspectives………...............................................................................13

1.3.5 Underpinning the design of a new intervention with theoretical approaches...13

1.3.6 Tailoring interventions to overcome implementation barriers………………..15

**1.4 Features of academic detailing or educational outreach for prescribing**..17

1.4.1 Multifaceted approaches……….......................................................................18

1.4.2 Multiple therapeutic topics………....................................................................18

1.4.3 Number of visits………....................................................................................20

1.4.4 Overcoming organisational barriers to implementation………........................22

1.4.5 Educational outreach plus additional strategies……….....................................23

1.4.6 Cardiovascular disease focus……….................................................................25

1.4.7 Pharmacy led educational outreach………........................................................28

1.4.8 Pharmaceutical industry models………............................................................29

1.4.9 Targeted or untargeted educational outreach……….........................................30

**1.5 The need for better evidence to underpin prescribing support**……………31

**1.6 Therapeutic uses of statins in vascular disease**…………………………….32

1.6.1 The basis for clinical guidance on statins………..............................................32

1.6.2 The case for improving Statin prescribing……….............................................33

1.6.3 Could educational outreach help improve statin prescribing? ………..............34

**1.7 Template for a new intervention**……….......................................................34

**1.8 Template for a study to test a new intervention**………..............................35

**2. Methodological considerations arising from previous, related work**………..36

2.1 Clustering……….................................................................................................36

2.2 Pre-Randomisation and randomisation ………...................................................36

2.3 Recruitment and generalisability……….............................................................37

2.4 Choice and measurement of outcomes……….....................................................38

2.5 Economic appraisal………..................................................................................40

**3. Study methods**………..........................................................................................41

3.1 Setting - NHS Greater Glasgow and Clyde………..............................................42

3.2 Aim of study………............................................................................................43

3.3 Endpoints………................................................................................................43

3.4 Sample size and power………............................................................................43

3.5 Statistical analysis………...................................................................................43

3.6 Ethical approval and study registration………...................................................44

3.7 Funding………...................................................................................................44

3.8 Participating practices and patients………........................................................44

3.9 Randomisation………........................................................................................45

**4. The Statin Outreach Support intervention**……….............................................46

4.1.1 Overview………...............................................................................................46

4.1.2 Before the first meeting ………........................................................................46

4.1.3 Meeting one………...........................................................................................47

4.1.4 Between the first and second meetings……….................................................48

4.1.5 Meeting 2………..............................................................................................49

4.1.6 Between the second and third meetings………................................................50

4.1.7 Meeting 3………..............................................................................................50

4.2 Usual Care……….............................................................................................50

**5. Appendices** ………...............................................................................................51

Appendix I Heart Protection Study – Subgroups for 1^st^ vascular event…………52

Appendix II Ethical approval…………………………………………………….54

Appendix III Local Cholesterol guideline…………………………………………55

Appendix IV Clinical Facilitation (Statin Outreach Support): a summary for practices…………………………………………………………….56

**6. References**………................................................................................................57

**Abbreviations**

ACE Angiotensin Receptor Enzyme

CABG Coronary Artery Bypass Graft

CHD Coronary Heart Disease

CVD Cerebrovascular disease

G Group (practice)

GG&C Greater Glasgow and Clyde

GMS General Medical Services

GP(s) General Practitioner(s)

HPS Heart Protection Study

ICC Intra Class Correlation

LTC Long Term Condition

MI Myocardial Infarction

NHS National Health Service

PVD Peripheral Vascular Disease

QOF Quality and Outcomes Framework

SH Single Handed (practice)

SOS Statin Outreach Support

SSRI Selective Serotonin Receptor Inhibitor

TIA Transient ischaemic attack

UC Usual Care

**1. Critical review of literature and rationale for SOS study**

**1.1 Overview and origins of pharmacist-led GP prescribing support in Primary Care**

**1.1.1 ‘Rational’ prescribing and support**

The term ‘rational prescribing’ was introduced by Gilley in 1994. It acknowledges variations in prescribing practice but asserts that some prescribing decisions are more appropriate than others. Rational prescribing was seen as the “appropriate, safe, effective and economic” use of medicines (Parish 1973). One observer suggested the patient’s quality of life should also be considered when aiming to achieve appropriate prescribing (Barber 1995). Toa achieve rational prescribing, ‘Prescribing support’ was conceived as a professional support mechanism addressing one or more of the components of the prescribing process, aiming to promote high quality, cost effective medicines use (National Prescribing Centre and NHS Executive 1998; Audit Commission 1994).

As expected from a process involving many inputs there are large variations in the extent and nature of prescribed medicines within and between practices. For example, variations in statin prescribing are well recognised, with studies explaining up to 20% of the variation (Packam 1999; Majeed 2000; Gibson 2002; Bradshaw 1999; Ward 2007). Contributing factors include the prevalence of CHD, and patient level socioeconomic deprivation.

Prescribing support is indicated from a fiscal perspective. Factors contributing to a growth in drug expenditure include increases in spend on disease prophylaxis, high cost/new products for previously untreatable diseases, increased expectations from the public and media together with innovative marketing strategies from the pharmaceutical industry. All of these pressures lead to a greater need (or perceived need) for rational, cost effective prescribing.

Programmes of prescribing support are intended to increase the rational use of medicines. Rational use should lead to decreased costs and improvements in the quality of care. The best case scenario is when both of these issues are addressed simultaneously, but it is difficult to choose a prescribing topic and prescribing support method which does both. There are examples of prescribing support initiatives that improve quality of care but increase costs, (Kreling 1989) and cost reduction programmes that lead to poorer quality of care (Bloom 1985). One report found practice level pharmacist prescribing support to be cost effective, compared with no prescribing support, in the context of a controlled trial (Rodgers 1999). If prescribing support achieves stated aims it may provide a means of reducing the lag time between publication of clinical evidence and implementation of that evidence in practice (Burrel 1990; *Effectiveness Matters* 1998).

**1.1.2 The prescribing knowledge – practice gap**

In relation to health related interventions including prescribing, there is often a failure to implement research findings (*Effectiveness Matters* 1998). Many recognised barriers are entirely rational e.g. patients or GPs deciding not to implement on a case by case basis, because circumstances differ from the trials upon which the evidence is based. These departures from the evidence base are common and account for much of this gap between research and practice (David 2003; Mair 1996; Sudlow 1997; Mashru 1997). However there are many other barriers to implementation of evidence based medicine and an associated range of theories on how to improve dissemination and implementation of research findings (Grol 1997, Fraser 2003) which could be applied at the individual or health care system level (Smith 2003).

Discussions on the implementation of evidence based medicine have acknowledged factors influencing prescribing. These include GPs’ knowledge, professional experience, role perception of GPs, time pressures (patient and GP), patient expectations, patient demand and the number of GPs in a practice. An understanding of these has illuminated the processes shaping GP prescribing decisions (Carthy 2000; Watkins 2003; Webb 1994; Britten 1997, Cockburn 1997). According to Haynes (2002), clinical decisions should consider evidence based medicine at the individual patient level. They describe a four part model:

1. Ascertainment of what is wrong with the patient and what treatment options are available;

2. Consideration of options informed by research evidence concerning the efficacy, effectiveness and efficiency of medicines when this is available;

3. Consideration of the patient’s preferences;

4. Application of clinical expertise to bring these considerations together and communicate the decision with the patient.

Anticipating specific barriers to implementation of the research evidence on a particular topic can inform solutions. Barriers are likely to vary depending on the practice, GP and patient’s circumstances. Finding out what the barriers are, before devising well intentioned solutions may be the best way forward (Haynes 1998). There are many reasons why the evidence based model of prescribing is not followed. Failure to take the patient’s beliefs and preferences into account during a consultation might reduce the likelihood of agreement on an evidence based choice. Known difficulties in the process of effective communication of the benefits and risks of any particular treatment cannot be underestimated (Veldhuis 1998; McColl 1998; Freeman 2001; Sweeney 1998).

**1.2 Prescribing Support at practice level: prescribing support pharmacists**

Recognising the potential for improvements in patient care and prescribing efficiency through direct support to GPs, additional investment was made to deliver complementary prescribing support at GP, patient and practice level. This move aimed to bring about effective prescribing change together with cost savings, in addition to encouraging GPs to prescribe in line with the evidence. Better collaborative working between medical and pharmaceutical professionals was encouraged and Prescribing Support pharmacists were introduced into general practices. Methods included working in practices, with full access to patients’ medical and prescribing information at practice level, to ensure prescribing advice is tailored to practices’ and patients’ needs. This also helped translate prescribing decisions based on guideline or formulary advice into action at patient level.

There were some calls for more practice based pharmacists, because anecdotal evidence suggested they were able to reduce prescribing costs (Wells 1997; Wells 1998; Department of Health and NHS Institute for innovation and improvement 2007). Others have asserted that there are many ways to reduce prescribing costs without involving prescribing support pharmacists (Tant 1999).

The methods adopted by General practice based pharmacists were diverse. So too are the terms used to describe the role. These have included “GP pharmacist” (Kempner 1996), “primary care pharmacist” (Marinker 1994) “clinical pharmacy service” (*Anon* 1996) and “consultant or freelance pharmacist” (Powell 1997). By default, the role involves a pharmacist working with medical practitioners and others in the practice team. The core objective is to identify, with the practice, areas of prescribing in need of attention or support. The pharmacist gains agreement on how best to support the area of prescribing and bring about change. Methods include modification of prescribing mediated through presentations and patient or practice level reports, prescribing or clinical audits. All approaches aim to influence GPs, nurses or patients directly. Success is measured by cost saving, better adherence to clinical guidance or both. Being closer to the prescribing process also means that evaluation of impact was immediate: within 1-2 months of introducing or encouraging a prescribing change, a shift in prescribing could be detected through a search of the practice’s repeat prescribing on the practice computer system. This acted as a useful reinforcement to support subsequent prescribing change.

**1.2.1 Prescribing Support models**

The introduction of general practice based pharmacy prescribing support started during the GP fundholding era, when ‘early adopter’ practices contracted work on a sessional basis from freelance pharmacists or from NHS contractor pharmacists with a special interest in the clinical use of drugs in Primary Care. As Pharmacy led prescribing support models have evolved over the past 15 years, general descriptions of their characteristics, costs and benefits have appeared (National Prescribing Centre and NHS Executive 1998; Kempner 1996; Powell 1997). Overall, the quality of evaluation varied and as is often the case with innovations in health care delivery, questions of reproducibility, effectiveness and value for money remained unanswered (Soumerai 1990; Soumerai 1989; Haaijer-Ruskamp 1995).

There are few scientifically rigorous studies of the effectiveness of prescribing support models other than medication review, in Primary Care. They appear as a subset of the larger body of research involving educational and other interventions that aim to change health professionals’ behaviour (O’Brien 2002; Oxman 1995; Davis 1995). One review of evidence on the impact of pharmacists providing a prescription review and monitoring service in primary care suggested improvement in patients’ clinical status and suggested reduced prescribing costs, while acknowledging the need for further research to confirm these findings (Tully 2000). The NHS Centre for Reviews and Dissemination subsequently disagreed with the authors’ suggestions of the likely effectiveness of pharmacists’ prescription review and monitoring activities, but agreed on the need for future research in this area. Further, the NHS centre recommended more independent and blinded outcome assessment, use of comparison groups and appropriate statistical analysis in future research (NHS Centre for Reviews and Dissemination 2004).

Approaches to prescribing support have evolved in response to the changing needs of patients, GPs, the evidence base for management of targeted conditions and financial constraints. Service evaluations have shed some light on the apparent effectiveness of different models of prescribing support delivered by primary care pharmacists (Wilson 1997; Jesson 1997; National Prescribing Centre and NHS Executive 1998; Squires 1997; National Health Service Executive 1996). However, due to a lack of adequate description of roles and methodological rigour in evaluation, much of the available published work has not clarified which models of support are effective and efficient. Inadequate design, lack of a control group, poorly defined interventions and an emphasis on process related outcomes limit the generalisability of findings and have led to a call for more rigorous comparative effectiveness research in this area (Beney 2000).

**1.2.2 Passive dissemination of prescribing information**

Unsolicited, passive dissemination of printed educational material is characterised by a lack of targeting of the message or assessment of the educational needs of the recipient. The approach is thought to be relatively ineffective as a means of changing healthcare professionals’ behaviour according to a Cochrane Review on the subject (Freemantle 2005). While there were only 11 studies fitting the criteria for inclusion in the review, evaluation of their combined impact was impractical because of the poor reporting of results and inappropriate primary analyses.

Much of the research in this area relates to the dissemination of prescribing guidelines, or clinical guidelines (‘systematically developed statements to assist practitioner decisions about appropriate healthcare for specific clinical circumstances’) (Institute of Medicine 1990).

Passive dissemination of prescribing guidelines may act to raise awareness which may help pave the way for change at an individual level (Soumerai 1990; Denig 1990; Anon *Implementing clinical guidelines: can guidelines be used to improve clinical practice?* 1994). However, some individual studies indicate that there is no significant change in GP’s attitudes and knowledge following direct mailing of consensus statements (Hunskaar 1996) or guiding prescribing decisions e.g. prescribing of antibiotics for acute conditions (Schaffner 1983). Davis reviewed the literature on different forms of prescribing support and concluded that commonly used methods of delivering education, such as conferences, have only limited impact on improving professional practice (Davis 1995).

Publishing and distributing national guidelines on cholesterol management (focussing on the use of statins and dietary measures), does not appear to have contributed to changing doctors’ approaches to management (Sempos 1993). However one report suggested that a simple, passive educational intervention can change prescribing behaviour, compared with feedback and education (Schectman 1995). In addition, it is thought that the publication of major trials heralding a significant shift in prescribing trends may lead to changes in prescribing (Muhammad 2001). Muhammad investigated whether the publication of three landmark statin trials were associated with an increase in the market share of statins in Canada and found a study specific effect, post trial publication. However, the strength of this evidence was weak: their study lacked a control group and did not account for confounding.

It stands to reason that strategies most likely to change practice are those which are actively communicated, underpinned and shaped by behavioural change and adult learning theory (Foy 2001).

It should be remembered that conferences, presentations and distribution of clinical guidelines may not aim to change professional practice. Instead, they may aim only to increase awareness of a particular topic, which in turn may create a better starting point for introduction of more active dissemination strategies to facilitate change, at some point in the future. In fact, there is some suggestion that the distribution of bulletins through post or email is a low cost approach and any small effects may be worthwhile in the long run (Soumerai 1986). One study in North America showed passive dissemination, in addition to educational outreach, improved marginal cost effectiveness (Soumerai 1986).

Another prescribing support model combining passive dissemination with more tailored approaches is the use of postal prompts to GPs, for named patients. In a London based study, Feder et al trialled this approach, to improve cholesterol measurement together with the prescribing of Beta Blockers and statins, for patients who had recently been discharged from hospital after a coronary event. Their intervention increased the proportion of patients attending for cholesterol checks at their practice but failed to improve prescribing (Feder 1999).

**1.2.3 Prescribing audit, analysis and feedback**

In general, published audit work in healthcare settings has focussed on standards of clinical practice or diagnostic performance rather than prescribing (Mugford 1991; Buntix 1993).

Prescribing audit is the systematic, critical analysis of the quality of prescribing. It does not make inferences about the quality of life for the patient, but it can be used to estimate the population level impact of evidence based prescribing choices. For example, prescribing of angiotensin converting enzyme inhibitors (known to be effective and cost effective for heart failure due to left ventricular systolic dysfunction) may vary significantly across different practices. If the practices have similar demographics and access to effective services, then excluding any other justifiable reasons for the difference, prescribing audit enables recognition of what is feasible and a starting point for improvement.

The Cochrane Collaboration assessed the effects of audit and feedback on healthcare professionals’ prescribing and patient outcomes. They found it to be effective, but only moderately so. One interacting factor consistently shown to predict the effectiveness of prescribing audit across studies was baseline non-compliance with recommended prescribing (absolute effects likely to be greater) (Jamtvelt 2005). This might be expected, because there may be more scope for change if the starting point is lower.

Practice level summary and analysis of prescribing data has been used to compare the prescribing of medicines between general practices, but the impact requires to be evaluated beyond the short term and include a comparator group (Harris 1993). Prescribing audit creates an opportunity for discussion and feedback on the reasons for prescribing variance and departures from ‘best’ practice. One study showed it to be effective and more efficient than educational outreach (Anderson 1996).

Prescribing audit is a useful means of differentiating between established and best practice. In some cases, raising awareness of these differences during a face to face meeting may be sufficient to trigger a change in prescribing, but evidence is lacking for this effect. Perhaps the best approach is to combine it with several other models, again, to reinforce the communication of a message, in much the same way that multidisciplinary teams work together to achieve a more pronounced effect than uni-professional, solo efforts. One evaluation of prescribing audit delivered by community pharmacists (who worked on a sessional basis within General practices) showed cost savings and reported improved levels of communication and co-operation between GPs and community pharmacists. GPs also felt that the quality of the meetings was superior to other local postgraduate events (Pilling 1998).

‘Prescribing indicators’ are now in common use across the UK. These are derived from prescribing audit and are set thresholds of prescribing of targeted medicines e.g. the least expensive bisphosphonate from the available options sharing the same indication. While opinions vary as to the suitability of some indicators (Asworth 2002), if a practice achieves their indicator threshold, a payment is triggered. This incentive to the practice is smaller than the savings generated as a result of the change in use the drug under question. While there is very little empirical evidence of the impact of such schemes on quality improvement, they are known to change prescribing, in the same way as evidence based prescribing for targeted LTCs has improved as a result of financial incentivisation through the new GMS contract (Roland 2004).

Overall, there are few scientific assessments of the effectiveness of prescribing audit, and significant variation in the nature, intensity and delivery mechanisms, thus limiting transferability of the model as a tool for prescribing support. However, there may be merit in combining this with other approaches. As an example of this, Eccles showed that routine attachment of educational reminder messages by reporting software or coloured stickers (in addition to audit and feedback) was effective in changing radiology referrals (Freemantle 2001). This study highlighted one of the difficulties in evaluating studies of audit and feedback: describing the intervention and context sufficiently to enable appraisal.

**1.2.4 Prescribing formularies**

Formularies are a limited range of medicines for specified conditions. The medicines are chosen on the basis of clinical evidence of relative efficacy and relative cost. The main reasons for using formularies are to promote rational prescribing and limit costs (Jolles 1981; Reilly 1989; Greenfield 1982; Harding 1985; McGavock 1990) Formularies are designed to encourage the uptake of more effective prescribing (clinically and economically). They are also seen as a mechanism for quality improvement in general practice (Roland 1998).

A systematic review concluded that there is insufficient robust evidence to justify the use of formularies per se, or involvement in their development, as an effective model of modifying prescribing behaviour (Pearson 2003). Methodological weaknesses limited the usefulness of the authors’ conclusions. One report (only available as a supplement) described the use of “closed formularies” (a tighter list of options restricting choice to a bare minimum with non formulary prescribing disabled through computerised restrictions) combined with several other approaches e.g. academic detailing, and reported effectiveness (Sbarbaro 2001).

**1.3 Educational Outreach**

**Overview**

Educational Outreach can change health professionals’ prescribing behaviour (New 2004;

Boardman 1999; De Wilde 2003; Davis 2005). It involves a face to face meeting between a person knowledgeable in therapeutics and a healthcare professional, in the healthcare professional’s own setting. It commonly incorporates the provision of feedback on prescribing issues and the following key features and techniques are thought to maximise the chances of educational messages being understood and the learning being implemented:

- Focussing on a targeted, small group of clinicians;
- Defining clear educational and behavioural objectives;
- Establishing credibility;
- Stimulating active participation;
- Using concise graphic educational material;
- Highlighting and repeating essential messages;
- Providing positive reinforcement in follow up visits.

(Avorn 1983; van Eijk 2001; Soumerai 1990; O’Brien 2002).

The term ‘Detailing’ was originally coined to describe a form of educational outreach pioneered by the Pharmaceutical Industry through representatives promoting their products. Marketing and selling strategies are incorporated, tailored to the doctor’s personality, practice style and preferences. Acknowledging the usefulness of this approach in a commercial, for-profit sense, the methods have been applied to benefit the ‘not for profit’ sectors e.g. the National Health Service. This led to the terms ‘Public interest’ or ‘academic’ detailing, and the process of non commercial, short, face to face, in-service interactive education by a trusted outsider trying to promote evidence based choices among physicians. The median improvement in prescribing from this approach is estimated to be approximately 6% with a range from -4% to 17%, up to 1 year (Grimshaw 2004) although Avorn achieved a difference of 18% (Avorn 1983: see Table 1). Core features of this complex model are not well understood. There is no consensus or guidance on how narrow or wide the topic should be, or the relative efficacy of components e.g. written compared with face to face feedback. Frequency of contacts between pharmacist and prescriber vary between studies. Some suggest that eight, one hour sessions per practice are sufficient to effect prescribing change at practice level (Squires 1997), while others recommend meetings every 4 – 8 weeks, of one hour duration (Pilling 1998), but there is no consensus, partly because of the differences in context, content and delivery, topics, training of staff involved.

**1.3.1 Limitations of current educational outreach models**

Compared with conventional group based educational meetings, detailing is thought to be more effective (Bernal-Delgado 2002; O’Brien 2002). Educational meetings generally focus on a wider subject, take place outwith the provider’s practice and involve groups of professionals together. To date, the published reports of educational outreach describe interventions focussing on educational exchange, without any practical, ‘hands on’ assistance to introduce changes to practice, or without much thought about how changed practice can be sustained (O’Brien 2002). The messages conveyed during educational meetings are not usually individualised to each GP’s needs, unlike the approach taken by pharmaceutical industry representatives, who make every attempt to tailor their messages to meet GPs’ real or perceived needs. Marketing and behavioural or personality profiling are used to achieve this end.

The level of interactivity in studies of detailing is difficult to ascertain from available evidence. Delivery of outreach appears to involve prioritisation of the message (usually abbreviated clinical guidance) over the process by which the message is communicated. This runs the risk of failing to acknowledge the GP’s circumstances, baseline prescribing habits, beliefs, opinions and not least, patient preferences. In 2000, the Medical Research Council acknowledged the importance of basing implementation on a scientific understanding of the behaviours that may need to change, the relevant decision making processes and the barriers and facilitators of change (Medical Research Council 2000). In view of this guidance, and the increasing reliance on prescribing as a means of tackling or preventing ill health, it is timely to introduce a new form of detailing, which supports implementation by tailoring the messages to each GP, and providing practical help in addition to educational outreach.

**1.3.2 Theoretical principles underpinning prescribing support models**

Theory building and testing are important considerations for developing new approaches to prescribing improvement. In 1990, Raisch produced a useful model of methods to influence prescribing (Raisch 1990a, 1990b). He drew on four bodies of literature to develop his theoretical model and suggested that the greatest chance of success comes from constructing an intervention which incorporates:

- Theoretical prescribing models;
- Theories of persuasion;
- Research articles of programmes to improve prescribing;
- Theories of human inference.

The prescribing habits of individuals were acknowledged to be relatively stable over time. Changes usually occur slowly and as a result of various influences including scientific papers, specialist recommendations, postgraduate educational or practice meetings, feedback from patients and the pharmaceutical industry. Lack of knowledge is only one reason for suboptimal prescribing and may not be the factor limiting change in many cases. Raisch also suggested that of the numerous factors influencing prescribing decisions, it is likely that some are rational and others irrational. Accepting this to be true, and assuming the general aim is to change prescribing decisions in line with standards set out in clinical / prescribing guidelines, it follows that rational influences are more easily identified and resolved than irrational influences. Depending on the relative weight of rational and irrational influences, this is a limitation to any prescribing support model and should be borne in mind. Ignorance, incompetence, poor management, and sometimes a deliberate disregard of established knowledge may get in the way of best practice when it comes to implementation of guidelines in general (Clinical Resource and Audit Group 1993). This approach may also be seen as displaying healthy scepticism because guidelines are not always based on impartial, best evidence (Delamothe 1999; Grol 1997). Not all patients fit a guideline algorithm for each of their conditions. Doctors may be exercising a conservative approach to minimising the risk of exposing patients to treatments that (in some cases) have not yet gained sufficient exposure in a real world population. Practitioners may regard guidelines as documents that focus on scientific knowledge rather than the needs of their end users (Fairhurst 1998). There are many examples in general practice of so called “clinical evidence” being of questionable quality, relevance, objectivity or simply out of date by the time it arrives in practices. As one GP put it: “the fickleness of evidence is inconvenient but would be easier to live with if it was more widely acknowledged in discussions of implementation” (Temple 2002).

From the available evidence, a novel intervention should include features which enable identification or anticipation of individual GPs’ reasons for not implementing the evidence.

If a guideline has features that are conducive to increasing uptake, this is likely to improve implementation potential. Several features are known to be useful, including congruence with existing prescribing norms, precise descriptions of actions required and the need for the message to be compatible with GPs’ values (Grol 1997; Grol 1998; Burgers 2003). Involvement of GPs in the process of guideline development may be consistently effective as a means of improving prescribing (Pearson 2003).

**1.3.3 Information transfer**

Theories of information transfer are relevant to prescribing change interventions because facts are learned more easily if presented in ways that enhance retention. It is therefore important to link new prescribing information to what the prescriber already knows. This creates the need to find out what the prescriber knows in advance – a marketing technique used extensively by the pharmaceutical industry. If prescribing information is presented in context e.g. at the time of an eligible patient’s appointment, timely retrieval is more likely. Active discussion e.g. using therapeutic dilemmas or clinical scenarios from practice may also help ensure detailing messages are absorbed, retained and used in practice.

There is a recognised gulf between what is known about changing prescribing behaviour and what is practised by those aiming to change it. This has led to a range of theories describing how to improve dissemination and implementation. These can be applied at the individual or health care system level (Smith 2000; Prochaska 1984; Bero 1998).

Some theories are intuitive e.g. Fraser argued that as evidence is translated into practice by the pushing out of ideas by the spreaders of best evidence, change was more likely to take place if the focus is on the prescriber (who they describe as the ‘adopter’) (Fraser 2003). It stands to reason that adult learning and persuasive communication theories could be put to good use in the move to improve prescribing. If the theories are applied by the person instigating the change in prescribing (in this case, the pharmacist), they suggest the importance of using repetition and reinforcement, recommending alternative prescribing strategies, supporting recommendations by credible third parties e.g. expert clinicians. Offering evidence based, unbiased advice and stimulating interactive discussion are ways of establishing and sustaining engagement compared to other strategies e.g. passive dissemination of sponsored promotional material. Other features likely to increase the ability to improve information transfer and the uptake of better prescribing include features relating to the pharmacist delivering the information e.g. credibility, expertise in the relevant topic area (Lipton 1995).

Freeman demonstrated this point by showing that evidence based medicine can be viewed as a language to describe formalised explicit knowledge (Freeman 2001). In contrast, tacit knowledge includes intuition and problem solving ability that is gained through experience and interaction with people. It is important in decision making and is not often made explicit or externalised. In constructing a new educational outreach-type model, it is important to create an environment for the opportunities to arise when pharmacist-detailers find out GPs’ tacit knowledge about the detailing topic. One way to do this could be to arrange a face to face meeting to explore tacit knowledge. Another way to find this out is to carefully gather information from past prescribing decisions.

**1.3.4 Patient perspectives**

A frequently overlooked consideration in prescribing change models is the importance of patients’ beliefs and choices. Patient related barriers e.g. their beliefs and preferences ought to be acknowledged and addressed (Lipton 1995). In reviewing educational interventions targeted directly at patients, Mullen identified several principles which, if addressed, rendered the approach more effective (Mullen 1985). While Mullen’s principles do not apply directly to the model of pharmacists detailing with GPs, they are congruent with many of the techniques used as part of the academic detailing approach. These include provision of advice that is relevant to the receiver’s circumstances, involvement of the receiver in the design of the intervention, reinforcing desirable changed behaviours, anticipating and addressing practical barriers to changing behaviour and the importance of delivering educational messages through multiple media e.g. written and verbal. A scoring system based on these principles has since been used to evaluate the quality of inter-professional educational interventions, confirming the potential suitability of Mullen’s work in this context (Cooper 2001).

**1.3.5 Underpinning the design of a new intervention with theoretical approaches**

It is recommended that the design of interventions aiming to change health care professionals’ practice is underpinned by theoretical principles of behavioural change including adult learning theory (Foy 2001; Medical Research Council 2000, 2011). An understanding of theories of behavioural change and acknowledgement of factors known to influence prescribing practice is therefore a useful starting point to designing a novel intervention to change prescribing (Foy 2001). As with complex interventions in general, this is better understood in the context of other relevant intervening variables. Literature in this area is diverse and it is often difficult to extract practical lessons that could be transferred into a new prescribing change model. However a useful, pragmatic description of a range of factors recognised (by doctors) as important in changing clinical practice is given by Allery (1997). Fifty GPs and 50 consultants were interviewed and using a critical incident technique, they ascertained categories of reasons influencing their clinical practice. While the scientific validity of their findings was not helped by the absence of a comparator group, (they did not ask a comparable group of GPs who did not make changes), it was clear that help with organisational factors combined with education, and contact with an independent professional e.g. a pharmacist, was regarded as a positive influence. They recommended that a wide range of factors need to be considered in the provision and evaluation of educational activities, because of the wide range of factors involved in changing practice.

Schwartz explored 114 doctors’ motivations for apparently inappropriate prescribing decisions within a larger randomised controlled trial (Schwartz 1989). Of the 110 responses elicited, the most common reason for prescribing medicines known to be of limited usefulness was patient demand. Most prescribing decisions seek to balance perceived efficacy with perceived risk of adverse effects. The authors recommended that greater attention should be paid to attitudes and motivations concerning suboptimal prescribing if ‘detailing’ programmes are to be successful. This observation has implications for the design of a study testing a novel outreach model: targeting prescribers known to prescribe sub-optimally (in comparison with clinical guidelines) is more likely to generate positive results compared with an untargeted approach. However, in the National Health Service, the level of equity is important, and in primary care general practices, all patients who are eligible for a statin or eligible for an optimal dose (i.e. those not prescribed a statin or those prescribed one, but at a dose less than guideline recommendations) ought to be offered one. These patients exist in every practice therefore targeting of all practices may be necessary.

In 1996, Armstrong interviewed a purposeful sample of 18 GPs from London and hypothesised that there were three models of prescribing change (Armstrong 1996). Notes taken during semi-structured interviews were analysed and identified a challenge model, a continuity model and an accumulation model. An important practical conclusion from this work was the unanimous view of the participating GPs that the initial change in prescribing was precarious and needed reinforcement. This finding coincides with much of the literature describing effective educational outreach programmes, where repeated visits serve the purpose of reinforcing desired change (Oxman 1995). A similar observation was noted from effective programmes of changing patients’ health behaviours including adherence to medicines (Mullen 1985). Reinforcement and support for the initial steps towards prescribing change therefore appear worth integrating into a novel educational outreach model. Cognisance should also be given to doctors’ overriding concern when considering whether to prescribe: preservation of the doctor-patient relationship. Non clinical factors have an important influence on prescribing e.g. attitudes, perceptions and experiences of the GP (Bradley 1992). All of these factors should be acknowledged before introducing an educational programme.

Prescribing support interventions should be designed in a way that enables the providers to recognise the barriers known to limit the translation of evidence into practice. Different adopters are likely to present different (often personal and wholly legitimate) reasons for not following the evidence based model of prescribing (Veldhuis 1998; McColl 1998; Freeman 2001; Sweeney 1998). Organisational barriers are a recurrent theme. The EBOR study investigators were unsuccessful in their attempt to change clinical practice following outreach visits, citing organisational difficulties as one of the main barriers to implementation (Freemantle 1999). Different barriers are likely to arise in different practices, and between individuals in the same practice, so a commonsense approach would suggest separate strategies are needed to overcome these. In Glasgow, we have found that different approaches are needed to engage different patient subgroups. For example, inviting Urdu or Punjabi speaking South Asians in their native language through a phone call is more successful than using English. We have found that a pro-active, empathic approach, tailoring appointments to the individual’s availability is more likely to lead to attendance in patient groups usually perceived as ‘hard to reach’ in areas of socioeconomic deprivation (Lowrie 2010b). Communicating using methods used routinely by the individual e.g. appropriate language or texting on a mobile phone, is more likely to bring about success than a printed letter, particularly if levels of literacy are low (O’Donnell 2009; Fisk 2006). In their critical review of studies of educational programmes designed to improve prescribing in North America, Figueiras concluded that the more personalised the intervention to the prescribers’ needs, the more effective the strategy is likely to be (Figueiras 2001). The authors substantiated this by conducting their own pragmatic controlled trial evaluating the effectiveness of two multifaceted educational strategies aimed at improving prescribing standards in primary care. Comparing one to one education with group sessions, focussing on reduced prescribing of non-steroidal anti-inflammatory drugs, they showed that single outreach visits of one to one sessions were more effective than group sessions.

In 2004, Schumock asked a sample of 150 doctors, pharmacists and formulary committee members to express their opinions as to the importance of factors influencing prescribing decisions. They found significant differences between professional groups. Doctors rated the recommendations of pharmacists, formulary committee members, prescribing guidelines and cost comparisons of lower influence than the pharmacists themselves (Schumock 2004). If generalisable, this result highlights the importance of asking prescribers to identify their biggest prescribing influences, and seeking the views of whoever the prescriber identifies as being educationally influential.

GPs’ perspectives of the utility of detailing information are sparse, but McColl produced a useful précis. He asked a sample of 302 GPs from Wessex, UK, what they rated most highly as helping clinical decision making. Using a semi structured questionnaire, they reported that GPs wanted evidence presented in short, understandable packets, with recognisable quality standards, using understandable descriptions e.g. NNTs of useful outcomes (McColl 1998). None of their requirements are too challenging, even in the current resource restricted NHS environment.

**1.3.6 Tailoring interventions to overcome implementation barriers**

From Grol’s summary of factors limiting or restricting behavioural change, it is possible to identify barriers which may need to be overcome before implementing clinical guidelines:

- Orientation: becoming informed about the existence of new guidelines; feeling interest and commitment;
- Insight: understanding the guidelines, awareness of gaps in own performance and the need to change;
- Acceptance: positive attitude to the new guidelines accompanied by intention to change;
- Change: implementation in practice, experimentation, recognition of positive outcomes and maintenance of change (Grol 1992).

Differences between guideline recommendations and prescribing in practice are inevitable when guidelines are drawn from discrete studies in populations and settings that may be far removed from usual care. Many other factors contribute e.g. the organisational context of the practice or the nature of the clinical or prescribing topic compared with the priority assigned to it in the practices under scrutiny. It is therefore unreasonable to aspire to 100% guideline adherence in any given topic and it seems likely that tailoring interventions to address these issues in a GP by GP and Practice by Practice basis will have most chance of overcoming implementation barriers. Bero concurred with this view, suggesting that the methods of implementing evidence based prescribing should be guided by evidence on their effectiveness, the nature of the change being suggested and a prior assessment of the obstacles to change (Bero 1998).

Fretheim constructed a tailored intervention incorporating an educational outreach visit with audit and feedback and computerised practice based reminders (Fretheim 2003). Pop up reminders appeared on physicians’ computer system in response to a consultation with a patient who had high blood pressure or high cholesterol. This enabled rapid calculation of cardiovascular risk and printed educational material for the patient; both factors had been previously identified as rate limiting.

Psychological methods have been used to guide GPs in the implementation of guidelines for depression in primary care (Baker 2001). Baker used content analysis of interviews with GPs about their performance. This enabled assessment of the likely obstacles to guideline-led depression management and pinpointed a psychological theory that best explained the relevant obstacle. Implementation methods were constructed in response to these theories and obstacles, and GPs were encouraged to adopt the recommended implementation methods. Important learning from this trial included the importance of the individualised discussion with each GP as a means of identifying reasons why the guideline evidence was not implemented, followed by tailoring of interventions to support the GP to adopt evidence based practice. The researchers did not seek to identify organisational or other obstacles at the practice level because it was not possible to change them.

In some cases, organisational inertia may be the rate limiting step and needs to be considered then tackled. For example, a new trial investigating statin prescribing for a cohort previously untreated would first require identification of those eligible to receive it within each practice. Following this, an invitation to attend an appointment, and offer of a statin would be required. Each step takes time and a systematic approach; adequate and persistent call and recall underpinned by accurate and complete practice based disease registers to maximise uptake and minimise dropout, particularly in areas where health literacy is poor and engagement with preventative or screening appointments is low.

Fretheim attempted to implement an educational intervention aiming to improve prescribing of antihypertensive and cholesterol lowering medicines. In their analysis, they used structured reflection and focus groups to identify barriers to uptake of their educational messages (Fretheim 2004). Their findings were comparable to that of other teams: gaining commitment to change is predictive of actual change, which is a useful approach, easily replicated (Wakefield 2003), and worthwhile incorporating into a novel detailing approach.

In the international Drug Education Project, a new educational programme facilitated by GPs or pharmacists for peer groups of doctors to improve the treatment of asthma patients in the Netherlands, Norway, Slovakia and Sweden, was developed and tested (Veninga 1999). The multicentre trial design was parallel, randomised and controlled to test the effect of an educational programme on competence and prescribing. The intervention comprised individualised feedback on the underlying reasons for prescribing choices. Feedback was presented and discussed in small peer groups. Significant improvements were noted in all competence and prescribing outcomes in all countries except for Sweden, where improvements were made on all outcomes but the effect failed to reach statistical significance. Control group practices received prescribing guidelines only. The authors comment that the tailored approach was the key to effecting change. This result is striking because of the consistently positive effect despite the between, and within, country variation in continuing medical education programmes, organisational barriers to change, different baseline prescribing behaviours and attitudes of GPs. In reviewing this paper, it becomes apparent that a tailored intervention (if carried out effectively) may be more likely to work across different healthcare settings because it identifies and seeks to address the expressed educational and organisational needs of each individual, whatever the individual’s experience, background or practice organisation.

Another group of researchers tailored their outreach visits to the needs of each practice and found that this feature predicted effectiveness (Hulscher 1998). However, the extent to which they enabled the doctor to shape the educational exchange led to wide variation in the intensity and duration of their input. In their discussion, they called for standardisation of visits and standardisation of the skill-set of those delivering them, from an economic perspective.

Most GPs do not work independently. Instead, they work as part of a practice team and therefore, educational interventions aiming to change prescribing need to be seen in the practice context. There will be organisational barriers and difficulties at practice level that need to be identified and overcome. For example, implementation of the evidence base around offering statins to all patients with established vascular disease will require careful verification of patients most likely to benefit. In addition, it cannot be assumed that acquisition of knowledge about the ‘best’ treatment strategy is sufficient to overcome change: the rate limiting steps may be the systematic call and recall, chasing of non attendees, not to mention the need to overcome prescribing inertia (Nazareth 2002).

Anticipation of barriers to implementing change appears to be the exception rather than the rule, in previous research. In keeping with the majority of educational outreach trials, Doyne constructed educational modules without involving the end users. They did not seek to discover barriers to implementation in their randomised controlled trial involving an intervention to change antibiotic prescribing for acute conditions. Their control group received summary information by post only. Their intervention characteristics were poorly described; it is likely that they presented information in a didactic fashion, in small groups, without much discussion or interaction. Their ‘academic detailing’ model failed to achieve a statistically significant effect when the impact was measured by the under, or over, use of amoxicillin or cephalosporin antibiotics respectively (Doyne 2004).

The EBOR trialists were unsuccessful in their attempt to change clinical practice following tailored outreach visits. In their discussion, they cite organisational difficulties as one of the main barriers to implementation, rather than a mismatch between the intervention and the GPs’ information needs (Nazareth 2002). In particular, they lamented that they did not anticipate the level of organisational difficulties they encountered e.g. setting up a disease register and the call/recall problems in practices.

The Cochrane database of systematic reviews assessed studies of tailored interventions to overcome identified barriers to health professionals’ performance (Shaw 2005). Inclusion criteria were typically selective: randomised controlled trials that reported objectively measured professional practice in which at least one group received an intervention designed to address prospectively identified barriers to change. While one analysis of their findings found tailored interventions to be more effective (Odds Ratio 2.18 (95% CI 1.09 – 4.34; p = 0.026), another analysis using a different criteria gave a comparable odds ratio, but failed to reach statistical significance. Overall, they concluded there was insufficient evidence to confirm interventions tailored prospectively to identify barriers actually improve care and patient outcomes. However, sufficient funding and time are not always available to deliver and evaluate complex interventions in primary care. Further, complex interventions have, by definition, several interacting components, making it difficult to pinpoint the key feature.

For general practice level interventions in the United Kingdom, it appears that involving participant GPs in the design of their own intervention could be important (Langham 2002).

**1.4 Features of academic detailing or educational outreach for prescribing**

In the original report of academic detailing, Soumerai described a comprehensive list of features of the intervention (Soumerai 1987). Brevity, repetition and reinforcement of desired prescribing practices were cited as key factors contributing to the success of their educational messages (Soumerai 1990). The same authors had previously concluded, from a randomised controlled trial involving 435 doctors, that face to face educational sessions are effective in improving prescribing practices. Their sample of doctors included different age groups, speciality, urban/rural setting or extent of prior prescribing of the target drug groups.

In another report evaluating the effectiveness of academic detailing as a means of improving aspects of health professionals’ practices (focusing on mental health care), Soumerai stressed the importance of person to person contact with credible experts and the provision of information for GPs to try structured alternatives to help facilitate change. Four trials which had tested detailing as a means of improving mental health care were reviewed. Detailing was found to be effective as a means of reducing the overuse of unnecessary medicines, however, improvements in markers of mental health or the detection of mental health problems remained unchanged (Soumerai 1998).

Since then, many different permutations of the detailing model have been delivered and tested. The following appear to be important, and will be carried forward into the new model described and tested in this study.

**1.4.1 Multifaceted approaches**

Multifaceted interventions have tended to anticipate, facilitate and reinforce desired behaviours. They appear more likely to succeed compared with single strategies or single interventions (*Getting evidence into practice* 1998; Wensing 1998; Grol 1992). It follows that approaches to changing prescribing that use a greater number of evidence based strands may be more effective than those using less. Madridejos-Mora used a combination of educational recommendations and feedback of individualised prescribing, to improve prescribing quality (Madridejos-Mora 2004). Quality in this context was defined as improvements in the uptake of evidence based recommendations for prescribing of non-steroidal anti-inflammatories, ulcer healing drugs and antibiotics. Improvements in the quality of prescribing, particularly reducing overprescribing, was noted in practices exposed to their combination approach compared with a single approach. However, their description of the intervention is short on detail, limiting further critical appraisal.

Few trials other than that conducted by Madridejos-Mora have attempted to combine and compare more than two different components. None have accessed practice held patient level information and used that to identify patients who are eligible for change, then supported GPs to contact all eligible patients systematically.

Attempting to influence patient behaviour is rare; only one account of academic detailing reported the use of posters in waiting areas together with patient level education. When the dual approach of GP education and patient information were combined, the intervention proved successful and the authors cited the combination as one of the key reasons for effectiveness (Harris 2003).

While multifaceted approaches may be more likely to generate positive outcomes, simple approaches should not be overlooked because they may be easier, quicker and cheaper to deliver and evaluate. For example, Hux conducted a randomised controlled trial of antibiotic education and confidential feedback to GPs in Canada. They hypothesised that a decrease in the prescribing of inappropriate antibiotics and an increase in first line antibiotics would result. In achieving their primary outcome, they concluded that the simplicity of their programme, particularly the confidential aspect of their feedback, was vital in achieving desired outcomes (Hux 1999).

**1.4.2 Multiple therapeutic topics**

Some researchers, like Madridejos-Mora, tried to effect change in several therapeutic areas within the same educational outreach programme. Assuming the detailing approach is effective, it might appear more efficient to deliver multiple messages. However, this approach needs to be balanced against the inevitable dilution of message when several are communicated at the same time. An associated reduction in effect size may be expected; a greater magnitude of effect might be achievable if only one focussed topic was chosen.

In their detailing intervention to encourage GPs to avoid the many pitfalls from polypharmacy for the community dwelling elderly, Allard communicated multiple educational messages in the hope of changing physicians’ prescribing patterns accordingly (Allard 2001). Using a primary outcome of ‘potentially inappropriate prescriptions’, it was perhaps not surprising that there were no statistically significant improvement in the intervention group because of the number of interventions recommended and the complexity of the steps involved in translating this information into practice. In a similar approach, the EBOR trialists tried to demonstrate a difference through their intervention, focussing their educational messages on multiple drug based topics. They failed to detect any difference in prescribing overall but had some measure of success in smaller practices compared with larger practices (Nazareth 2002).

Crotty aimed to reduce falls and stroke risk in the elderly, through the delivery of two (pharmacist-led) educational outreach visits to nursing and medical staff in residential care homes (Crotty 2004). The study design was a randomised controlled trial, set in Australia. As risk factors for falls and stroke are multifactorial, the investigators covered multiple topics during their two detailing visits. Outcomes included a reduction in the percentage of falls, increase in the prescribing of aspirin, and reductions in the prescribing of various psychotropic medicines. In explaining the lack of achievement of any of these outcomes, the authors cite the difficulties they found in identifying and influencing the multiple contributors (and therefore barriers to change) of care for patients in the residential care setting. These included the need to explore barriers posed by physicians, nurses, care staff and family. If Crotty had carefully considered the suggested reasons for the success of earlier detailing interventions delivered in residential care settings e.g. Avorn 1983, they might have noted the importance of anticipating barriers created by different care pathways and healthcare professionals’ or carers’ views within care homes. This may have prompted tailoring of their intervention accordingly.

Avorn and Soumerai believed there to be a need to meet with all stakeholders in order to be in a position to identify and overcome barriers to implementation of their educational messages. Similarly, researchers in Sweden held bi-monthly outreach meetings with all caregivers in nursing homes recruited in a randomised controlled trial, over a 12 month period. The aim was to test the impact of their detailing package of multiple therapeutic topics, on the quality and quantity of psychotropic drug prescribing*.* (Schmidt 1998). They managed to achieve their desired effect. They suggested that improved teamwork in the nursing homes allocated to the experimental group was a key factor. It could be that repeated contacts with staff over 12 months gave ample opportunity to reinforce the key messages, deliver feedback on improved performance and enabled stronger working relationships to develop, all of which are consistent with learning from theoretical approaches of behaviour change. Two important differences between the study designs of Crotty’s trial and that of the Swedish team were the degree of anticipation of the barriers to change and the length/intensity of the intervention. It stands to reason that anticipating barriers to change and delivering a more intense intervention will increase the chances of a successful outcome.

Goldberg conducted a randomised controlled trial comparing the effect of continuous quality improvement teams with academic detailing (Goldberg 1998). They focussed on several broad topics: hypertension prescribing, blood pressure control, depression recognition, prescribing of older tricyclic antidepressants and scoring of patients’ depression ratings using a novel depression scale. Process measures (prescribing, symptom scores) and blood pressure readings were collected. Academic detailing achieved a slightly greater effect size than the continuous quality improvement teams but neither improvement reached statistical significance. The authors concluded that academic detailing and continuous quality improvement were ineffective in improving hypertension and depression guideline compliance. Alternatively, their results may be highly conditional: the choice of multiple topics was overambitious a priori (certainly a posteriori), and a lack of anticipation of multiple barriers may have obscured the effect of the intervention.

As there are many interacting features to the complex interventions delivered in the context of the trials described above, the features cannot be viewed in isolation. However, the trend appears to favour effectiveness when fewer topics are covered. Any reduced effectiveness of the intervention as a result of covering multiple topics could be attenuated or overcome by anticipation of barriers to change and addressing these, and adhering to the detailing principles and behavioural change techniques described in the original work (Avorn 1983).

It is also worth noting that the choice of therapeutic topic is likely to have a bearing on outcomes in this type of comparative effectiveness research. From information generated by the literature search and associated reading, it appears that most published outreach trials have focussed on changing antibiotic prescribing for acute conditions, over the short term. These trials have been conducted in Australia, North America, Canada or the Netherlands (De Santis 1994; Schaffner 1983; Ilett 2000; Welschen 2004; Gonzales 1999; Zwar 1999). This choice of therapeutic topic provides frequent consultations and ample opportunity to use new approaches to prescribing, but possibly limits the ability to evaluate the impact on longer term effects or on LTC management.

**1.4.3 Number of visits**

Most trials involve a single visit to general practices or primary care centres by the detailer. While single visits might be expected to achieve statistically significant changes in short term, frequently prescribed items, uncertainty surrounds whether the changes persist. All else being equal, it follows that multiple visits are more likely to achieve longer lasting changes.

In a study by Peterson, a pharmacist visited GPs once only, after sending educational material in advance, but the duration of the effect was not evaluated beyond the end of the short intervention period (Peterson 1996).

When sufficient information on the number of visits is available, fewer visits tend to predict less chance of success. Witt delivered a single visit to encourage the uptake of an asthma guideline in general practice (Witt 2004). Using changes in sales of asthma medicines as the outcome measure, their approach failed to make an impact. While the authors discuss a few reasons for the poor uptake, their expectation was that GPs would improve adherence to a complex guideline and sustain improvements, after only one visit from an academic detailer. There was no reference to a pilot study and the sample size calculation was not described in sufficient detail to enable further appraisal of this work.

The apparent increased likelihood of effectiveness following multiple visits may need to be balanced with acceptability and cost of repeated outreach visits. Gask found that two face to face visits per year by a detailer to general practices was feasible and acceptable to GPs in Belgium (Gask 2004). Crotty found that residential care home physicians accepted two outreach visits (Crotty 2004). In delivering their successful educational package to GPs in Anglia, Fender met with practices on two separate occasions (Fender 1999). Yeo and colleagues in Australian general practice describe a controlled trial involving three visits aiming to reduce prescribing of benzodiazepines and related medicines (Yeo 1994). While they do not report any change in prescribing behavior, the GPs who received the face to face support rated their benzodiazepine prescribing as much improved but objective assessment of their prescribing did not show any change. In their discussion, the authors reflected on the components of their intervention and stressed the importance of the initial meeting to “establish rapport”. Overall, they interpreted their findings as a reminder that “we do not always do what we mean to do, and we do not always do what we think we do”. Missing in their approach was anticipation of practical barriers to changing prescribing. For example, the detailers failed to identify those patients who were eligible for benzodiazepine reduction or discontinuation, or provide the GPs with this information to facilitate change.

Soumerai appears to be the only researcher to have speculated on the dose-response of increasing the number of outreach visits. He conducted a randomised controlled trial of the effectiveness of face to face visits by clinical pharmacists, involving 435 doctors. They observed an approximate doubling of the magnitude of change in their targeted medicines when the number of visits was increased from one to two (Soumerai 1987).

In a randomised controlled trial of practice based education (using principles of academic detailing) in east London by Feder, guidelines for asthma and diabetes were actively disseminated. Focusing on practice based small group multidisciplinary educational outreach sessions, the authors visited practices on three occasions. While the duration of meetings and the interval between meetings was not reported, the effects were positive and the three visits were viewed as an integral part of the successful model (Feder 1995). The investigators appear to have adhered to the principles of academic detailing during their meetings with practices. They also encouraged practices to make improvements in the accuracy of their disease registers.

Only one report from the literature review described GP preferences regarding the frequency of academic detailing visits in practices. A feasibility study based in Belgium invited 184 physicians to receive two visits from an academic detailer focusing on Non Steroidal Anti Inflammatory painkillers. One hundred and forty two received two visits and 105 physicians gave their opinion on the visits through a questionnaire. Most (90%) respondents expressed a wish to receive an academic detailer on other topics, agreeing that two visits per year was a suitable frequency (Habraken 2003).

Another study used four, one to one visits between pharmacists and GPs in an attempt to increase the proportion of depressed patients taking antidepressants, and improve symptoms of depression (Brown 2000). The intervention made a statistically significant difference on both outcomes.

In 2002, the Cochrane collaboration considered the importance of the number of visits on the effect of academic detailing (O’Brien 2002). They reported a wide range, from once weekly for seven months (Steele 1989), to single visits (Newton Syms 1992), with examples of successful and unsuccessful outcomes across the spectrum. The review concluded on the need for standardisation and formal evaluation of the incremental importance of additional outreach visits.

The duration of effect after the last detailing visit is an important consideration, because the persistence of prescribing change will determine the extent of benefit incurred by patients. If it can be assumed that more frequent visits lead to a stronger and longer lasting effect, in designing a new intervention that builds on previous work, it makes sense to develop our understanding by increasing the number of visits and evaluating outcomes over a longer period. Therefore, we plan to trial the impact of three face to face meetings (as a balance between too few and too many) and evaluate outcomes over a longer period than any previous trials.

**1.4.4 Overcoming organisational barriers to implementation**

Organisational barriers are recognised as a key factor limiting the implementation of evidence based medicine (Haynes 1998). Some reports have highlighted practice or GP level organisational barriers as important factors limiting the introduction of prescribing change, but most investigators have not identified and addressed these prospectively. For example, none have set out to provide outreach visitors with the time and understanding to help practices with the additional effort needed to overcome organisational barriers. These might include patient identification, call and recall. A recurrent theme in the literature review thus far is that identification and addressing organisational barriers (at the level of the practice, the prescriber or the patient) is a key determinant of the success of detailing interventions.

While some detailing interventions incorporate strategies to explore and resolve GP level barriers, few apply this logic to practice level organisational barriers and fewer still to patient level barriers and supports.

Practice level barriers to implementation of (repeat) prescribing change can nullify or delay the detailer’s efforts. If we assume educational messages are accepted, even if these are implemented on a patient by patient basis by GPs, completion of this task may take a long time in primary care, if the prescribing change applies to large numbers of patients. The administrative burden involved in patient identification and engagement, arrangement of consultations to facilitate prescribing then follow up, cannot be underestimated. Additional work can be expected for patients who do not attend pre-arranged appointments. Overcoming these barriers might involve the detailer helping the practice to identify patients who would benefit from changed prescribing and set up a register or recall system which systematically invites and appoints patients.

Moher tested this approach but not within a multifaceted, complex detailing intervention (Moher 2001). He compared the effectiveness of three interventions (audit and feedback, recall to general practitioner and recall to nurse clinic) aiming to improve the cardiovascular assessment of patients in Primary Care. Establishing a nurse led call/recall system in practices and comparing the impact (for secondary prevention of CHD) on patients with CHD, they found that it was no better than audit as a means of promoting secondary prevention measures. Outcomes included reductions in blood pressure, blood cholesterol and continine levels. Each approach was found to be better than doing nothing, because assessment and follow up of patients was improved within intervention groups. The authors account for the inability of their interventions to deliver clinical improvements on GPs’ unwillingness to initiate recommended preventative medicines including statins. If their practical support was accompanied by an educational exchange, this may have helped. They found that follow up by nurses appeared to be more effective than follow up by GPs.

In other UK based studies, systematic registration and planned recall of patients for appointments was found to improve the quality of care (Feder 1995; Pierce 1989), although there are now financial incentives for practices to systematically identify and treat patients with several common chronic diseases, including CHD.

An additional, often overlooked consideration in general practice which has a direct bearing on the chances of successful detailing outcomes is the accuracy and completeness of disease registers. These form the backbone of a successful call and recall system. Accounts of the accuracy and completeness of disease registers indicate variability (as expected) (Whitelaw 1996; Jick 1991; Jick 1992; VanStaa 1994; Hassey 2001; Moher 2000). However, this evidence is not recent, and recognition of the need to improve the accuracy and completeness of disease registers and therefore reduce variation in processes of care has led to improvements, evidenced from the achievement on practices’ GMS QOF points year by year.

If the aim of educational outreach is to improve the drug management of all eligible patients with the targeted condition, the detailer needs to be aware of the potential variation in the accuracy and completeness of practice level disease registers. Support should be offered to the practice to improve this, prior to introducing changes.

The UK based qualitative study by McColl further supports the idea that organisational barriers are important in the route to the implementation of evidence based medicine (McColl 1998). In addition, the attitudes and extent of teamwork within practices was seen as a major tension which has the potential to lead to a collective failure to agree practice level policies on clinical management, scuppering practice level changes in prescribing

Going a step further to involve patients and carers (in this case parents of children aged under 6 years old), Finklelstein (2008) studied the impact of an intervention combining detailing with posted, printed educational material directed at families. On comparing this approach with usual care, they found that the use of antibiotics diminished significantly in their intervention group. The authors concluded by emphasising the importance of involving patients/carers or the public in attempts to change antibiotic prescribing practice.

In healthcare systems where patients’ ability to pay limits the uptake of health and related services, financial incentives for patients may be effective in overcoming barriers to changed behaviour (Benedetto 2000). Within the NHS these principles are unlikely to apply therefore will not be considered further.

**1.4.5 Educational outreach plus additional strategies**

As an example of how the detailing model has developed to include additional techniques, Brufsky conducted an interrupted time series study within a large health maintenance organisation covering four states in New England (Brufsky 1998). They compared a combined educational outreach and facilitation model to usual care, encouraging prescribing of Cimetidine instead of Ranitidine, for patients with gastrointestinal conditions. The prescribing of their preferred agent (Cimetidine), increased by 53.8% following the intervention. Other positive outcomes included an assessment of the rate of hospitalisations for gastrointestinal conditions. Their intervention proved effective and an accompanying economic analysis demonstrated cost effectiveness. Unfortunately, the intervention is not well described and for that reason, cannot be critiqued. The professional or other qualifications of those delivering it are also unclear, as are the content, duration, frequency of contacts, bespoke training received by people delivering it or the characteristics of patients and practices receiving it. Lack of information on these important features limits transferability. However, the authors assert that their combination of education and facilitation led to the successful outcomes. Facilitation included the use of bespoke forms to enable doctors to evaluate their own performance in relation to the prescribing of Cimetidine and the use of reinforcement strategies (e.g. feedback). In addition, the intervention team pre-screened lists of patients to clearly identify patients prescribed ranitidine who were appropriate candidates for Cimetidine. In this respect, the investigators employed marketing techniques and change methods not described previously or since then. Their methods resembled those used in everyday pharmacist prescribing support within general practices in the UK.

Nilsson and colleagues incorporated detailing with additional techniques to effect change, but unlike Brufsky, they stopped short of providing GPs with lists of patients who may have benefitted from changes to their prescribing for hypertension, peptic ulcer or dyspepsia (Nilsson 2001). Instead, they used patient level diagnoses and prescribing data from electronic patient records as part of their feedback. Feedback on prescribing rates, problem oriented educational outreach visits, educational material and the views of local opinion leaders were all used to encourage adoption of changed prescribing for the specified conditions. Within their randomised study which included three parallel intervention groups of GPs, they used before and after measures of prescribing change. Their intervention demonstrated a statistically significant improvement in prescribing.

Although New et al described their UK general practice based nurse led intervention as educational outreach, additional strategies were incorporated (New 2004). These included seeking the names of diabetic patients who were poorly controlled and providing these, together with the names of patients requiring review of their lipid and blood pressure management, to participating GPs. In contrast with Brufsky’s study, New failed to achieve a statistically significant shift in their primary outcome of pre-defined targets for control of hypertension or hyperlipidaemia in patients with diabetes. In explaining this neutral result, organisational barriers were cited as an important limiting factor. These included apparent lack of additional activity within the practice, to review those patients with raised blood pressure and lipid levels. Already overburdened staff were resistant to accepting any additional work and some practices complained of a lack of resources to deal with the additional work. Practice nurses appeared more likely to support the outreach nurses than GPs but this dichotomy appeared to cause confusion among patients. Few practices have spare capacity, ready to be deployed in this or any other way, without prior agreement or without reorganisation of existing staff or employment of additional staff.

The setting for the Brufsky study was within a US Health Maintenance Organisation. New set their study in general practice in the UK. Both studies were primary care based and utilised similar interventions, albeit within different methodologies. However, one major difference that is likely to have led to the different outcomes is the incentives of the staff for changing their prescribing behaviour. In the Brufsky study, staff pay was directly linked to cost effective prescribing and the investigators provided a rich vein of income to the practice on achievement of prescribing change. In the UK at the time of the study, improvements in control of blood pressure and lipids competed with management of other clinical conditions, pre-dating the introduction of the QOF. Prescribing in the NHS operates independently of direct market forces; therefore, additional financial reward is not a viable option.

The Cochrane Collaboration’s findings would tend to support the effectiveness of the use of financial incentives for GPs (Gosden 2005). They evaluated whether payment of primary care physicians (capitation, salary, fee for service and mixed systems of payment) impacted differently on clinical behaviour and found that there was some evidence to support the case. However, detailing models and studies are difficult to compare across different healthcare systems, because there is the possibility that an invisible hand, i.e. payment systems, can significantly influence outcomes. It is difficult to account for this confounding other than through a multicentre study.

Recognising the need to help practices identify and target patients eligible for change, Feder et al used a combination of academic detailing together with disease register support. They demonstrated effectiveness in a cluster randomised controlled trial. They acknowledged the need to update disease registers as a pre-requisite to their detailing intervention and built their novel, multifaceted intervention on a sound knowledge and understanding of the context of the UK Primary Care and general practice system of organisation (Feder 1995).

Overall, there seems to be a reasonably strong logistical argument backed up by some published evidence, for incorporating a combination of strategies, including addressing organisational barriers in addition to the original cognitive approach described by Avorn and Soumerai. However, there is no ‘off the shelf’ description or standardisation of the components of this hybrid model and it remains to be tested in the context of a trial for patients with CHD or other vascular disease, in the UK primary care system, with measurement of longer term surrogate clinical outcomes.

Practice based prescribing support pharmacists are regarded as part of practice teams or, at least, the primary care team. Therefore, the pharmacists can support eligible patient identification, call and recall, and work in the practice for longer than other visitors. In so doing, the process of the original detailing model is developed to the point where the intervention offers a balance between educational exchange and facilitation of change through direct practical support to the practice. There are other examples of the application of prescribing support models combining educational outreach with additional strategies, but none involving the following: case identification, call and recall support, more intensive, repeated intervention with pharmacists working in practices on a weekly basis. Incorporating these features represents a necessary evolution in prescribing support, potentially providing a useful means of supporting prescribing decisions as the volume and complexity of prescribing for patients with LTCs continues to increase.

**1.4.6 Cardiovascular disease focus**

Attempts at improving targeted aspects of the management of cardiovascular disease in primary care through educational outreach or academic detailing, have generated positive results (Cupples 1994; Feder 1999; Jolly 1999; McCartney 1997; Siegel 2003). However, no studies have involved pharmacists, followed patients for longer than 1 year, focussed on statins, measured impact on anything other than prescribing or described their interventions or study populations in sufficient detail. Three of the most relevant are described in Table 1.

**Table 1. Key features of selected studies**

| **Authors/**  **Country/**  **Year** | **Method** | **Participants and setting** | **Interventions** | **Outcome measurement** | **Control** | **Effect on practice and patient** | **Comments** |
| --- | --- | --- | --- | --- | --- | --- | --- |
| Avorn J, Soumerai SB. North America, 1983. | Cluster RCT | 435 physicians from a Medicaid programme in 2 states of USA. Physicians selected on basis of high prescribing of ≥ 3 target drug groups | Educational group of physicians received 2 visits from an academic pharmacist or pharmacologist over 6 months. Aiming to reduce prescribing of 3 drug groups. | Up to 9 months after intervention, measurement of number of units of drug prescribed across randomised groups. | Control and printed material only | Detailing on a one to one basis is effective and saves drug costs compared with printed materials only and control groups. Effect size: 18% drop in prescribing (p< 0.0001) | This trial was the first to implement a detailing programme and test it within a RCT. A market research consultant conducted interviews with non participating doctors, asking about their reasons for prescribing the 3 target drug groups. These insights were incorporated into the educational strategy.  The pharmacist detailers made quantitative and subjective notes of their meetings.  No information available on the characteristics of the pharmacist detailers, patients or physicians. No power calculation. |
| Freemantle, UK, 2002 | Cluster RCT | 75 general practices in 12 Health Authorities in England | Between 4 and 6 outreach visits by one of 12 community pharmacists who had received three days of training covering 4 clinical guidelines. Delivered the explicit techniques of academic detailing set out in Avorn and Soumerai’s original work. | Change in prescribing of evidence based medicines advocated in the guidelines and in one case, alternatives. Secondary outcomes are changes in trends of medicines, collected by remote assessment of prescribing statistics without linkage to patients and diagnoses. | Control practices without educational outreach for 2 of the 4 guidelines. | 5.2% improvement (95% CI 1.7% - 8.7%) in number of patients treated according to guideline recommendation.  Smaller practices responded more favourably than larger practices. | Practices were given postgraduate educational allowances for participating, which limits generalisability and questions motivation for participation.  The clinical guidelines were developed for the purpose of the trial. The results of this process are not likely to carry weight or support from participating practices because they were not involved in the production.  No measure of prescription adherence or characteristics of patients at baseline.  The authors concede that measurement of outcomes was complicated in each of the four clinical guidelines areas. Outcomes were positive for changes in 3 of the 4 guidelines; in the remaining guideline’s educational outreach programme, a decrease in guideline prescribing with an OR of 0.73 (95% CI 0.56 – 0.94), equivalent to a 3% reduction in patients managed in line with the guideline. |
| **Authors/**  **Country/**  **Year** | **Method** | **Participants and setting** | **Interventions** | **Outcome measurement** | **Control** | **Effect on practice and patient** | **Comments** |
| Diwan, Sweden, 1995. | Cluster RCT | 134 Health Centres in Sweden | 4 meetings lasting 30 minutes each, over 5 months in the practice, group outreach by a pharmacist, covering guidelines on hyperlipidaemia management | Number of prescriptions for lipid lowering drugs and prescription of first line lipid lowering drug.  Data collected from pharmacies including lipid lowering dose, strength, quantity. Age and sex were only demographics. | Usual Care | 20% increase in prescribing of first line lipid lowering drugs (p = 0.03) | Power calculation; stratification by number of prescribers and list size which is important as both can influence uptake of evidence. No measurement of cholesterol levels or adherence or clinical outcomes. No testing of model on single handed practitioners.  This trial includes a larger number of physicians than any other published. Follow up lasted 1 year after intervention finished.  No patient demographics other than age and sex; no clinical co-morbidities available.  Consideration given to existing and incident patients receiving lipid lowering treatment. |

In the study by Latour (2000), the pharmacist encouraged physicians to decrease the prescribing of lipid lowering medicines for patients at low risk of vascular disease. The primary outcome was not achieved. The study design did not incorporate a control group, was quasi randomised, did not adequately describe the intervention characteristics and had a short follow up period of five months, all of which limits the strength of the evidence. However, this work stands out as one of the few that attempted to decrease the prescribing of a medicine that is unlikely to cause any harm if continued indefinitely. In fact, over time, as subjects’ age, blood pressure and possibly other risk factors increase, it is possible that their cardiovascular risk profile reaches the point where a statin is indicated; this factor may have mitigated against the intervention working. The authors do not discuss this possibility. An important lesson from this work is the importance of carefully choosing the prescribing message to meet short term and long term patient and GP needs.

**1.4.7 Pharmacy led educational outreach**

This section considers lessons from the literature relating to pharmacist-led educational outreach, and considers direct implications for the new intervention to be tested in this study, which aims to improve statin prescribing.

In the UK, the concept of a general practice based prescribing facilitator/manager (distinct from pharmacist-led patient facing activities e.g. medication review) appears to have been first described by Leach in 1999. He suggested that to secure effective implementation of an agreed prescribing change at practice or population level, someone needs to take the initiative to modify practice computer systems, organise and send letters to patients. Since then, many different models of prescribing change have developed. In parallel, practices have developed an infrastructure that is now more able to absorb these changes e.g. the availability of practice nurses and practice managers, better trained reception staff and expert information technology solutions.

A Cochrane Review sought to answer the question of whether educational services delivered by pharmacists to physicians result in better outcomes (improved patient outcomes or decreased use or costs of health services) compared to the delivery of the same services by other healthcare professionals (Beney 2000). Insufficient evidence was available to answer this question, with only one controlled pre-post study cited. In this study, pharmacist-led delivery of outreach was compared with physician – led delivery. The objective was to reduce the prescribing of three contraindicated antibiotics and oral cephalosporin antibiotics. Physicians’ educational support was found to be more successful, although more expensive (Schaffner 1983).

Newton Syms (1992) trained pharmacists to operate as detailers, using selling techniques normally associated with the pharmaceutical industry e.g. training on communication and presentation skills, approach to GP appointments, promotional aids. Pharmacists delivered non-commercial advice on non steroidal painkillers, individually to GPs working in single handed and group practices. Their trial compared prescribing from patients registered with GPs who had received the intervention and those registered with GPs who did not. The authors found a positive shift in the prescribing of targeted medicines. However, little information was available on the baseline characteristics of the GPs or patients and the study results are not presented in enough detail to enable critical appraisal of the analysis. It appears that the impact of clustering was not incorporated into the design or analysis which is likely to have led to overestimation of the significance of the observed effect. The follow up period was 5 months post intervention. Implications of this work for the educational outreach intervention and the Statin Outreach Support study, include the need to describe the intervention and pharmacists’ training adequately, account for clustering and describe baseline characteristics of recruited practices and patients in sufficient detail.

In their design paper, Freemantle (1999) describe key features of their educational outreach intervention delivered by pharmacists across 48 practices in England but the extent to which support was targeted to call and recall was unclear. Outcomes included prescribing, summarised at practice level only. However, a lack of characterisation of patient demographics and prescribing at patient level, make interpretation of the impact of the intervention difficult.

One study involved community pharmacists in an attempt to improve the appropriateness of their recommendations for over the counter antifungal medicines. Community pharmacists received an educational outreach visit or attended a continuing professional education session (Watson 2002). This randomised controlled trial showed that there were no statistically significant differences in the appropriateness of antifungal sales from educational outreach.

In another UK based randomised controlled trial, community pharmacists delivered outreach visits to general practices (Watson 2001). The study intervention comprised posted guidelines combined with two, face to face outreach visits from community pharmacists, encouraging the prescribing of a limited list of three Non Steroidal Anti-inflammatory Drugs (NSAIDs). Comparison groups were practices with and without posted NSAID guidelines. A useful description of the intervention included the duration of each outreach visit: up to 10 minutes was allowed, and visits were three to four months apart. Watson found no statistically significant differences were demonstrable in the primary outcome. From a theoretical and pragmatic standpoint, it is difficult to envisage how this very short contact time between detailer and GP would have enabled the change process to take place. Grol mentioned the need to achieve orientation, insight and acceptance to encourage implementation of change in practice, all of which may take longer than two 10 minute slots in the context of a busy day (Grol 1992).

**1.4.8 Pharmaceutical industry models**

As with every other commercial, for profit organisation, the pharmaceutical industry spends vast sums of money on marketing. This includes supporting their representatives to engage with Doctors in Acute and Primary Care settings, disseminating promotional materials, staging conferences, carefully choosing research questions that promise to illuminate the benefits of their products in comparison with competitors, and provision of equipment to support diagnosis and prescribing. In 1994, an enquiry into the influence of pharmaceutical industry representatives on the NHS in the UK, estimated that the industry spent approximately £10,000 per GP annually on marketing. The amount spent by the NHS on delivering prescribing advice (which included pharmaceutical advisers) was approximately £500 per GP (National Association of Health Authorities and Trusts 1994). The industry makes best possible use of known influences on behavioural change. Much can be learned from their marketing techniques. For example, printed material used by representatives is often persuasive and visually appealing. Together with repetition of key points during brief opportunities, industry representatives are able to effect behavioural change in prescribing. Affability and the provision of updates, self declared “cutting edge” innovation in drug selection and small gifts all help to convey the message. Leaving free gifts may lead to an increase in the prescribing of company sponsored products, up to two years after the event (Schumock 2004; Glare 2006). In a review of the literature concerning interactions between doctors and pharmaceutical industry representatives from 1977 - 1993, Lexchin concluded that doctors’ prescribing behaviours are affected by their interactions (Lexchin 1993). This was found to be the case despite doctors’ lack of awareness of personal susceptibility to influence (Ruteledge 2003). To compound this finding, most GPs seem not to have a very high opinion of the information from pharmaceutical company detailers or company sponsored continuing medical education events (Lexchin 1993). This influence leads to increased prescribing costs (Brewer 1998), with one study showing that frequent general practitioner contact with drug industry representatives was strongly and independently associated with higher prescribing costs (Watkins 2003). However, there appears no reason why NHS pharmacists cannot adopt some of these techniques, although NHS pharmacists’ approaches are more limited in relation to provision of free gifts.

Although not clearly reported, the pharmaceutical industry occasionally enlists the support of local opinion leaders to advocate their products in the hope that they can persuade other physicians to prescribe. In primary care, prescribing is influenced by many factors. One influence for some GPs is endorsement of a medicine by a trusted consultant in secondary care, and prescription of a new medicine by a consultant leads to familiarity by the GP who has responsibility for ongoing prescribing. While the broad literature about the usefulness of local opinion leaders as a means of impacting on professional practice appears inconclusive, there may be some merit in this approach (Thomson 2005). On this basis, it appears worthy of inclusion in a multifaceted, novel prescribing support model.

Appropriate training appears instrumental to the industry’s success. It focuses less on pharmacology, more on communication skills: developing rapport, anticipating GPs’ needs, profiling individual GPs and highlighting drawbacks of competitors’ products. One study explored the complex attitudes and behaviours of groups of GPs attending reflective practice sessions focussing on their prescribing. In commenting on the skill profile of the group facilitators (detailers), the authors asserted that effective group facilitation skills to create a group process were more important than a professional background and sound knowledge of therapeutics (Watkins 2004).

One study of the interaction between GPs and pharmaceutical company representatives, helped illuminate several marketing techniques used by the industry. These included “reciprocity” in which the GP is given a gift and in accepting, feels bound to make repayment, often through prescribing of the company’s product. As pharmaceutical companies and their representatives will show bias towards their own products, the authors recommended the introduction of a third party to provide unbiased educational information about the full range of medicines for specific conditions (Somerset 2001). Pharmacists, as NHS employees, particularly those working within the practice environment, seem ideally suited to this role.

Therefore, in designing a novel pharmacist-led outreach intervention for delivery by NHS employees, an awareness and use of techniques derived from pharmaceutical industry representatives and their training programmes, are likely to support uptake of new prescribing practice.

**1.4.9 Targeted or untargeted educational outreach**

In choosing who should receive outreach or academic detailing in a trial context, researchers usually choose to target those who have the greatest scope for improvement in the intervention. In the context of routine service delivery, the decision is likely to be guided by economic principles, which leads to the same, targeted approach, by offering a service to those who need support most, thus minimising inefficient use of scarce resources. However, the decision can be more complex, e.g. the equity dimension of whether a service should be offered to all or some. In addition, in primary care, rapid access to sufficiently detailed information may not be available to inform targeting e.g. how much unmet need is there in each practice in relation to maximum guideline doses of statins for eligible patients. Therefore, targeting may not be an option.

Soumerai and Avorn targeted high prescribers of inappropriate medicines with a view to decreasing prescribing (Soumerai 1990), and many other intervention studies since then have adopted the same strategy to maximise the chances of a positive result. For example, after exposing frequent prescribers of antibiotics to a 1:1 detailing session, there was a significant reduction in the number of days of inappropriate antibiotic usage in a large teaching hospital (Solomon 2001). Other evidence suggests delivery of academic detailing without targeting may lead to insignificant changes in the outcome of interest (Hall 2001). This observation is in accord with one of the barriers to implementation of change identified by the EBOR team (Nazareth 2002): lack of perceived willingness to change among prescribers.

However, untargeted detailing has the advantage of being more easily reproduced outwith the trial setting because it does not depend on the presence of prescribing outliers, who have a recognisable need to modify their prescribing towards the mean, and it does not rely on sufficient information to inform targeting.

**1.5 The need for better evidence to underpin prescribing support**

In a systematic review of the effectiveness of pharmacists’ activities on health service utilisation, costs and patient outcomes, it was concluded that more rigorous research was needed (Beney 2000). The following features were highlighted as important and in need of improvement in future work:

- Intervention definition and description;
- Health economic evaluation;
- Generalisability of the intervention (e.g. more pharmacists delivering the intervention rather than one or two highly specialist practitioners);
- Outcomes should include clinical events or surrogate clinical endpoints;
- Design and analysis to account for clustering

Aspects of design are commonly cited as weaknesses in trials of interventions aiming to improve the professional or clinical decision making behaviour of healthcare professionals or patients (Hatoum 1993; Morrison 2001). Inappropriate designs or analyses are common and create difficulties in interpretation (Campbell 1998). Examples of this include inadequate sample size and bias in selection of practices (Avery 1997), absence of a control group (Field 1989; Green 1985; Wyatt 1992), lack of matching of controls and insufficient statistical analysis (Grant 1985), or lack of information on characteristics of the control group (Hill-Smith 1996). Studies with design weaknesses are prone to misinterpretation.

Primary care needs more implementation research because of the variability in uptake of robust clinical evidence and the negative impact on effectiveness and efficiency of patient care resulting from this (Foy 2001). To date, qualitative research involving GPs has improved our understanding of possible reasons why clinical evidence may not translate into practice (Veldhuis 1998; McColl 1998; Freeman 2001; Sweeney 1998). Together, these reports and others (Hemminiki 1975; Avorn 1982; Bradley 1991; Virji 1991; McGavock 1993; Britten 1995) suggest:

1. A wide range of factors have been shown to influence the prescribing decision making process and many of these are independent of the acceptability or availability of clinical evidence;

2. The evidence based model cannot be assumed to be robust;

3. Practical issues e.g. time constraints to learn about new evidence and systematically apply it in practice, limit the application of some evidence-based prescribing guidelines.

Therefore, in devising a prescribing support model, these points should be addressed.

**1.6 Therapeutic uses of statins in vascular disease**

Optimal drug based management of cardiovascular disease is important because it can reduce the risk of clinical events and delay early death. The management of dyslipidaemia has an important role in the reduction of these risks. Statins have revolutionised dyslipidaemia management and in the process, reduced the risk of morbidity and mortality in patients with coronary, cerebrovascular and peripheral vascular disease (Scandinavian Simvastatin Survival Study Group 1994; Sacks 1996; LIPID study group 1998; Shepherd 1995; Downs 1998; Heart Protection Study Collaborative Group 2002). The mean reduction in cholesterol was 25%, which resulted in clinically significant benefit. Contra-indications are limited to active liver disease, pregnancy and breast-feeding.

Simvastatin is regarded as the treatment of choice in the secondary prevention of CHD within the UK population (Phillips 2000). Generic simvastatin is the least expensive and can achieve cholesterol control in the vast majority of patients, if prescribed at 40mg daily dose (HPS, Scandanavian Simvastatin Survival Study Group (4S) 1994). In terms of spend; statins represent the largest drug cost to the NHS (£738 million in 2004). It has been estimated that using simvastatin instead of more expensive alternatives e.g. Atorvastatin could save the NHS £1.1 billion over 5 years (Moon 2006) without any detrimental effects. In Primary Care in Glasgow during 2005 there were more than 600,000 prescriptions for statins at a total cost of over £12 million; one fifth of which could be saved if simvastatin was used instead of the other prescribed statins. If a new approach to improving statin prescribing can be introduced, cholesterol levels at the individual level are likely to decrease. In the longer term (at 3 - 5 years after daily use of a statin) clinical events are likely to become less frequent in those treated. If 19 patients with vascular disease (Appendix I) receive a statin (e.g. simvastatin 40mg for 5 years) then it is likely that one major vascular event e.g. a myocardial infarction, will be prevented irrespective of the extent to which cholesterol levels are reduced.

As a result, many clinical guidelines have summarised these findings with a view to changing prescribing practice accordingly.

**1.6.1 The basis for clinical guidance on statins**

Prevention of atherosclerotic vascular disease requires control of all known risk factors e.g. smoking, hypertension, and glucose control. No single risk factor, including cholesterol level, should be viewed in isolation. While support for health related behavioural change (e.g. obesity, diet, physical activity, alcohol) should be offered at population and individual level, attention has focussed on statins, because they are more effective and efficient at reducing cholesterol and have the greatest measurable impact on clinical outcomes.

It is known that many more patients than those currently treated, could benefit from statins if they were offered them. However, resources are finite and in an example of the law of diminishing returns, benefits diminish as the estimated risk of suffering an event decreases. Trials which included patients at highest risk (i.e. with the highest end point rate in the placebo control arm) showed the greatest absolute risk reduction. For practical and economic reasons, the use of statins is therefore recommended only for patients who are at high risk and set to gain most.

For patients who do not have any evidence of established vascular disease, the aim is to prevent the onset of a first coronary event e.g. heart attack. This approach is called primary prevention and emphasis is given to identifying patients at high-risk of developing atherosclerotic vascular disease, as they obtain greater benefit from treatment with a statin. A coronary event rate of 30% at 10 years (i.e. 3% per year) has been advised as the threshold for treatment. It is now recommended that risk stratification should be carried out using the Joint British Societies’ ‘Cardiovascular disease Risk Prediction Charts’ (British Cardiac Society, British Hyperlipidaemia Association, British Hypertension Society, endorsed by the British Diabetic Association 1998). This is the tool recommended by the Scottish Intercollegiate Guidelines Network (SIGN; Guideline 40). Cardiovascular disease risk takes account of the risk of stroke in addition to coronary heart disease risk for primary prevention.

Secondary prevention includes patients with established vascular disease (coronary artery bypass graft (CABG), MI, Stroke, IHD, TIA, Angioplasty and peripheral vascular disease) and those with diabetes aged 40 years or above. All these groups should be offered a statin regardless of cholesterol concentrations, a recommendation based on the Heart Protection Study (HPS) (HPS Collaborative Group 2002). It was felt to have the greatest implications for immediate changes in clinical practice, and therefore, previous guidelines were updated in the year following publication of the study.

This sudden change in recommended prescribing practice created the opportunity to test a novel intervention aiming to improve the use of statins in line with the new guidance, for those at greatest risk.

**1.6.2 The case for improving Statin prescribing**

In response to growing acknowledgement of the benefits, their prescribing has increased more dramatically than most other medicines (Ramsay 2006; Bull 2003). However, this generates three linked questions:

1. Of those with established vascular disease, are all eligible patients receiving a statin at the right dose? If not, what can be done to improve uptake of adequate prescribing and dosing?

2. Can pharmacists based in practices turn their attention to improving uptake, through

a model of prescribing support?

3. Can this work be adequately described and tested, to ensure a robust answer to these questions?

Based on the review of prescribing support models described above, there are no ‘off the shelf’ interventions to ensure maximal uptake of statin prescribing guidance. This argues for innovation in the design and application of a novel intervention.

From the evidence described above, there is a robust case for prescribing statins for patients with established vascular disease. Prior to HPS, the evidence was less convincing for such a wide spectrum of patients. Publication of HPS led to reappraisal of statin guidelines and together, this is likely to account for the recent increase in statin prescribing, which has surpassed increases in any other risk reducing medicines (Bull 2003; Ramsay 2006).

This finding has important implications for the design of a trial testing an intervention to help increase statin prescribing: usual care (due to passive dissemination of guidance) is likely to improve over time and this should be factored into the power calculation. That prescribing increases over time, coinciding with the publication of new evidence, is not a new finding. Calvo and Rubinstein evaluated prescribing pre- and post- publication of successful drug trials. They monitored the incidence of new prescriptions written by physicians in primary care in a 6 month period before and after publication of trials of the following drugs: alendronate, metformin, alpha1-blockers and finasteride. Among their findings was a clear, statistically significant temporal association between prescribing and the publication of the new evidence (Calvo 2002).

However, the literature review gave a convincing account of there being shortfalls in the prescribing and use of lipid lowering agents (ASPIRE steering group 1996; Campbell 1998; Siegel 2000; Ramsay 2006; Sempos 1993; Clinical Quality Improvement Network Investigators 1995; Northridge 1994; McBride 1998; Sueta 1999; McCormick 1999; Aronson 2006; De Wilde 2003; Primatesta 2000; Whincup 2002; Minhas 2004; Smith 2001; Missouris 2001; Sloan 2001; Frolkis 1998; Hoereger 1998; Reid 2002; Majeed 2000; Fernie 2006; Pearson 2000). Many of these papers reported suboptimal statin prescribing after the publication date of the HPS. The studies varied in design, methodology, sample characteristics, country of origin and severity of cardiovascular disease. There were biases and confounding inherent in most of these due to the absence of a comparator group but the consistent finding was there being considerable scope for improvement in the identification of patients eligible for treatment, statin prescribing in comparison with the evidence base, achievement of target statin doses and achievement of target cholesterol levels.

In EUROASPIRE II, the investigators’ found half of those eligible were receiving statins and of those receiving them, half were reaching the target of 5mmol/l (EUROASPIRE II 2001). A subsequent comparison of EUROASPIRE studies I and II led to the assertion that there was a collective failure across Europe, of practice to achieve the substantial potential among patients with CHD to reduce the risk of recurrent disease and death. Approximately 60% of those patients interviewed across the nine participating countries were prescribed statins between 1999 and 2000 (EUROASPIRE I and II group. 2001). Since then, the introduction of the New General Medical Services (GMS) contract has proved that at least 60% of patients with CVD and diabetes can achieve total cholesterol levels below 5mmol/l (QOF Achievement Data 2005). However, it is not known if there can be any further improvement in uptake beyond the 60% audit target set by the GMS contract, or how many more patients would start and continue to take newly prescribed statins if the health care professionals adopted the best available implementation techniques, informed by implementation research. It is likely that there will remain a significant number of patients who will not take their statin out of choice, even if it were prescribed.

**1.6.3 Could educational outreach help improve statin prescribing?**

There is a recognised need to evaluate the effectiveness of outcomes from outreach visits in terms of clinical outcomes whenever possible (O’Brien 2002). If this is not possible, surrogate clinical outcomes could be used, particularly in cases where there is a robust link between clinical outcomes and the surrogate end point e.g. HbA1c, blood pressure, cholesterol levels. A strong link exists between cardiovascular, cerebrovascular and peripheral vascular morbidity/mortality and the use of statins (Scandinavian Simvastatin Survival Study Group 1994; Sacks 1996; LIPID study group 1998; Shepherd 1995; Downs 1998; Heart Protection Study Collaborative Group 2002). Educational outreach appears to be a promising way of delivering quality improvements in prescribing, but at present, there is insufficient evidence to enable routine application as a means of improving prescribing beyond 1 year, improving statin prescribing and clinical or surrogate clinical outcomes. Reasons for this include:

- Inadequate description of the components of interventions;
- Inadequate description of training and qualifications of those delivering the model;
- Lack of pragmatic endpoints other than prescribing
- Unknown efficiency;
- Lack of generalisability of existing evidence;
- Weaknesses in choice of methodology and analysis.

**1.7 Template for a new intervention**

In view of the above, the following are key features worth incorporating into the design and reporting of a multifaceted, new model of prescribing support:

- Utilisation of multiple strategies to effect change (Brufsky 1998);
- Face to face, brief educational exchanges with reinforcement;
- Adequate training for pharmacists, paying attention to communication skills and approaches used by the Pharmaceutical Industry (Lexchin 1993 and Watkins 2003);
- Clear definition of the components of the intervention and how they were delivered;
- Multiple intervention components including: anticipation of barriers, interactive provider education, confidential feedback, make explicit links between everyday practice and published evidence of effectiveness (Watkins 2003);
- Reminders, patient education;
- Specific focus on statin prescribing/cholesterol lowering;
- Involvement of local opinion leaders (Freemantle 2005);
- Intervention underpinned by theoretical models of behavioural change (Davis 1998, 1995);
- Delivery of the intervention to nurses and practice staff in addition to GPs (commonsense approach, recognising the dynamics of the general practice team);
- Minimum of three outreach visits, with sufficient time within each visit to establish rapport, credibility and inspire change (Yeo 1994);
- Confidential feedback on a one to one basis (Hux 1999);
- Twelve month duration of intervention with multiple meetings (Schmidt 1998);
- Provision of organisational and administrative help (Renders 2005);
- The use of pharmacists based in general practices (all previous work has involved community pharmacists or academics), with access to practice information and a peer support network between pharmacists delivering the intervention.

Key characteristics differentiating the SOS intervention from previous outreach – like interventions include:

- Non academic, non commercialised pharmacists delivering the intervention;
- Access to full patient level clinical and prescribing data;
- Access to, and influence over disease registers and call/recall systems;
- Repeated presence in the practice environment;
- Ability to meet with and influence clinical and non clinical staff;
- Three linked meetings, with sufficient time and opportunity for feedback and reinforcement between meetings;
- The use of several techniques to induce changes in prescribing practice: educational outreach tailored to the needs of the individual practitioner and practice; audit and feedback; reinforcement; reminders; social marketing; educationally influential opinion leaders; and changes to practice administration/ procedures including support for systematic case finding and call / recall;
- A single therapeutic topic combining quality improvement and cost minimisation.

**1.8 Template for a study to test a new intervention**

Review of the literature spanning 20 years indicates that more and better evidence is needed to determine the effectiveness of pharmacists in their attempts to change prescribing behaviour. The available literature is difficult to interpret because of inconsistent or nonexistent nomenclature or explanations of the interventions delivered by pharmacists. Study designs are often inadequate, trial durations and length of follow up insufficient to enable evaluation of clinical outcomes and much of the outcomes are process related. Other trials have methodological weaknesses such as lack of control groups, poor methods of randomisation, inadequate statistical analysis and problems with internal or external validity (McLaughlin 1991; Beaudry 1989).

The following features of a trial are therefore likely to add to the body of literature in this area:

- Sufficient power to detect differences in clinical or surrogate clinical outcomes;
- Applied across different general practice settings (affluent, deprived, single handed, group practices, training and non training (while it is unlikely that statin prescribing patterns differ between training and non training practices (Ashworth 2006, Mackay 2003), we considered it useful to have recruited both types of practices with examples in each arm of the study);
- An assessment of prescription collection rates as an indication of the extent of adherence to newly prescribed medicines;
- Recruitment of untargeted practices, where there is variability in the need for changed prescribing (this will help confer generalisability of the results);
- Stratification of important measures of baseline prescribing;
- Pilot work to ensure theoretical underpinning, feasibility of the model including acceptability to GPs;
- Appropriate choice of methodology and analysis to take account of clustering;
- Description of qualifications and training of pharmacists delivering the model;
- Collection of patient and practice level prescribing data at baseline and follow up, including incident and prevalent cases;
- Follow up of outcomes after 12 months (Pearson 2003);
- Link any cost savings to health outcomes (Belby 1997).

**2. Methodological considerations arising from previous, related work**

**2.1 Clustering**

In primary care based randomised controlled trials in the UK, designed to study the effect of educational outreach, patient level randomisation is neither practical nor ethical. Randomisation by group (practice) is preferable because it avoids contamination of the usual care group and the effect of the intervention can be assessed in the natural practice environment. Using individual patient randomisation in an educational outreach intervention directed at general practitioners is not appropriate because the management of one patient is not independent of another (MacLennan 2003).

Some studies involve randomisation of health professionals or groups of professionals (cluster randomisation) but analyse outcomes at the patient level, thus resulting in a possible overestimation of the significance of the observed effects (unit of analysis error) (Whiting -O’Keefe 1984; Donner 1981; Donner 2000; Simpson 1995; Divine 1992; Donner 1990), without making corrections for the impact of clustering. Pill tested the impact of a patient centred intervention on GPs (Pill 1998). Twenty nine general practices were randomised and the results analysed at practice level; prescribing and other changes were measured at the level of the patient. However; analysis did not take account of clustering so the results are likely to be an overestimate of the significance of the effect.

Therefore it is important to build this learning into the methodology of a trial of a new intervention in primary care general practices, directed at GPs.

**2.2 Pre-Randomisation and randomisation**

Stratification (balancing influential variables other than the number of practices in each arm of the study) prior to randomisation is useful if there are characteristics of practices that make them more or less susceptible to changed prescribing.

In a trial of prescribing rationalisation in primary care in the Netherlands, Van Eijk assessed some key characteristics of practices prior to randomisation (Van Eijk 2004). Three characteristics independently influenced their main outcome measure: practices’ use of prescribing feedback data, a formulary and a consensus on drug choice within the practice. Consequently, they stratified on this basis.

As there may be differences in the organisation, response to educational interventions and uptake of evidence based medicine between single handed and group practices, stratification by practice size has been recommended (Soumerai 1989). In keeping with this, Nazareth found disproportionately increased uptake of their outreach intervention in smaller practices compared with larger practices, supporting the case for stratification of this potentially important confounding variable (Nazareth 2002).

Van Eijk (2001) found more successful outcomes from group practice detailing, in their trial designed to compare the impact of both group and individual detailing on the use of highly anticholinergic antidepressants.

These two reports draw conflicting conclusions on the effect of detailing in group versus smaller practices. They perhaps underscore the relative importance of heterogeneity of context, intervention duration, delivery, intensity and topic on the outcomes, and the difficulties inherent in comparing different trials using similar interventions in different healthcare settings. This emphasises the importance of adequately describing these variables in order that other researchers and policymakers can compare with their own healthcare settings and draw their own inferences.

**2.3 Recruitment and generalisability**

A description of the process of practice recruitment in detailing type trials is useful because it enables an understanding of the representativeness of participating practices and informs the success of different recruitment methods, for other researchers.

Moher (2001) initially contacted eligible practices in writing. After two invitations, non respondents were telephoned to assess their willingness to participate. From 79 eligible practices, 21 (27%) agreed to participate and were randomised. Fifty eight practices did not respond to the initial written invitation, were not interested or had other reasons not to participate. It is not clear over how long recruitment lasted, how much resource was required to follow up non responders or whether non respondents were contacted in person.

Nazareth managed to recruit 75 of 102 eligible practices in their Evidence Based Out Reach (EBOR) trial (Nazareth 2002) however, further details on how they approached practices is lacking.

Van Eijk (2001), in common with most other trialists, do not describe their recruitment process. The lead investigator was known to the practices and it is likely that this helped.

Hall (2001) offered either a single outreach visit or audit and feedback to 38 practices in the intervention arm of a randomised controlled trial aiming to improve the drug based eradication of Helicobacter pylori infection in Primary Care. At the time of recruitment (1996), all practices in their catchment area were invited and ethical committee approval was not required. No detail on their recruitment process was given.

The transferability and generalisability of detailing studies is increased if the people delivering the intervention are less highly qualified, or representative of the majority of pharmacists practising in the community. Most randomised controlled trials involving pharmacists have recruited those with highly advanced training and postgraduate degrees (McMullin 1999; Lipton 1992). This drawback applies to the original pharmacist-led educational outreach work by Avorn and Soumerai in 1983, and, analogous to the targeting of outlying prescribers for intervention, specialists delivering outreach interventions will increase the chances of success, but reduces the generalisability of the results.

From an NHS perspective, using a more representative sample of the pharmacy workforce to deliver an intervention would increase the implementation potential. Therefore, an important consideration in designing the intervention is that the pharmacists have some additional training but are not so different from other prescribing support pharmacists that the intervention cannot be reproduced.

**2.4 Choice and measurement of outcomes**

Evaluating prescribing change in relation to set prescribing criteria generates a process rather than an outcome measure of quality.

In most studies involving guideline implementation, it is assumed that care is improved when a guideline is implemented. A direct, proportional relationship between prescribing change and guideline criteria might be assumed to result in improvements in quality of care. However without measuring health outcomes, it is difficult to confirm and quantify the effect size, yet a neutral or negative result from this type of intervention may represent an adequate response from the intervention but inappropriate guideline recommendations. Process outcomes may therefore be more appropriate for trials of health care delivery. They may be considered more sensitive indicators of quality than clinical outcomes because poor outcomes do not always result from poor processes (Brook 1996).

When there is a proven impact on clinical outcomes from improvement in prescribing in a clinical trial setting, it is likely to be specific to a particular health care setting (Thomas 2000). To date, there is insufficient evidence to link educational outreach visits to reducing the number or prolonging the time to hospitalisation or impacting on mortality. Therefore, evaluating the effectiveness of outreach visits in terms of clinical outcomes is desirable (O’Brien 2002). If this is not possible, surrogate clinical outcomes could be used, particularly in cases where there is a robust link between clinical outcomes and the surrogate end point, e.g. cardiovascular, cerebro- and peripheral vascular morbidity/mortality and cholesterol lowering or simvastatin prescribing.

On the other hand, it might be argued that given the unequivocal efficacy of some medicines e.g. Statins, it is an unnecessary duplication of effort to follow patients to the point where differences in clinical outcomes become apparent. ‘Care as usual’ as the control intervention is one acceptable way of dealing with this scenario.

One of the lessons learned from the cluster randomised controlled trial of educational outreach conducted by Gask (2004), included the usefulness of measuring patient level outcomes. Participating GPs received an educational intervention aimed at improving the process of assessment and management of depression. Patients with depression presenting to GPs who had received this intervention were evaluated for their depression status and satisfaction with consultations. The only positive outcomes in the study were patient reports of the improved ability of intervention group GPs to listen and understand their symptoms better. Most studies do not assess this type of patient oriented outcome.

The study by Solomon is also atypical in that it measured clinical outcomes. In their trial of antibiotic detailing in a hospital setting, they captured the impact on length of stay, intensive care unit transfers, readmission rates and in-hospital death rates. These were similar in groups of doctors receiving detailing and those with no support (Solomon 2001). However, their study was not powered to detect differences in any of these outcomes; the primary outcome was a reduction in prescribing of non-formulary intravenous antibiotics and this was achieved.

An unusual feature of the study by Freemantle (Table 1) was the choice of outcomes. The researchers evaluated three steps considered pre-requisites for the achievement of the primary outcome. The steps were general practice agreement to participate in the study, attendance at outreach visits and the GP’s prescribing practice. While the trial did not show a statistically significant change in prescribing overall, their unusual choice of outcome measurement enabled a better insight to the reasons for the lack of impact, rarely seen in a randomised controlled trial design.

An additional consideration in the choice of outcome relates to whether new (incident) or established (prevalent) prescribing is evaluated at follow up. Very few trials evaluate both; most focus only on incident prescribing, presumably because it is a more sensitive indicator of effect and easier to measure. While this is a legitimate approach, it perhaps lacks the comprehensiveness of measuring both. A greater effect size is likely if a larger number of patients receive the targeted changes, which means all those patients who are eligible and already receiving medicines for long term conditions should be considered suitable for prescribing change. In addition, measuring impact on both incident and prevalent cases is likely to be of more interest because both groups require support. As a demonstration of this point, in their trial of educational outreach aiming to reduce benzodiazepine prescribing, Zwar considered both, but detected a reduction over time in only the maintenance (prevalent) prescriptions for benzodiazepines (Zwar 2000). In contrast, GPs described more barriers when attempting to change maintenance treatments for asthma rather than incident treatments in one randomised controlled trial based in the Netherlands (Veninga 2000).

One study aimed to evaluate the impact of two different methods of implementing guidelines on the management of hyperlipidaemia. The comparisons comprised guidelines displayed in general practice case records in an algorithmic format or in a standard (non algorithmic) format. Unusually, prescribing or cholesterol changes were not evaluated. Instead, the authors chose to ask participants’ opinions of their preferred guideline display and which method led to the most prescribing changes. A key finding was that there was a statistically significant mismatch between the perceived and actual proportion of patients managed appropriately between groups. However, as patient level and practice level prescribing data was not collected in sufficient detail, no more inferences can be made (Nguyen 2000).

Little is known about the longer term outcomes from detailing research. In the original trial testing academic detailing against passive dissemination of printed materials, the effect waned after nine months (Avorn 1983). Other evidence suggests effects weaken after 12 months (Pearson 2003) but no studies could be found where follow up lasted beyond 12 months. This argues for designing a study involving educational outreach with sufficient power to detect a difference in clinical outcomes over the longer term.

Figueiras (2001), showed that one to one detailing using reminders and focussing on only one (narrow) topic was effective up to nine months post intervention. Only two studies from thirty four identified through a systematic review in 1997 found that changes to clinical behaviour including prescribing were sustained beyond nine months (Beilby 1997). Until an empirical evidence base is clear, it could be argued that outreach visits are not the method of choice if longer term change is required (Tamblyn 1997).

The Anglia menorrhagia education study (Fender 1999), aimed to determine whether an educational package could influence the management of menorrhagia, increase the appropriateness of the choice of non - hormonal treatment and reduce referral rates from primary to secondary care. One hundred general practices were recruited within a randomised controlled trial. The intervention resembled academic detailing and the principles adopted by the independent academics were borrowed and acknowledged as being from Avorn and Soumerai’s original work. Results were positive for two of the three outcomes: significantly fewer referrals and higher use of tranexamic acid but no decrease in the use of norethisterone in the intervention group practices compared with controls. While the trial was the first to use academic detailing in the area of menorrhagia management, the process for measurement of outcomes is a weakness in the trial design. GPs in both groups were asked to place a self report pad in a prominent place on their desks, and complete it following consultations with suitable patients. This approach ran the risk of under reporting for GPs within practices allocated to the control group who have not received any educational outreach support. There was no assessment, or reporting, of the true, or baseline, prevalence of menorrhagia across the practices. A practice computer search for prescribing of the implicated medicines and a practice Read code search for menorrhagia diagnosis before, during and after the intervention in each participating practice could have addressed this potential source of bias. It is also not clear whether the one year follow up period commenced at the second of two outreach visits or at some point thereafter.

Only one study could be found which was designed to explore long term outcomes from a detailing approach and a clinical endpoint. The trial design used historical and parallel comparator groups of practices; lack of matched controls and absence of randomised design limits the usefulness of the results (May 1999). However, doctors received intermittent visits from detailers (pharmacists with teaching hospital experience) over a five year period, with the observation and data collection period spanning 11 years. With the outreach topic of ‘safer prescribing of Non Steroidal Anti-Inflammatory painkillers’, the research team from Australia measured the number of hospital admissions for gastrointestinal problems and found a profound (70%) reduction in the patients from practices continuing to receive outreach visits. What is surprising about this study is the magnitude of the effect: a 70% reduction over a long time period is an incredible achievement, but because of the way the researchers interacted with their GPs, is understandable. They gathered clinical and practice level evidence, weighed it up against other evidence, honed it by involving local experts and opinion leaders. Overall, a pragmatic study of this size is difficult to deliver, but due to the lack of randomised control group, the outcome remains uncertain.

Overall, it is preferable to measure clinical outcomes whenever possible and the longer the follow up the better, if we are to generate definitive evidence of the longevity of the detailing effect. Most work has not addressed either, therefore both will be considered in the present research.

**2.5 Economic appraisal**

Insufficient and inadequate economic evaluation of educational interventions creates uncertainty in the decision to adopt trials of implementation research with positive results (Brown 2002; O’Brien 2002; Beney 2004). Based on available evidence, there is insufficient information indicating cost effectiveness. However, Soumerai evaluated the cost effectiveness of academic detailing within a randomised controlled trial and found it to be highly cost effective. Two meetings lasting approximately 18 minutes each, between a trained detailer and GP, were focussed on three drug groups commonly used inappropriately. They found that target drug use was decreased beyond the point where the model became cost effective. They also observed that the reduction was not affected by pre-intervention prescribing levels (Soumerai 1986).

Watson delivered educational outreach visits to GPs in England and found the costs of delivery were greater than the costs saved as a result of the (statistically insignificant) prescribing changes observed (Watson 2002). However, in this study based in 20 general practices in England, the team delivering the intervention did not explicitly make any recommendations of a cost saving nature which is unusual for pharmacist-led prescribing support in the UK. Without cost saving as one of the key outreach messages, and without an endpoint associated with significant costs (from an NHS perspective) e.g. hospitalisations, it is less of a surprise that cost effectiveness was unproven. May et al also found this to be the case in 1999.

Possibly the most detailed study of the cost effectiveness of outreach visiting was described by Mason (2001). Their perspective was that of the policymaker faced with the decision to implement guidelines on ACE inhibitors for heart failure and tricyclic antidepressants rather than newer, more expensive SSRI antidepressants. While their analysis, like the original publication (Nazareth 2002), is at times difficult to follow, it appears that their model of outreach became cost effective if the focus was on improving quality (increasing ACE inhibitor use in heart failure) but not for cost saving (tricyclic instead of SSRI antidepressants). Key reasons behind this were discussed and included the large health benefit achievable through the use of ACE inhibitors in heart failure while the relatively small cost savings accrued by switching antidepressants did not offset the cost of the outreach visits. Acknowledging that the process of implementation of best practice in prescribing incurs a cost, Mason summarised the importance of evaluating the policy cost effectiveness of prescribing change models, and argued for the measurement of costs to form part of implementation research design.

**3. Study methods**

**3.1 Setting - NHS Greater Glasgow & Clyde**

At the start of the SOS trial (2003), NHS GG&C existed as two geographically and organisationally separate Board areas: Greater Glasgow Health Board and Argyll and Clyde Health Board. In 2006, Argyll and Clyde Health Board split and the Argyll area merged with Highland Health Board. Greater Glasgow Health Board absorbed the Clyde component, and was renamed GG&C Health Board. The SOS trial involved practices and policies within Greater Glasgow only.

NHS GG&C Health Board provides health care to almost 25% of the Scottish population. The GG&C population lives within a diverse geographical area, encompassing both urban and rural settings and suffers high levels of deprivation and unemployment with the worst health status and most extreme health inequalities in Scotland. Although overall life expectancy is increasing, the population faces a growing burden of morbidity and disability driven by LTCs. In addition, the Board area continues to attract an increasing migrant population, with their own health challenges. LTCs account for over 80% of total general practice consultations and 60% of hospital bed days (Department of Health 2004). Cardiovascular Disease (CVD), cancers and chronic respiratory disease account for the majority of premature deaths. In the Board’s Director of Public Health Report (2009), the following key health problems were identified:

- CVD;
- Health inequalities;
- Substance Misuse (drugs, alcohol and smoking);
- Obesity.

A substantial proportion of CVD risk is attributable to high cholesterol and some of this is preventable through prescribing of statins in accordance with robust evidence from clinical trials. CVD encompasses several distinct conditions e.g. Coronary Heart Disease (CHD). Other conditions sharing the same vascular pathophysiology include stroke, Transient Ischaemic Attack (TIA), Peripheral Vascular Disease (PVD) and Diabetes. Underlying coronary vascular disease is the most common reason for Coronary Artery Bypass Graft (CABG) and Angiography, which classifies patients who have undergone these procedures as being at equivalent risk as patients with CHD. CHD is the largest subgroup of CVD and as such, is a strong predictor of CVD trends. A summary of the combined prevalence and impact of vascular disease within GG&C was not available. However, age standardised CHD death rates were available and are described in Fig 1. While the prevalence of CHD is decreasing, in GG&C Health Board it remains the leading cause of death, with rates consistently above the National average (Figure 1).

**Figure.1 Coronary Heart Disease Death rates in NHS GG&C**


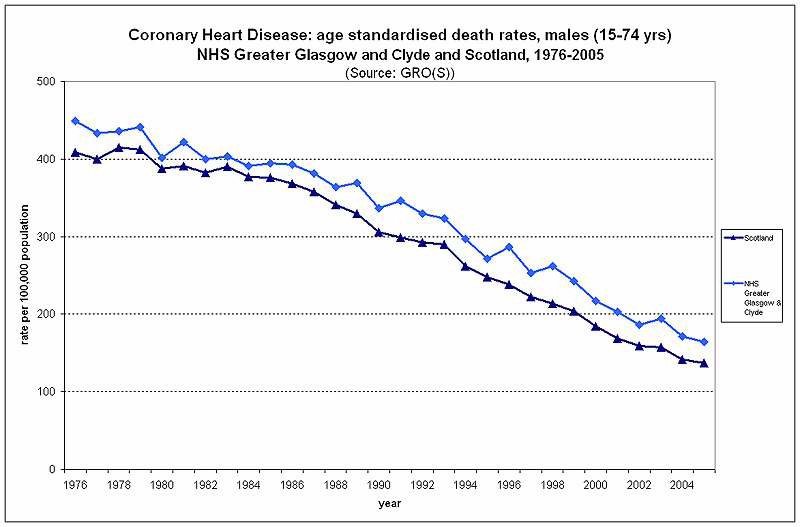


Several factors could have accounted for the steep decline in death rates in GG&C and the rest of Scotland over this period. These include improved primary and secondary prevention of CHD e.g. improved prevention and management of hypertension or myocardial infarction (Capewell 1999). Decreases in blood pressure over this period were confirmed by data from the Scottish Centre MONICA studies, with additional data showing reductions in smoking prevalence and cholesterol levels between 1976 and 1996 (Evans 2001).

The Heart Protection Study and derived GG&C guidance offered an opportunity to further tackle the burden of CHD and other vascular mortality for patients with vascular disease, because of the finding that simvastatin 40mg daily for 5 years reduced the rates of heart attack, stroke and revascularisation by approximately 25%. While cholesterol decreased in HPS, the use of simvastatin 40mg was sufficient to achieve benefit without cholesterol reduction.

The challenge for GG&C and other health care providers is to maximise the uptake of robust evidence based treatments such as this.

**3.2 Aim of study**

The aim of the study is to evaluate the effectiveness of pharmacist-led SOS intervention delivered at the level of general practices, to practice staff. The SOS study will therefore test the hypothesis that primary care, general practice based pharmacists delivering the SOS intervention at practice level, can increase the proportion of patients with their cholesterol controlled, compared with care as usual.

**3.3 Endpoints**

The primary endpoint is the proportion of patients with cholesterol controlled, evaluated at the level of the individual patient, in the SOS intervention arm practices compared with Usual Care (UC). Secondary endpoints are:

- Prescribing of simvastatin;
- Prescribing of Simvastatin 40mg with cholesterol control;
- Prescribing of simvastatin 40mg;
- Cholesterol tested since randomisation for patients prescribed simvastatin 40mg;
- Cholesterol levels of patients prescribed simvastatin 40mg;
- Cholesterol levels tested since randomisation;
- Cholesterol levels;
- Prescribing of any statin.

Outcomes will be assessed between 1.4 and 2.2 years (mean 1.7 years) after randomisation.

**3.4 Sample size and power**

Cluster randomised trials require larger numbers of individuals because there tends to be correlation of outcomes within clusters which otherwise reduces the statistical power, compared to individually randomised trials. The method of calculation of sample size is a key difference between a practice randomised trial and an individually randomised trial: the sample size needs to increase to accommodate clustering, using a cluster inflation factor (also called the design effect, D). The design effect is related to the cluster size (m, the number of patients in each cluster) and the intra cluster correlation coefficient (ρ, which measures the size of the clustering effect i.e. the correlation of patient outcomes within a cluster) by the equation:

D = 1 + ρ (m – 1)

The design effect enables calculation of the amount by which the sample needs to be multiplied, to maintain sufficient power.

**3.5 Statistical analysis**

Pilot work found 50% of patients to have cholesterol controlled with an intraclass correlation (ICC) of 0.4. Assuming that data for 40 patients per practice would be collected, we estimated that approximately 20 practices (10 per group) would be required to detect an increase in cholesterol control to 90% in intervention practices. We anticipated a high practice dropout rate due to the length and intensity of the intervention. We, therefore, invited 49 practices to participate.

Analysis will be by intention to treat. Descriptive statistics for continuous variables will be presented as mean and standard deviation and for categorical variables, as frequencies and percentages. Baseline analysis will compare participating with non-participating practices, SOS with UC practices, and single handed with group practices. Patient level data will not be collected for non-participating practices. Summaries will use continuous and categorical variables, as appropriate. Practice-level data (e.g. number of GPs, list size) will be compared using the Wilcoxon-Mann-Whitney test, or, for comparisons between SOS and UC practices, using the Wilcoxon Signed Rank test within pairs of practices.

Due to the non-normal distribution of cholesterol levels, a log-transformation will be applied. Patient level data will be analysed at the individual level for greater power.

Linear and logistic regression models will be used to test for differences in the primary and secondary outcomes between the two intervention groups. These models will be adjusted for practice pair to account for the matching at randomisation. Results will be presented as intervention effect or odds ratio, 95% confidence interval and p-value. Secondary analyses will be carried out to investigate the sensitivity of the results to adjustments for age and sex. A sensitivity analysis, adjusting for any statin prescribing at follow up, will be carried out for the primary outcome to assess whether the treatment effect can be explained by increased statin prescribing.

Subgroup analyses of differences in endpoints between SOS and usual care groups will be defined by the following baseline variables: age, gender, practice level socioeconomic deprivation (≤ median, > median), practice type (single handed or group), patient type (prevalent: known to the practice at baseline or incident: new diagnoses after baseline date), number of co-morbidities (one vs. two or more), presence or absence of IHD, prescribed or not prescribed a statin and cholesterol controlled or uncontrolled at baseline.

**Fixed and Random effects models**

With multiple measurements from the same practice and pair, regression analysis will be used to investigate the relationship between the response variable and one or more predictors. Fixed effects regression models assume the only source of variability is between subjects (and that this was constant across all practices); adjustment will be made only for the pairing of practices. However, the data is not independent (patients within practices and pairs are more likely to be similar to one another than patients from different practices and pairs). Random effects regression models treat practice pairs as random effects, taking into account two forms of variability: within practice/pair and between practice/pair variability.

Either fixed or random effects models can be used; fixed effects will be reported.

**3.6 Ethical approval and study registration**

Ethical approval was granted in December 2002 (Appendix II). At that time the intervention was called ‘clinical facilitation’. A cost effectiveness analysis is planned to report after 10 years of follow up. As the unit of intervention, all practices provided written consent to participate. The study is registered: ISRCTN61233866.

**3.7 Funding**

NHS Greater Glasgow and Clyde. The study design, funding and governance are independent from commercial sponsorship of any kind.

**3.8 Participating practices and patients**

Practices will be asked to identify patients who had confirmed vascular disease. These patients are at ≥ 30% risk of suffering a cardiovascular, cerebrovascular or peripheral vascular event in the next 10 years. In accordance with GG&C Cholesterol Guidelines (Appendix III), this level of risk will be confirmed by the following confirmed diagnoses:

- Myocardial infarction;
- CABG / angioplasty;
- Angina/IHD;
- Angiographic coronary artery disease;
- Stroke/transient ischaemic attack;
- Peripheral ischaemic arterial disease/intermittent claudication;
- Diabetic patients aged over 45years.

A validated computer search of each general practice’s computerised patient record will generate a list of eligible patients. Each patient’s computerized and paper record will then be screened to collect relevant data.

Due to time and resource limitations, a systematic sample of every third eligible patient’s data will be collected in 5 practices with the largest list sizes (12330, 7354, 7215, 7099 and 5616 patients). Therefore, cross sections of data will be collected at baseline. In addition, in all practices, some demographic data (in particular, the “Patient identification number”) may not be available. Both factors preclude longitudinal follow up of all patients. In all practices, data will be collected from each eligible patient’s record, anonymised in the practice and entered onto an ACCESS database.

**3.9 Randomisation**

**Sequence generation**

Due to the relatively small number of recruited practices and practice level randomisation, only two strata will be used (Pocock 1983). To maximise the chances of practices in the intervention and UC arms of the study being comparable at baseline, we will separate practices into prognostic factors thought to impact on the primary endpoint: Single Handed (SH) or Group (G, more than one full time GP) and the proportion of eligible patients whose cholesterol is controlled at baseline. This distinction will be made because we suspect that larger practices are more likely than single handed practices to implement the SOS intervention. Though we are not aware of an evidence base surrounding this, we suspected that Group practices would have three features favouring better uptake of the SOS intervention:

1. Multiple partner practices would have more advanced record keeping, systematic case finding, call /recall systems;

2. Greater capacity and flexibility to absorb additional work incurred by the intervention;

3. More spare rooms, enabling a pharmacist to work with the practice team on the same day each week. This would tend to increase the chances of the intervention working.

To minimise imbalance between SOS and UC practices in proportions of patients with cholesterol controlled, our framework had this as a secondary division. We used the ratio:

| | Number of eligible patients with cholesterol in target range | | --- | | Number of eligible patients | |
| --- | --- | --- |

Within their strata (SH or G), practices will be arranged in ascending order according to this ratio, ordered then numbered sequentially, and paired (practice 1, practice 2), (practice 3, practice 4) etc.

This approach to randomisation will minimize the differences from these prognostic factors, between treatment groups, to validate the assumption that any differences seen in the outcomes were most likely due to differences between the SOS intervention and UC and not random imbalances in baseline characteristics between practices in the SOS and UC arms.

**Allocation concealment**

A table of random numbers (MINITAB statistical software) will be used with one practice from each pair randomly allocated the number “1” and the other allocated “0”. Practices will not be identifiable by name: the practice’s unique Community Health Index number will be used as the only identifier, until after randomization is complete. This process ensures a balanced allocation of practices between SOS and UC arms in respect of Practice status (SH or G). This type of matched cluster design (‘matched pair’ in which one of two matched clusters in a stratum are randomly assigned to each intervention) is frequently adopted in cluster randomised trials (Donner 2000). The allocation was therefore based on clusters rather than on individuals, and the identity of all the practices will be concealed until after allocation to SOS intervention or UC.

**Implementation**

The allocation sequence will be generated by Alex McConnachie (AMcC). Richard Lowrie (RL) will assign practices to the SOS intervention or UC.

**Blinding**

In common with trials of other educational interventions, double blinding was not possible. The study will be assessor blind, because baseline data will be collected before allocation. At follow up, two independent researchers will collect prescribing and cholesterol outcome data. Both will be blinded to whether practices had received the SOS intervention or UC, therefore bias in the process of data collection will be minimised.

**4. The Statin Outreach Support intervention**

Appendix IV summarises the process of the SOS intervention. This summary will be used as an aide memoire by practice staff. Pharmacists delivering the SOS intervention will introduce, discuss and reinforce the main messages from the cholesterol guideline during three face to face meetings between December 2003 and December 2004. Between meetings, the pharmacist will work in the practice one day per week, to understand and whenever possible, improve the practice’s attempts at introducing a systematic approach to offering simvastatin 40mg to eligible patients. The gap between meetings will be approximately 4 months, depending on the availability of the GPs, size of the practice and pharmacists’ annual leave.

**4.1.1 Overview**

Pharmacists will identify the names and contact details of eligible patients (who are not prescribed simvastatin 40mg). The pharmacist will then help the practice to agree on whether, how and when to introduce a new prescription for simvastatin 40mg for eligible patients with established vascular disease, on a case by case basis. Pharmacists will encourage GPs to adopt and apply the same approach to statin use (i.e. guideline based) for incident patients as they encountered them during routine practice.

**4.1.2 Before the first meeting**

Pharmacists will be asked to work in their allocated practice one day each week for approximately one year. They plan to access, collect and summarise patient level information to confirm that each patient who is eligible for a statin is offered one, or is currently prescribed a statin, and if not, reasons recorded. This involves confirmation of eligible diagnoses, statin prescribing (current and historical), prescription ordering, cholesterol tests and levels. Practice disease registers will be validated and updated or amended where necessary. Confirmation of diagnoses will be sought by looking through case notes, computer records or hospital (including Accident and Emergency) discharge letters.

Reasons for patients not receiving a statin will be collected. From pilot work, these are likely to be due to failure of the practice to offer the patient a statin; the patient defaulting routine appointments; dropout from prescription ordering; a history of statin intolerance or contraindication. Some eligible patients may be overlooked by their practice. In this case, the pharmacist will be asked to reinstate the patient onto the relevant disease register, in order that they would be subject to routine call and recall for review by the practice, or contacted with the offer of statin initiation. In pilot work, pharmacists noticed that patients had moved house but the new address had not been entered onto the practice computer system, meaning the patient would not have received notification of the need for an appointment at the practice if a letter had been sent.

Pharmacists will be asked to use the time in the practice to collect and summarise eligible patient level information on diagnoses, statin prescribing history and cholesterol levels. Baseline information (collected prior to randomization) will be used as a starting point for this process. Pharmacists will be asked to identify prescribing traits and gaps in care e.g. lack of cholesterol levels which limited the practices’ ability to better manage eligible patients. The prescribing traits of individual GPs will be recorded whenever possible, to provide a rich source of information to individualise attempts to improve prescribing.

Pharmacists’ perceptions of organisational barriers to increasing the uptake of simvastatin prescribing will be recorded. The pharmacist will form a plan to support the practice in finding ways to engage and implement the key messages of the educational sessions.

**4.1.3 Meeting one**

Each pharmacist will receive clear instruction to address the following three objectives during meeting one:

- enable relationship building between pharmacist and practice staff;
- evaluate the GP or nurse’s awareness and understanding of the HPS trial and statin prescribing guidelines;
- identify perceived barriers to changing statin prescribing in line with guidance.

This face to face interactive discussion will include a power point presentation, involving the pharmacist and each GP, nurse and other practice staff.

The first meeting will help the GP or nurse understand the SOS intervention process. An open, honest exchange will be encouraged. The pharmacist will use open questions to explore the GP or nurse’s usual statin prescribing practice. For example, asking which statin was prescribed first line, at what point statins were initiated (what was the trigger for initiating a statin), whether patients were assessed for a statin (and other secondary preventive medicines) opportunistically or through planned appointments, was an up to date cholesterol and whether the GP believed that liver function test results needed to be present before statin prescribing. The pharmacist will ask about the GP or nurse’s awareness and views on the recent Heart Protection Study and local guideline.

A summary of the practice’s statin prescribing (numbers of patients, statin type, dose, trends over the past few years, cost), will be shared and discussed during the meeting. Other practices’ prescribing will be used as a comparison. Pharmacists will ask GPs and nurses if they wish to hear the views of ‘educationally influential’ local opinion leaders and if so, who these opinion leaders are. The pharmacist will offer to contact these opinion leaders, with a view to feeding back answers to the GP/nurse during the second meeting. The group of pharmacists will be encouraged to contact each other and if similar questions arose from GPs or nurses in other practices, share learning across the network.

The pharmacist will be asked to establish, from each GP and nurse, what they considered to be their own and their practice’s key barriers to systematic offering of simvastatin 40mg to each (eligible) patient not prescribed it. If this discussion progresses well, targets will be agreed, for the desired proportion of patients with statins and cholesterol controlled, in their practice, at the end of the intervention. Benefits to some GPs may be conceptualised in terms of achievement of contract points and therefore remuneration for the practice, or the prospect of systematically offering evidence based prescribing for patients at highest risk. Cost savings will be projected, in view of the lower cost of simvastatin compared with other statins (one month’s supply of the preferred SOS statin (generic simvastatin 40mg) was £1.32; a commonly prescribed equivalent was Atorvastatin 20mg once daily, costing £26.64 per month).

**4.1.4 Between the first and second meetings**

Pharmacists will be asked to use all of the material they obtain from their previous time in the practice, to prepare a tailored learning/action plan for each individual GP/nurse and the practice. A printed report of the plan will be circulated after meeting two.

While the content of this report is likely to vary between practices, pilot work indicated that the core practical recommendations were likely to include the practice (with support from the pharmacist) categorising eligible patients into one of the following groups:

1. Not prescribed a statin

2. Receiving simvastatin but dose suboptimal;

3. Receiving simvastatin 40mg;

4. Prescribed a statin other than simvastatin, optimal dose;

5. Prescribed a statin other than simvastatin, suboptimal dose.

In each case, cholesterol levels were controlled, uncontrolled or unchecked.

Appropriate actions will be mapped to each category of patient e.g. for those prescribed a suboptimal dose of simvastatin, the pharmacist will recommend each patient should receive a letter with a new prescription for simvastatin 40mg, barring contraindications. Based on the pharmacist’s impression of the practice’s repeat prescribing organisation and the willingness of GPs and nurses interviewed, the pharmacist will suggest who should be responsible for each agreed action. The pharmacist’s role in this process is to provide a concise summary of the evidence base and consensus opinion where required. Together with practice staff, the pharmacist will screen case notes, noting and recording the category of each patient and updating the practice’s disease register. Practices will be asked to continue the process of patient identification after the intervention period. In this way, eligible patients will include existing and incident patients.

The pharmacist will therefore plan appropriate actions and responsibilities for practice staff and match these to each category of patient. Table 2 describes options from a template plan.

**Table 2. Possible actions following a SOS intervention meeting**

| **Category of patient with vascular disease** | **Intervention by practice team (pharmacist, named nurse, named GP)** |
| --- | --- |
| Prescribed low potency statin,^§^ cholesterol and LFTs known and at target | Switch to simvastatin 40mg by contacting on phone then confirming by letter |
| Prescribed low potency statin, cholesterol and LFTs not known/not at target | Letter for blood test then switch to simvastatin 40mg |
| Prescribed potent statin, cholesterol not at target | Phone call to discuss concordance |
| Prescribed potent statin, cholesterol and LFTs not known/not at target | Letter for blood test then increase dose if necessary |
| Prescribed potent statin, cholesterol at target | No action |
| Prescribed low potency statin, cholesterol at target | No action *  Check LFTs |
| No statin; LFTs and Cholesterol known | Letter for appointment for consideration of statin |
| No statin, LFTs and Cholesterol not known | Letter for appointment for bloods then discussion of need (GP/nurse), leading to Statin prescription |
| Started on simvastatin 40mg (or other sufficiently potent statin), cholesterol not known | Letter for appointment for bloods |

^§^ Potency relates to the cholesterol lowering ability of the statin. Low potency statins include Fluvastatin and Pravastatin. Potent statins are all others e.g. simvastatin, Atorvastatin, Rosuvastatin. * Some practices decided, on the weight of the HPS evidence base, to substitute low potency statins with simvastatin.

Pharmacists will quantify the number and confirmed the names and contact details (address, phone number) of patients fitting each category, then ask the practice to agree a follow up date and to name the person responsible for ensuring action.

Workload implications for the practice will be carefully considered by the pharmacist in advance of meeting two. Key anticipated actions include time to generate letters to eligible patients, setup a call/recall system, consultation time and time to enter new information onto the practice computer system to ensure long term follow up and sustainability of the changes. The pharmacist will anticipate these additional tasks and suggest ways to overcome them. Possible approaches include the practice dividing the list and allocating part of it to each GP to action, or reception staff agreeing to call patients for an appointment, booked for the GP during any quieter slots. The pharmacist will prepare an interactive plan on power point, with concise graphic material.

**4.1.5 Meeting 2**

Pharmacists will ask practices to protect approximately one hour for this meeting, to involve the whole practice because consensus building and agreement on next steps will require input from all practice staff. If one GP or nurse cannot make the meeting, the pharmacist will meet with each separately to cover the main educational points and practical decisions.

The objective is to gain firm commitment (from all individuals and the entire practice as a unit) on whether, when and how to phase a plan for systematic implementation of the guideline. Having thoroughly considered each part of the implementation plan in terms of workload implications, the pharmacist will deliver an interactive presentation including:

- The evidence base;
- A guideline summary;
- Anonymised patient specific prescribing and clinical data;
- Simulation of therapeutic challenges;
- A comparison of the practice’s prescribing trends with neighbouring practices;
- Opinion leaders’ answers to questions;
- Possible targets.

In the plan for change, the pharmacist will describe timelines; targets (anticipated improvements in prescribing, including tentative quantification of improved clinical outcomes for patients) and the benefits. Lower prescribing costs resulting from the changes will be anticipated and quantified. The pharmacist will offer help in steering, monitoring and feeding back progress on behalf of the practice.

All pharmacists will ensure their presentation adheres to the following points:

1. Agree clear educational objectives;

2. Agree clear behavioural objectives;

3. Present both sides of controversial issues;

4. Stimulate active participation;

5. Use concise graphics;

6. Highlight and repeat essential messages;

7. Obtain agreement and build on it;

8. Be enthusiastic;

9. Keep to allotted time.

**4.1.6 Between the second and third meetings**

Following the second meeting, pharmacists will use their time (one day per week), to implement change, reinforce and feedback through progress reports, until the agreed actions are carried out.

The pharmacists will record changes in prescribing at patient level. These data will be summarised and used in the presentation for the third meeting.

**4.1.7 Meeting 3**

The objectives of meeting three are to:

- Re-iterate key points agreed during meeting two;
- Enable two way feedback, consolidate and reinforce changes to prescribing;
- Address any ongoing implementation difficulties.

Pharmacists will try to meet one to one (face to face) with practice staff, at a time convenient to the GP or nurse. Pharmacists will be encouraged to provide feedback on any prescribing changes made in line with the agreement reached during meeting two. They will be asked to present statin prescribing of other (anonymised) practices over the same time period for comparison purposes. Reinforcement and repetition of key educational messages, reminders of agreed actions and open discussion of progress in relation to timescales will form key components of meeting three.

All pharmacists will leave meeting three with a firm commitment from the GPs and nurses to continue prescribing simvastatin 40mg for eligible patients.

**4.2 Usual Care**

Practices randomised into UC will receive no pharmacist-led prescribing support throughout the study period (intervention and follow up).

**5. Appendices**

**Appendix I**

## Heart Protection Study – Subgroups for 1^st^ vascular event (CHD / CVA / revascularisation)

## Category N Placebo Simvastatin ARR NNT

Coronary Heart Disease 13386 27.5% 21.8% 5.7% 18

Any PVD 6748 32.7% 26.4% 6.3% 16

PVD alone 2701 30.5% 24.7% 5.8% 17

CVA (any stroke) 3280 29.8% 24.7% 5.1% 20

CVA (stroke alone) 1820 23.6% 18.7% 4.9% 20

Diabetes Mellitus (any diabetes) 5963 25.1% 20.2% 4.9% 20

Diabetes Mellitus (diabetes alone) 3982 18.6% 13.8% 4.8% 21

Total Cholesterol <5 4072 23.1% 17.7% 5.4% 19

Total Cholesterol ≥5 but <6 7883 24.5% 18.9% 5.6% 18

Total Cholesterol ≥6 8581 26.8% 21.6% 5.2% 19

ARR=absolute risk reduction; NNT=number needed to treat for 5 years to prevent one event

**Appendix II**

**
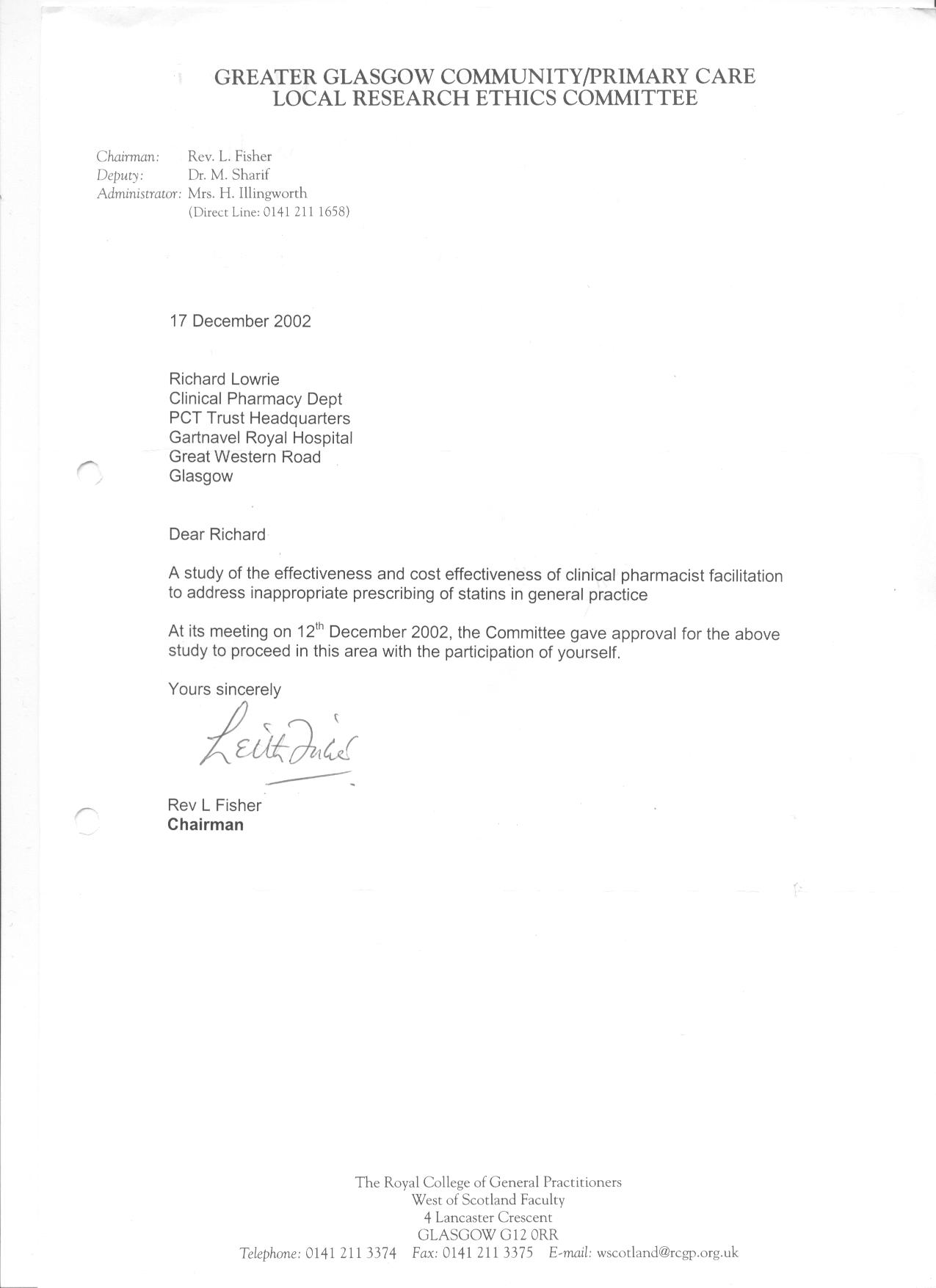
**

## Appendix III

## Secondary prevention of coronary heart disease and stroke

**Cholesterol**

##### Patients with established vascular disease are at high risk and should be treated with a statin regardless of total blood cholesterol concentration

##### i.e. Previous MI / pre- or post-CABG / pre- or post-Angioplasty / Angina / Angiographic coronary artery disease / Ischaemic stroke or TIA / Peripheral Arterial Disease / Diabetic patients aged ≥ 40 years

† *Discuss with lipid clinic if in doubt*

**Do within 24 hours of onset of acute MI*

**Random non-fasting test for total cholesterol*** **and LFT’s**

Consider secondary causes & familial hyperlipidaemia if Cholesterol >8.0 mmol/L^†^

***See BNF for cautions, contra-indications and clinically important interactions.***

# Re-test at 1 month

Random non-fasting total cholesterol + triglycerides + LFT’s

# Goals of Treatment by Three Months

# Total cholesterol concentration <5.00 mmol/l

# (<4.00 mmol/L for post CABG patients)

# and

# Reduce cholesterol concentration by ≥ 25%

Cholesterol Goals Achieved

Annual review to ensure continued concordance.

**Treat all patients with statin regardless of baseline cholesterol concentration**

Recommended drug & daily dose

Simvastatin 40mg daily

Consider **Atorvastatin 80mg** in acute coronary syndrome with elevated troponin

Cholesterol goals not achieved

Discuss concordance. Switch to Atorvastatin 40mg, and then if necessary increase to 80mg. Consider rosuvastatin 20mg if goals still not met. The use of other lipid-lowering agents is not recommended without specialist advice.

Triglycerides > 4.0mmol/L

Check fasting sample^‡^

**^‡^ *Consider referral to lipid clinic in resistant cases, if liver transaminases > 2x normal, or if statin not tolerated.***

## Atherosclerotic arterial disease is of multifactorial origin. No single risk factor, including cholesterol concentration, should be viewed in isolation.

- Encourage smoking cessation (consider nicotine replacement therapy)
- All other risk factors hypertension, diabetic control, should be addressed (see separate guidelines)
- Dispersible aspirin 75mg (not enteric coated) should be taken by all those with occlusive arterial disease in the absence of contraindications (active peptic ulceration, a bleeding disorder or true hypersensitivity) – see antiplatelet guideline
- Consider treatment with ACE-inhibitors especially in patients with left ventricular dysfunction or heart failure
- Consider β-blockers for those with CHD – ensure they are on the CHD register and seen for annual CDM review
- Dietary and other lifestyle advice e.g. alcohol, obesity, physical activity, should be given.

**Appendix IV**

**Clinical Facilitation**^§^**: a summary for practices**

This is a multifaceted intervention, rooted in the belief that more intensive efforts to alter prescribing practice are generally more successful. Clinical Facilitation involves prescribing support pharmacists meeting individually with GPs or nurses to identify any obstacles standing in the way of offering patients statins to lower cholesterol and then providing specific, practical solutions (facilitation) to overcome these obstacles. Some techniques used to change prescribing include: implementation of the evidence may include a practice based clinical audit; open, honest communication; agreement on help needed by the practice to improve patient attendance. Increasing prescribing of simvastatin 40mg for patients with vascular disease will feature in each meeting.

**Pre – meeting 1**

Prior to meeting 1, the pharmacist searches the practice computer to ascertain numbers of patients with confirmed vascular disease who are not treated with statins in line with the local guideline.

**Meeting 1 (up to 30 minutes)**

Outline of process. Identification of GP/nurse learning needs (knowledge based or organisational) and identification of each practitioner’s preferred learning method. The information gathered pre meeting 1 is summarised, displayed graphically and shared with the GP/nurse during the first meeting. If expert opinion is requested to address uncertainties in therapeutic management, this is sought and incorporated into the second meeting. Discussion of need and methods to address the gap between guideline and implementation.

Key outcome: Agreement on individual information /practice organisational barriers that prevent the identified patients with vascular disease being identified and offered statins. Targets for changes in prescribing are discussed and agreed if possible.

**Meeting 2 (up to 1 hour)**

Interactive, individualised presentation by pharmacist facilitator. Delivery of therapeutic information about the practice’s prescribing of statins and summarised trial information in a concise, unbiased, understandable format. The pharmacist suggests and seeks agreement for solutions to questions/barriers raised at 1^st^ meeting. Anonymised patient specific prescribing and clinical data are used to simulate therapeutic challenges. The pharmacist will offer the views of respected peers/specialists.

Key outcome: Commitment by the GP/nurse to changing their approach to prescribing statins for patients identified pre meeting 1, with vascular disease, with or without a statin. Responsibilities for behavioural change and timelines for action agreed. If agreed action involves the pharmacist committing to ongoing support for creating a register of patients with vascular disease, the pharmacist agrees when this can be done. Pharmacist will then facilitate change by visiting the practice weekly until the agreed actions are carried out. The actions may involve the identification of suitable patients followed by mailing of letters to patients explaining benefits of statins and requesting the patient’s attendance at clinic for a review.

**Meeting 3 (up to 30 minutes)**

Positive reinforcement of changes made since meeting 2. The pharmacist searches the practice computer system to identify a sample of patients with coronary heart disease to assess the impact of the facilitation process. Key messages are repeated. Additional barriers to change management are identified and addressed. Graphical presentation of how the practice’s prescribing statistics have changed is fed back.

Key outcome: Practice agree ongoing, systematic approach to optimising statin use in line with guideline

^§^ Statin Outreach Support, SOS

**6. References**

Allard, J. Herbert, R. Rioux, M. Asselin, J. and Voyer, L. (2001). Efficacy of clinical medication review on the number of potentially inappropriate prescriptions prescribed for community dwelling elderly people. *CMAJ*. 164 (9) p1291-6.

Allery, LA. Owen, P. and Robling MR. (1997). Why General Practitioners and consultants change their clinical practice: a critical incident study. *BMJ*. 314 (7084) p870-4.

Anderson, JF. McEwan, KL. and Hudley, WP. (1996). Effectiveness of notification and group education in modifying prescribing of regulated analgesics. *CMAJ*. 154 (1) p31-39.

Armstrong, D. Reyburn, H. and Jones R. (1996). A study of general practitioners’ reasons for changing their prescribing behaviour. *BMJ*. 312. p949-952.

Aronson, JK. (2006). Prescribing statins. *Br J Pharmacol*. 61. p502-512.

Aspire Steering Group. Bowker, TJ. Clayton, TC. and Ingham, J. et al. (1996). A British Cardiac Society survey of the potential for the secondary prevention of coronary heart disease: ASPIRE (action on secondary prevention through intervention to reduce events) principal results. *Heart*. 75. p334-42.

Asworth, M. Golding, S. and Majeed, A. (2002). Prescribing indicators and their use by primary care groups to influence prescribing. *J Clin Pharm Ther*. 27 (3) p197-204.

Ashworth M, Lloyd D, Smith RS, Wagner A, Rowlands G. (2006). Social deprivation and statin prescribing: a cross sectional analysis using data from the new UK general practitioner ‘Quality and Outcomes Framework’. *J Pub Health 29:40-47*

Audit Commission. (1994). A prescription for improvement: towards more rationale prescribing in general practice. *London HMSO*.

Avery, A. Walker, B. and Heron, T. (1997). Do prescribing formularies help GPs prescribe from a narrower range of drugs? A controlled trial of the introduction of prescribing formularies for NSAIDs. *Br J Gen Prac*. 47. p810-4.

Avorn, J. and Soumerai, SB. (1983). Improving drug therapy decisions through educational outreach. A randomized controlled trial of academically based “detailing”. *N Eng J Med*. 308. p1457-1463.

Baker, R. Reddish, S. Robertson, N. Hearnshaw, H. and Jones, B. (2001). Randomised controlled trial of tailored strategies to implement guidelines for the management of patients with depression in general practice. *Br J Gen Pract*. 51. p737-741.

Barber, N. (1995). What constitutes good prescribing? *BMJ*. 310. p923-925.

Beaudry, JS. (1989). The effectiveness of continuing medical education: a quantitative synthesis. *Journal of Continuing Education in the Health Professions.* 9. p285-307.

Beilby, JJ. And Silagy, CA. (1997). Trials of providing costing information to general practitioners: a systematic review. *Med J Aust*. 167 (2) p89-92.

Benedetto, S. Sloan, A. and Duncan, B. (2000). Impact of interventions designed to increase market share and prescribing of fexofenadine at HMOs. *Am J Health Syst Pharm*. 57 (19) p1778-1785.

Beney, J. Bero, LA. and Bond, C. (2000). Expanding the roles of outpatient pharmacists: effects on health services utilisation, costs and patient outcomes. *Cochrane Database of Systematic Reviews*. (3).

Bernal-Delgado, E. Galeote-Mayor, M. Pradas-Arnal, F. and Peiro-Moreno, S. (2002). Evidence based educational outreach visits: effects on prescriptions of non-steroidal anti-inflammatory drugs. *Journal of Epidemiology and Community Health.* 56 (9) p653-8.

Bero, LA. Grilli, R. Grimshaw, JM. Harvey, E. Oxman, AD. and Thomson, MA. (1998). Closing the gap between research and practice: an overview of systematic reviews of interventions to promote the implementation of research findings. *BMJ*. 317. p465-8.

Bloom, BS. and Jacobs, J. (1985). Cost effects of restricting cost effective therapy. *Med Care*. 23. p872-80.

Boardman, HF, and Thomson, V. (1999). Views of general practitioners and nursing home staff about an educational outreach programme. *Pharm J*. 263. R44.

Bodgen, P. Koontz, L. Williamson, P. and Abbot, R. (1997). The physician and pharmacist team: an effective approach to cholesterol reduction. *JGIM*. 12 (3) p158-164.

Bradley, CP. (1991). Decision making and prescribing patterns – a literature review. *Fam Pract*. 8. p276-285.

Bradley, CP. (1992). Factors which influence the decision whether or not to prescribe: the dilemma facing general practitioners. *Br J Gen Pract*. 42. p454-458.

Bradshaw, N. Fone, DL. and Walker, R. (1999). Explaining variation in statin prescribing; a practice based analysis. *Pharm J.* 263. R19.

British Cardiac Society. British Hyperlipidaemia Association. British Hypertension Society. and endorsed by the British Diabetic Association. (1998). Joint British recommendations on prevention of coronary heart disease in clinical practice. *Heart.* 80 (suppl 2) S1-29.

Britten, N. (1995). Patients’ demands for prescriptions in primary care. *BMJ*. 310. p1084-1085.

Britten, N. and Ukoumunne, O. (1997). The influence of patients’ hopes of receiving a prescription on doctors’ perceptions and the decision to prescribe: a questionnaire survey. *BMJ*. 315. p1506-1510.

Brewer, D. (1998). The effect of drug sampling policies on residents’ prescribing. *Family Medicine.* 30 (7) p482-6.

Brook, RH. McGlynn, EA. and Clearly, PD. (1996). Quality of Health Care, part 2: measuring quality of care. *N Eng J Med*. 335. p966-969.

Brown, CA. Belfield, CR. and Field, SJ. (2002). Cost effectiveness of continuing professional development in health care: a critical review of the evidence. *BMJ*. 324. p652-655.

Brown, J. Shye, D. and McFarland, BH. (2000). Controlled trials of CQI and academic detailing to implement a clinical practice guideline for depression. *Jt Comm J Qual Improv*. 26. p39-54.

Brufsky, J. Ross-Degnan, D. Calabrese, D. Gao, X. and Soumerai, S. (1998). Shifting physician prescribing to a preferred Histamin-2-receptor antagonist: effects of a multifactorial intervention in a mixed model health maintenance organisation. *Med Care*. 36 (3) p321-332.

Bull, N. Williams, J. Nicholls, P. and Lawrenson, RA. (2003). Increased statin prescribing in patients with diabetes after the introduction of NSF for Coronary Heart Disease. *Pract Diabet International*. 20 (9) p313-7.

Buntix, F. Wiunkens, R. Grol, R. and Knottnerus, JA. (1993). Influencing diagnostic and preventive performance in ambulatory care by feedback and reminders: a review. *Fam Pract*. 10. p219-228.

Burgers, J. Grol, R. Zaat, J. Spies, TH. Van Der Bij, A. and Mokkink, H. (2003). Characteristics of effective clinical guidelines for general practice. *Br J Gen Pract*. 53. p15-19.

Burrel, CJ. Skehan, JD. Cowley, ML. Barrett, CW. and Mills, PG. (1990). Districts use of thrombolytic agents. *BMJ*. 300. P237-8.

Calvo, CB. and Rubenstein, A. (2002). Influence of new evidence on prescription patterns. *Journal of the American Board of Family Practice*. 15 (6) p457-62.

Campbell, NC. Thain, J. Deans, HG. Ritchie, LD. and Rawles, JM. (1998). Secondary prevention in coronary heart disease: a baseline survey of provision and possibility in general practice. *BMJ*. 316. p1430-4.

Capewell, S. Morrison, CE. and McMurray, JJV. (1999). Contribution of modern cardiovascular treatment and risk factor changes to the decline in coronary heart disease mortality in Scotland between 1975 and 1994. *Heart*. 81. p380-386.

Carthy, P. Harvey, I. Brawn, R. and Watkins, C. (2000). A study of factors associated with cost and variation in prescribing among GPs. *Fam Pract*. 17 (1) p36-41.

Clinical Resource and Audit Group. (1993). Clinical Guidelines. *Edinburgh: Scottish Office*.

Clinical Quality Improvement Network Investigators. (1995). Low incidence of assessment and modification of risk factors in acute care patients at high risk for cardiovascular events, particularly amongst females and the elderly. *Am J Cardiol*. 76. p570-573.

Cockburn, J. and Pit, S. (1997). Prescribing behaviour in clinical practice: expectations and doctors’ perceptions of patients’ expectations – a questionnaire study. *BMJ*. 315. p520-523.

Cooper, H. Carlisle, C. Gibbs, T. and Watkins, C. (2001). Developing an evidence base for interdisciplinary learning: a systematic review. *Journal of Advanced Nursing*. 35 (2) p228-237.

Crotty, M. Whitehead, C. Rowett, D. Halbert, J. Weller, D. Finucane, P. and Esterman, A. (2004). An outreach intervention to implement evidence based practice in residential care: a randomised controlled trial. *BMC Health Services Research*. 4 (1) p6.

Cupples, M. and McKnight, A. (1994). A randomised controlled trial of health promotion in general practice for patients at high cardiovascular risk. *BMJ*. 309. p993-6.

David, D. Evans, M. and Jadad, A. (2003). The case for knowledge translation: shortening the journey from knowledge to effect. *BMJ*. 327. p33-5.

Davis, D. (1998). Does CME work? An analysis of the effect of educational activities on physician performance or health care outcomes. *Int J Psychiatry Med*. 28. p21-39.

Davis, DA. Thomson, MA. Oxman, AD. and Haynes, RB. (1995). Changing physician performance: a systematic review of continuing medical education strategies. *JAMA*. 274 (9) p700-705.

Delamothe, T. (1999). Wanted: guidelines that doctors will follow. *BMJ*. 307. p218-60.

Denig, P. Jaaijer-Ruskamp, FM. and Zijslign, DH. (1990). Impact of a drug bulletin on the knowledge, perception of drug utility and prescribing behaviour of physicians. *Ann Pharmacother*. 24 (1) p87-93.

Department of Health (2004). Chronic disease management. A compendium of information. London: Department of Health. *London: HMSO*.

Department of Health and NHS Institute for innovation and improvement. (2007). Better Care Better Value indicators.

De Santis, G. Harvey, KJ. Howard, D. Mashford, ML. and Moulds, RF. (1994). Improving the quality of antibiotic prescription patterns in general practice. The role of educational intervention. *Med J Aust*. 160 (8) p502-5.

De Wilde, S. Carey, IM. Bremnder, SA. Richards, N. Hilton, SR. and Cook DG. (2003). Evolution of statin prescribing 1994-2001: a case for ageism but not sexism. *Heart*. 89. p417-21.

Divine, GW. Brown, JT. and Frazier, LM. (1992). The unit of analysis error in studies about physicians’ patient care behaviour. *J Gen Intern Med*. 7. p623-9.

Diwan VK, Wahlstrom R, Tomson G, Beermann B, Sterky G, Eriksson Bo. (1995). Effects of “Group detailing” on the prescribing of lipid lowering drugs: a randomized controlled trial in Swedish primary care. *J Clin Epidemiol.* 48(5):705-711.

Dollan Mullen, P & Green, L. and Persinger D. (1985). Clinical trials of patient education for chronic conditions: a comparative meta-analysis of intervention types. *Preventive medicine*. 14. p753-781.

Donner, A. Birkett, N. and Buck, C. (1981). Randomisation by cluster: sample size requirements and analysis. *AM J Epidemiol*. 114. p906-914.

Donner, A. Brown, KS. and Brasher, P. (1990). A methodological review of non therapeutic intervention trials employing cluster randomisation, 1979-1989. *Int J Epidemiol*. 19. p795-800.

Donner, A. and Klar, N. (2000). Design and analysis of Cluster Randomisation trials in health research. *Arnold. London*.

Downs, JR. Clearfield, M. Weis, S. and Whitney, E. et al. (1998). Primary prevention of acute coronary events with lovastatin in men and women with average cholesterol levels (AFCAPS/TexCAPS). *JAMA*. 279. p1615-22.

Doyne, EO. Alfaro, MP. Siegel, RM. Atherton, HD. Schoettker, PJ. Bernier, J. and Kotagal UR. (2004). A randomised controlled trial to change antibiotic prescribing patterns in a community. *Archives of Paediatric and Adolescent Medicine*. 158 (6) p577-83.

EUROASPIRE I and II group. (2001). Clinical reality of coronary prevention guidelines: a comparison of EUROASPIRE I and II in nine countries. *Lancet*. 357. p995-1001.

EUROASPIRE II study group. (2001). Lifestyle and risk factor management and use of drug therapies in coronary patients from 15 countries. Principal results from EUROASPIRE II study group. *European Heart Journal*. 22. p554-77.

Evans, A. Tolonen, H. Hense, HW. Ferrario, M. Sans, S. and Kuulasmaaa, K. (2001). WHO MONICA project. Trends in coronary risk factors in the WHO MONICA project. *Int J Epidemiol*. 30 (Supp 1) S35-s40.

Fairhurst, K. and Huby, G. (1998). From trial data to practical knowledge: qualitative study of how general practitioners have accessed and used evidence about statin drugs in their management of hypercholesterolaemia. *BMJ*. 317. p1130-1134.

Feder, G. Griffiths, C. Highton, C. Eldridge, S. Spence, M. and Southgate, L. (1995). Do clinical guidelines introduced with practice based education improve care of asthmatic and diabetic patients? A randomised controlled trial in general practices in east London. *BMJ*. 311. p1473-8.

Feder, G. Griffiths, C. Eldridge, S. and Spence, M. (1999). Effect of postal prompts to patients and general practitioners on the quality of primary care after a coronary event (POST): randomised controlled trial. *BMJ*. 318. p1522-6.

Fender, G. Prentice, A. Gorst, T. Nixon, R. Duffy, W. Day, N. and Smith, S. (1999). Randomised controlled trial of educational package on management of menorrhagia in primary care: the Anglia menorrhagia education study. *BMJ*. 318. p1246-1250.

Fernie, JA. Penning-Van, B. Termorshizen, F. Goettsch, WG. Klungel, OH. Kastelein, JJP. And Herings, RMC. (2006). Adherence to evidence based statin guidelines reduces the risk of hospitalisations for acute myocardial infarction by 40%: a cohort study. *Eur Heart J*. 28 (2) p154-159. doi:10.1093/eurheartj/ehl391.

Field, J. (1989). How do doctors and patients react to the introduction of a practice formulary?. *Fam Pract*. 6. p135-140.

Figueiras, A. Sastre, I. and Gestal-Otero, JJ. (2001). Effectiveness of educational interventions on the improvement of drug prescription in primary care: a critical literature review. *Journal of Evaluation in Clinical Practice*. 7 (2) p223-241.

Finklelstein, JA. Davis, RL. Dowell, SF. Metlay, JP. Soumerai, SB. Rifas-Shiman, SL. Higham, M. and Miller, Z. (2001). Reducing antibiotic use in children: a randomised trial in 12 practices. *Paediatrics*. 108 (1) p1-7.

Fisk, D. Rakfeldt, J. and McCormack, E. (2006). Assertive outreach: an effective strategy for engaging homeless persons with substance disorders into treatment. *Am J Drug Alcohol Abuse*. 32 (3) p479-486.

Foy, R. Eccles, M. and Grimshaw, G. (2001). Why does primary care need more implementation research?. *Family Practice*. 18. p353-355.

Fraser, SW. and Plesk, P. (2003). Translating evidence into practice: a process of externally driven spread or personal adoption?. *Education for Primary Care*. 14. p129-38.

Freeman, AC. and Sweeney, K. (2001). Why general practitioners do not implement evidence: qualitative study. *BMJ*. 323. p1100-1112.

Freemantle, N. Eccles, M. and Wood, J. et al. (1999). A randomised trial of evidence based outreach (EBOR): rationale and design. *Controlled Clinical Trials*. 20. p479-492.

Freemantle, M. Harvey, EL. Wolf, F. Grimshaw, JM. Grilli, R. and Belo LA. (2005). Printed educational materials: effects on professional practice and healthcare outcomes (Cochrane Review). *The Cochrane library*. (3) Oxford: Update Software; 2005.

Fretheim, A. Oxman, AD. Treweek, S. and Bjorndal, A. (2003). Rational Prescribing in Primary Care. A randomised trial of a tailored intervention to improve prescribing of antihypertensive and cholesterol lowering drugs in general practice. *BMC Health Services Research*. 3 (1) p5.

Frolkis, JP. (1998). Physician noncompliance with the 1993 National Cholesterol Education Program Guidelines. *Circulation*. 98. p851-5.

Gask, L. Dorwick, C. Dixon, C. Sutton, C. Perry, R. Togerson, D. and Usherwood, T. (2004) A pragmatic cluster randomised controlled trial of an educational intervention for GPs in the assessment and management of depression. *Psychological Medicine*. 34 (1) p63-72.

Getting evidence into practice. (1998). *Effectiveness Matters*

Gibson, A. Asthana, S. Brigham, P. Moon, G. and Dicker, J. (2002). Geographies of need and the new NHS: methodological issues in the definition and measurement of the health needs of local populations. *Health and Place*. 8. p47-60.

Gilley, J. (1994). Towards rational prescribing. *BMJ*. 308. p731-2.

Glare, J. (2006). Involvement in clinical trials can modify GP prescribing patterns. *JAMA*. 295. p2759-64.

Goldberg, HI. Wagner, EH. Fihn, SD. Martin, DP. and Horowitz CR. (1998). A randomised controlled trial of CQI teams and academic detailing: can they alter compliance with guidelines? *The Joint Commission Journal on Quality Improvement*. 24 (3) p130-42.

Gonzales, R. Steiner, JF. Lum, A. and Barrett, PH. (1999). Decreasing antibiotic use in ambulatory practice: impact of a multidimensional intervention on the treatment of uncomplicated acute bronchitis in adults. *JAMA*. 281 (16) p1512-9.

Gosden, T. Forland, F. Kristiansen, IS. Sutton, M. Leeses, B. and Giuffrida, A. (2005). Capitation, salary, fee for service and mixed systems of payment: effects on the behaviour of primary care physicians. *The Cochrane Library*. (2).

Grant, GB. Gregory, DA. and Van Zwanenberg TD. (1985). Development of a limited formulary for general practice. *Lancet*. 1. p1030-1032.

Green, PE. (1985). The general practice formulary – its role in rational therapeutics. *J R Coll Gen Pract*. 35. p570-572.

Greenfield, PR. (1982). Report to the secretary of state for social services of the informal working group on effective prescribing. *London: HMSO*.

Grimshaw, JM. Thomas, RE. MacLennan, G. Fraser, C. Ramsay, CR. and Vale, L. (2004). Effectiveness and efficiency of guideline dissemination and implementation strategies. *Health Technol Assess*. 8 (6).

Grol, R. (1992). Implementing guidelines in general practice care. *Quality in Health Care*. 1. p184-191.

Grol, R. (1997). Beliefs and evidence in changing clinical practice. *BMJ*. 315. p418-21.

Grol, R. Dalhuisjsen, J. Thomas, S. Veld, C. Rutten, G. and Mokkink, H. (1998). Attributes of clinical guidelines in general practice: observational study. *BMJ*. 317. p858-861.

Haaijer-Ruskamp, FM. and Denig, P. (1995). Impact of feedback and peer review on prescribing. *J Royal Coll Gen Practitioners.* Occasional paper 70. p13-19.

Habraken, H. Janssens, I. Soenen, K. Lannoy, J. and Bogaert, M. (2003). Pilot study on the feasibility and acceptability of academic detailing in general practice. *Eur J Clin Pharmacol.* 59 (3) p253-60.

Hall, J. Radley, A. and John, S. (1995). Community and hospital pharmacist co-operation in an outreach anticoagulant clinic. *Pharm J*. 255 (suppl) R24.

Harding, JM. Modell, M. and Freudenberg, S. (1985). Prescribing: the power to set limits. *BMJ*. 290. p450-453.

Harris, RH. MacKenzie, TD. Leeman-Castillo, B. Corbett, KK. Batal, HA. Maselli, JH. and Gonzales, R. (2003). Optimising antibiotic prescribing for acute respiratory tract infections in an urban urgent care clinic. *Journal of General Internal Medicine.* 18 (5) p326-334.

Hassey, A. Gerrett, D. and Wilson, A. (2001). A survey of validity and utility of electronic patient records in a general practice. *BMJ*. 322. p1401-5.

Hatoum, HT. and Akhras, K. (1993). 1993 Bibliography: a 32 year literature review on the value and acceptance of ambulatory care provided by pharmacists. *Ann Pharmacother*. 27. p1106-19.

Haynes, B. (1998). Barriers and bridges to evidence based clinical practice. *BMJ.* 17. p273-276.

Haynes, B. Guyatt, G. and Devereaux, P. (2002). Clinical expertise in the era of evidence based medicine and patient choice. *ACP Journal Club.* 136. A11-14.

Hemminiki, E. (1975). Review of literature on factors affecting drug prescribing. *Soc Sci Med*. 9. p111-6.

Hill-Smith, I. (1996). Sharing resources to create a district drug formulary: a countrywide controlled trial. *Br J Gen Pract.* 46. p271-275.

Hoereger, TJ. (1998). Treatment patterns and distribution of low density lipoprotein cholesterol levels in treatment eligible United States adults. *Am J Cardiol.* 82. p61-5.

HPS. Heart Protection Study Collaborative Group. (2002). MRC/BHF Heart Protection Study of cholesterol lowering with simvastatin in 20,536 high risk individuals: a randomised placebo controlled trial. *Lancet.* 360. p7-22.

Hulscher, ME. Van Drenth, BB. Mokkink, HG. Van De Lisdonk, EH. Van Wouden, JC. Van Weel, C. and Grol, RP. (1998). Tailored outreach visits as a method for implementing guidelines and improving preventive care. *Int J Qual Health Care.* 10 (2) p105-12.

Hunskaar, S. Hannestad, YS. Backe, B. and Matheson, I. (1996). Direct mailing of consensus recommendations did not alter GP’s knowledge and prescription of oestrogen in the menopause. *Scandanavian Journal of Primary Health Care.* 14 (4) p203-8.

Hux, JE. Melady, MP. and De Boer, D. (1999). Confidential prescriber feedback and education to improve antibiotic use in primary care: a controlled trial. *CMAJ*. 161 (4) p388-92.

Ilett, KF. Johnson, S. Greenhill, G. Mullen, L. Brockis, J. and Golledge, Reid DB. (2000). Modification of general practitioner prescribing of antibiotics by use of a therapeutics adviser (academic detailer). *Br J Clin Pharmacol.* 49 (2) p168-73.

Anon. Implementing clinical guidelines: can guidelines be used to improve clinical practice? (1994). *Effective Health Care*. 8.

Institute of Medicine. (1990). Clinical Practice Guidelines: Directions for a New Program. *Washington: National Academy Press.*

Jamtvelt, G. Young, JM. Kristoffersen, DT. Thomson, MA. and Oxman, AD. (2005). Audit and feedback. *Cochrane Database of Systematic Reviews.* 3.

Jick, H. Jick, S. and Derby, L. (1991). Validation of information on general practitioner based computerised data resource in the United Kingdom. *BMJ*. 302. p766-768a.

Jick, H. Terris, BZ. Derby, L. and Jick, SS. (1992). Further validation of information recorded on general practitioner based computerised data resource in the United Kingdom. *Pharmacoepidimiol Drug Safety.* 1. p347-349b.

Jolles, M. (1981). Why not compile your own formulary? *J R Coll Gen Pract.* 31. p372.

Jolly, K. Bradley, F. Sharp, S. Smith, H. Thomson, S. and Kinmouth, AL. et al. (1999). Randomised controlled trial of follow up care in general practice of patients with myocardial infarction and angina: final results of the Southampton heart integrated care project (SHIP). *BMJ*. 318. p706-11.

Kempner, N. (1996). “GP pharmacist” what do they do? *Pharm J*. 256 p196-7.

Kreling, DH. Knocke, DJ. and Hammel, RW. (1989). The effects of an internal analgesic formulary restriction on Medicaid drug expenditures in Wisconsin. *Med Care*. 27. p34-44.

Langham, J. Tucker, H. Sloan, D. Pettifer, J. Thom, S. and Hemmingway, H. (2002). Secondary prevention of cardiovascular disease: a randomised trial of training in information management, evidence based medicine, both or neither: the PIER trial. *Br J Gen Pract*. 52. p818-824.

Latour Perez, J. Diaz Mandejar, R. De La, Vega. and Ortega, AI. et al. (2000). Failure of an intervention for changing hypo-cholesterolaemic drugs prescription in primary care. *Atencion Primaria.* 26 (4) p245-248.

Leach, R. (1999). Options for selecting PCG prescribing advice. *Prescriber.* Feb. p34-39.

Lexchin, J. (1993). Interactions between physicians and the pharmaceutical industry: what does the literature say? *CMAJ.* 149 (10) p1401-7.

LIPID study group. (1998). The long term intervention with pravastatin in ischaemic disease. Prevention of cardiovascular events and death with pravastatin in patients with coronary heart disease and a broad range of initial cholesterol levels. *N Eng J Med.* 339. p1349-57.

Lipton, HL. Bero, LA. Bird, JA. and McPhee, S. (1992). The impact of clinical pharmacists’ consultation on physician’s geriatric drug prescribing. A randomised controlled trial. *Med Care.* 30. p646-58.

Lipton, HL. Byrns, PJ. Soumerai, SB. and Chrischilles, EA. (1995). Pharmacists as agents of change for rational drug therapy. *Int J Tech Assessment in Health Care.* 11 (3) p485-508.

Mackay DF, Watt GCM. (2010). General Practice size determines participation in optional activities: cross sectional analysis of a national primary care system. *Primary Health Care Research and Development.* 11:271-279.

MacLennan, GS. Ramsay, CR. Mollison, J. Campbell, MK. Grimshaw, JM. and Thomas, RE. (2003). Room for improvement in the reporting of cluster randomised controlled trials in behavioural change research. *Control Clin Trials.* 24 p69-70S.

Madridejos-Mora, R. Amado-Guirado, E. and Perez-Rodriguez, MT. (2004). Effectiveness of the combination of feedback and educational recommendations for improving drug prescribing in general practice. *Medical Care.* 42 (7) p643-8.

Majeed, A. Moser, K. and Maxwell, R. (2000). Age, sex and practice variations in the use of statins in general practice in England and Wales. *J Pub Health Med.* 22. p275-279.

Mair, F. Crowley, T. and Bundred, P. (1996). Prevalence, aetiology and management of heart failure in general practice. *Br J Gen Pract*. 46. p77-79.

Majeed, A. (2000). Age, sex and practice variations in the use of statins in general practice in England and Wales. *J pub Health Med*. 22. p275-9.

Marinker, M. and Reilly, P. Rational prescribing. How can it be judged?

Mariniker, M. (1994). Controversies in Health Care policies: challenges to practice. *London: BMJ publishing.*

Mashru, M. and Lant, A. (1997). Interpractice audit of diagnosis and management of hypertension in primary care: educational intervention and review of medical records. *BMJ.* 314. p942-946.

Mason, J. Freemantle, N. Nazareth, I. Eccles, M. Haines, A. and Drummond, M. (2001). When is it cost effective to change the behavior of health professionals? *JAMA.* 286 (23) p2988-2992.

May FW, Rowett DS, Gilbert AL, McNeece JI, Hurley E. Outcomes of an educational outreach service for community medical practitioners: non steroidal anti inflammatory drugs. Med J Aust 1999; 170(10): 471-4.

McCartney, P. Macdowall, W. and Thorogood, M. (1997). A randomised controlled trial of feedback to general practitioners of their prophylactic aspirin prescribing *BMJ.* 315. p35-6.

McColl, A. Smith, H. White, P. and Field, J. (1998). General practitioners’ perceptions of the route to evidence based medicines: a questionnaire survey. *BMJ.* 316. p361-365.

McCormick, D. Gurwitz, JH. and Lessard, D. et al. (1999). Use of aspirin, B-Blockers and lipid lowering medications before recurrent acute myocardial infarction: missed opportunities for prevention? *Arch Intern Med.* 159. p561-567.

McGavock, H. Webb, CH. Johnson, GD and Milligan, E. (1993). Market penetration of new drugs in one UK region: implications for GPs and administrators. *BMJ.* 307. p1118-1120.

McGavock, H. Wilson Davis, K. and Connoly, JP. (1999). Repeat prescribing management – a cause for concern? *Br J Gen Pract.* 49. p343-7.

McLaughlin, PJ. and Donaldson, JF. (1991). Evaluation of continuing medical education programmes: a selected literature 1984-1988. *Journal of Continuing Education in the Health Professions.* 11. p65-84.

McMullin, ST. Hennenfent, JA. Ritchie, DJ. Huey, WY. and Lonergan, TP. (1999). A prospective, randomised trial to assess the cost impact of pharmacist initiated interventions. *Arch Intern Med.* 159. p2306-9.

Medical Research Council. (2000). A framework for the development and evaluation of RCTs for complex interventions to improve health. *London: MRC*

Medical Research Council. (2011). Developing and evaluating complex interventions: new guidance. A framework for the development and evaluation of RCTs for complex interventions to improve health. *London: MRC.* [*www.mrc.ac.uk/complexinterventionsguidance*](http://www.mrc.ac.uk/complexinterventionsguidance) *Last accessed June 2011.*

Minhas, R. (2004). Statins in primary care: bridging the treatment gap. *Br J Cardiol*. 11 (6) p487-91.

Missouris, CG. (2001). Coronary Heart Disease in the statin and aspirin era: are results of clinical trials being put into practice? *Eur J Intern Med.* 12. p490-5.

Moher, M. Yudkin, P. Turner, R. Schofield, T. and Mant, D. (2000). An assessment of morbidity registers for coronary heart disease in primary care. *Br J Gen Pract.* 50. p706-709.

Moher, M. Yudkin, P. Wright, L. and Turner, R. et al. (2001). Cluster randomised controlled trial to compare three methods of promoting secondary prevention of coronary heart disease in primary care. *BMJ.* 322. p1-7.

Moon J, Bogle R. (2006). Switching statins could save the NHS £2 billion over 5 years. *BMJ*. 332. p1344-1345.

Morrison, A. and Wertheimer, AI. (2001). Evaluation of studies investigating the effectiveness of pharmacists’ clinical services. *Am Soc Health Syst Pharm.* 58 (7) p569-577.

Mugford, M. Banfield, P. and O’Hanlon, M. (1991). Effects of feedback of information on clinical practice: a review. *BMJ.* 303. p398-402.

Muhammad, MM. and Tu, JV. (2001). Did the major clinical trials of statins affect prescribing behaviour? *Can Med Assoc J.* 164 (12) p1695-1696.

Mullen, PD. Green, LW. and Persinger, GS. (1985). Clinical Trials of patient education for chronic conditions: a comparative meta analysis of intervention types. *Preventive Medicine*. 14. p753-781.

National Association of Health Authorities and Trusts. (1994). Priority setting in the NHS: the drugs budget. *House of Commons health committee enquiry, evidence of NAHAT*. January.

National Health Service Executive and National Prescribing Centre. (1996). Primary Care: The future. *Leeds.*

National Prescribing Centre and NHS Executive. (1998). GP Prescribing Support: a resource document and guide for the new NHS. *London: DoH.* September.

Nazareth, I. Freemantle, N. Duggan, C. Mason, J. and Haines, A. (2002). Evaluation of a complex intervention for changing professional behaviour: the Evidence Based Outreach Trial (EBOR). *J of Health Services & Research Policy.* 7 (4) p230-238.

New, JP. Mason, JM. Freemantle, N. Teasdale, S. Wong, L. Bruce, NJ. Burns, JA. and Gibson, JM. (2004). Educational outreach in diabetes to encourage practice nurses to use primary care hypertension and hyperlipidaemia guidelines (EDEN): a randomised controlled trial. *Diabet Med.* 21 (6) p599-603.

Newton–Syms, FAO. Dawson, PH. Cooke, J. Feely, M. Booth, TG. Jerwood, D. and Calvert, RT. (1992). The influence of an academic representative on prescribing by general practitioners. *Br J Clin Pharmacol.* 33. p9-73.

Nguyen, G. Cruikshank, J. Mouillard, A. Dumuis, ML. and Picard, C. et al. (2000). Comparison of achievement of treatment targets as perceived by physicians and as calculated after implementation of clinical guidelines for the management of hypercholesterolaemia in a randomised, clinical trial. *Current Therapeutic Research, Clinical and Experimental.* 61 (9) p597-608.

NHS Centre for Reviews and Dissemination. (2004). Database of Abstracts of Reviews of Effectiveness. Impact of pharmacists providing a prescription review and monitoring service in ambulatory care or community practice. 2.

Nilsson, G. Hjemdahl, P. Hassler, A. Vitols, S. Wallen, NH. and Krakau, I. (2001). Feedback on prescribing rate combined with problem oriented pharmacotherapy education as a model to improve prescribing behaviour among general practitioners. *Eur J Clin Pharmacol.* 56 (1) p843-8.

Northridge, DB. Shandall, A. Rees, A. and Buchalter, B. (1994). Inadequate management of hyperlipidaemia after coronary bypass surgery shown by medical audit. *Br Heart J.* 72. p466-467.

O’Brien, T. Oxman, AD. Davis, DA. Haynes, RB. Freemantle, N. and Harvey, EL. (2002). Educational Outreach visits: effects on professional practice and health care outcomes. *The Cochrane Database of Systematic Reviews.* The Cochrane Library, Vol 1 (1).

O’Donnell, C. MacKenzie, M. Reid, M. Turner, F. Wang, Y. Sridharan, S. and Platt. S. (2009). National Evaluation of Keep Well: Strategies for Reaching the Target Population. *Internal report:* *University of Glasgow.*

Oxman, AD. Davis, AD. Thomson, MA. Davis, DA. and Haynes, RB. (1995). No magic bullets: a systematic review of 102 trials of interventions to improve professional practice. *Can Med Assoc J.* p1423-1431.

Packam, C. Robinson, J. Morris, J. Richards, C. Marks, P. and Gray, D. (1999). Statin prescribing in Nottingham general practices: a cross sectional study. *J Pub Health Med.* 21. p60-64.

Parish, PA. (1973). Drug prescribing: the concern of all. *J Roy Soc Health.* 4. p213-7.

Pearson, SA. Ross Degnan, D. Payson, A. and Soumerai, SB. (2003). Changing medication use in managed care: a critical review of the available evidence. *Am J Man Care.* 9. p715-731.

Pearson, TA. Laurora, I. Chu, H. and Kafonek, S. (2000). The Lipid Treatment Assessment Project (L-TAP). A multicenter study to evaluate the percentages of Dyslipidaemic patients receiving Lipid lowering therapy and achieving low density lipoprotein cholesterol goals. *Arch Int Med.* 160. p459- 467.

Peterson, GM. Bergin, JK. Nelson, BJ. and Stanton, LA. (1996). Improving drug use in rheumatic disorders. *Journal of clinical pharmacy and therapeutics.* 21 (4) p215-220.

Phillips, C. Smith, R. and Pittard, J. (2000). Secondary prevention with statin therapy: a budgetary impact assessment for UK primary care. *J Drug Assess.* 3 (2) p105-17.

Pierce, M. Lundy, S. Palanisamy, A. Winning, S. and King, J. (1989). Prospective randomised controlled trial of methods of call and recall for cervical cytology screening. *BMJ.* 299. p160-2.

Pill, R. Stott, N. Rollnick, S and Rees, M. (1998). A controlled trial of an intervention designed to improve the care given in general practice to Type II diabetic patients: patient outcomes and professional ability to change behaviour. *Fam Prac.* 15 (3) p229-235.

Pilling, M. (1998). The St Helens and Knowsley prescribing initiative model for pharmacist led meetings with GPs. *Pharm J.* 260. p100-2.

Pocock, SJ. (1983). Clinical Trials. A practical approach. *New York: John Wiley.* Section 5.3.

Powell, C. (1997). Consultant pharmacists – what are they all about? *Pharm J.* 258. p65-8.

Primatesta, P. and Poulter, NR. (2000). Lipid concentrations and the use of lipid lowering drugs: evidence from a national cross sectional survey. *BMJ.* 321. p1322-5.

Prochaska, J. and DiClementi, C. (1984). The Trans-Theoretical Approach. *Krieger Publishing, London.*

QOF Achievement Data. (2005). At Strategic Health Authority and England Level.

Available: <http://www.icservices.nhs.uk/qofdocuments/QOF0405_SHAs_ClinicalSummary.xls> Last accessed 12th December 2005.

Raisch, DW. (1990a). A model of methods for influencing prescribing: Part I. A review of prescribing models, persuasion theories and administrative and educational methods. *DICP Ann Pharmacother.* 24. p417-21.

Raisch, DW. (1990b). A model of methods for influencing prescribing: Part II. A review of educational methods, theories of human inference, and delineation of the model. *DICP Ann Pharmacother.* 24 p537-42.

Ramsay, S. Whincup, P. Lawlor, D. Papacosta, O. (2006). Secondary prevention of coronary heart disease in older patients after the national service framework: population based study. *BMJ.* 387. p770-6.

Reid, FDA. (2002). Use of statins in the secondary prevention of coronary heart disease: is treatment equitable? *Heart.* 88. p15-9.

Reilly. PM. Horne, RA. Gilleghan, JD. and Eckersley, APR. (1989). How to produce a practice formulary. *London: Royal College of General Practitioners.* London: RCGP.

Renders, CM. Valk, GD. Griffin, S. Wagner, EH. Eijk, J. and Assendelft, WJJ. (2005). Interventions to improve the management of diabetes mellitus in primary care, outpatient and community settings. *Cochrane Database of Systematic Reviews.* 3.

Rodgers, S. Avery, AJ. Meechan, D. Briant, S. Geraghty, M. Doran, K. and Whynes, DK. (1999). Controlled trial of pharmacist intervention in general practice: the effect on prescribing costs. *Br J Gen Pract.* 49 (446) p717-20.

Roland, M. Holden, J. and Campbell, S. (1998). Quality assessment for general practice: supporting clinical governance in primary care groups. *Manchester: National Primary Care Research and Development Centre*.

Roland, M. (2004). Linking physicians’ pay to the quality of care – a major experiment in the United Kingdom. *N Eng J Med.* 351. p1448-54.

Ruteledge, P. Crookes, D. Mckinstry, B. and Maxwell, SR. (2003). Do doctors rely on pharmaceutical industry funding to attend conferences and do they perceive that this creates a bias in their drug selection? Results from a questionnaire survey. *Pharmacoepidemiology and Drug Safety.* 12 (8) p663-7.

Sacks, FM. Pfeffer, MA. Moyle, LA. and Rouleau, JL. (1996). The effect of pravastatin on coronary events after myocardial infarction in patients with average cholesterol levels. (CARE). *N Engl J Med.* 335. p1001-9.

Sbarbaro, JA. (2001). Can we influence prescribing? *Clinical Infectious diseases.* 33 (3) S240-S244.

Scandinavian Simvastatin Survival Study Group. (4S) (1994). Randomised trial of cholesterol lowering in 4444 patients with coronary heart disease: the Scandinavian Simvastatin Survival Study (4S). *Lancet.* 344. p1383-9.

Schaffner, W. Ray, WA. Federspiel, CF. and Miller, WO. (1983). Improving antibiotic prescribing in office practice. A controlled trial of three educational methods. *JAMA.* 250 (13) p1728-32.

Schectman, JM. Kanwal, NK. Schroth, WS. and Elinsky, EG. (1995). The effect of an education and feedback intervention on group model and network model health maintenance organisation physician prescribing behaviour. Medical Care33 (2) p139-144.

Schmidt, I. Claesson, CB. Westerholm, B. Nilsson, LG. and Svarstad, BL. (1998). The impact of regular multidisciplinary team interventions on psychotropic prescribing in Sweedish Nursing Homes. *J Am Ger Soc.* 46 (1) p77-82.

Schumock, GT. Walton, SM. Park, HY. Nutescu, EA. Blackburn, JC. Finley, JM. and Lewis, RK. (2004). Factors that influence prescribing decisions. *Ann Pharmacother.* 38 (4) p557-62.

Schwartz, RK. Soumerai, SB. and Avorn, J. (1989). Physician motivations for non-scientific drug prescribing. *Soc Sci Med.* 28 (6) p577-82.

Scottish Intercollegiate Guidelines Network Publication 40. Lipids and the Primary Prevention of Coronary Heart Disease.

Available: <http://www.show.scot.nhs.uk/sign/guidelines/fulltext/40/index.html>

Sempos, CT. Cleeman, JI. and Carroll, MD. (1993). Prevalence of high blood cholesterol among U.S. adults. *JAMA.* 269. p3009–14.

Siegel, D. Lopez, J. and Meier, J. (2000). Use of cholesterol lowering medications in the United States from 1991 – 1997. *Am J Med.* 108. p496-499.

Siegel, D. Lopez, J. Meier, J. Goldstein, M. Lee, S. Brazill, B. and Matalka, M. (2003). Academic detailing to improve antihypertensive prescribing patterns. *Am J Hyperten.* 16. p508-511.

Simpson, JM. Klar, N. and Donner, A. (1995). Accounting for cluster randomisation: a review of primary prevention trials, 1990 through 1993. *Am J Public Health.* 85. p1378-83.

Shaw, B. Cheater, F. Baker, R. Gilles, C. Hearnshaw, H. Flottorp, S. and Robertson, N. (2005). Tailored interventions to overcome barriers to change. *Cochrane Database of Systematic Reviews*. 3.

Shepherd, J. Cobbe, SM. Ford, I. and Isles, CG. (1995). Prevention of coronary heart disease with pravastatin in men with hypercholesterolaemia. *N Eng J Med.* 333. p1301-7.

Sloan, KL. (2001). Frequency of serum low density lipoprotein cholesterol measurement and frequency of results ≤ 100mg/dl among patients who had coronary events. *Am J Cardiol.* 88. p1143-6.

Smith, DA. (2001). Comparison of physician managed lipid lowering care in patients with coronary heart disease in two time periods (1994 and 1999). *Am J Cardiol.* 88. p1417-9.

Smith, L. and McClenahan, J. (2000). Evaluation of the Purchaser-led Implementation Programme. In: Evans D, Haines A eds. Implementing evidence based changes in healthcare. *Radcliffe Medical Press.*

Smith, WR. (2003). An explanation of theories that guide evidence based interventions and improve quality. Clinical Governance: *An International Journal.* 8. p247-54.

Solomon, DH. Van Houten, L. Glynn, RJ. Baden, L. Curtis, K. Schrager, H. and Avorn, J. (2001). Academic detailing to improve use of broad spectrum antibiotics at an academic medical centre. *Arch Int Med.* 161 (15) p1897-902.

Somerset, M. Weiss, M. and Fahey, T. (2001). Dramaturgical study of meetings between general practitioners and representatives of pharmaceutical companies. BMJ. 323. p1481-1484.

Soumerai, SB. and Avorn, J. (1986). Economic and policy analysis of university based detailing. *Medical Care.* 24. p313-331.

Soumerai, SB. and Avorn, J. (1987). Predictors of physician prescribing change in an educational experiment to improve medication use. *Medical Care.* 25 (3) p201-221.

Soumerai, SB. McLaughlin, TJ. and Avorn, J. (1989). Improving drug prescribing in primary care: a critical analysis of the experimental literature. *The Millbank Quarterly*. 67. p268-317.

Soumerai, SB. and Avorn, J. (1990). Principles of educational outreach (“academic detailing”) to improve clinical decision making. *JAMA.* 263. p549-556.

Soumerai, SB. McLaughlin, TJ. and Avorn, J. (1990). Quality assurance for drug prescribing. *Quality Assurance in Health Care.* 2. p37-58.

Soumerai, SB. (1998). Principles and uses of academic detailing to improve the management of psychiatric disorders. *Int J Psych Med.* 28 (1) p81-86.

Squires, S. (1997). PACT based prescribing project. In National Prescribing Centre. GP prescribing – developments in professional support; *Liverpool: NPC.* p36.

Steele, MA. Bess, DT. Franse, VL. and Graber, SE. (1989). Cost effectiveness of two interventions for reducing outpatient prescribing costs. *DICP.* 23. p497-500.

Sudlow, M. Rodgers, H. Kenny, R. and Thomson, R. (1997). Population based study of use of anticoagulants among patients with atrial fibrillation in the community. *BMJ.* 314. p1529-1530.

Sueta, C. Chowdhury, M. and Boccuzzi, SJ. et al. (1999). Analysis of the degree of undertreatment of hyperlipidaemia and congestive heart failure secondary to coronary heart disease. *Am J Cardiol.* 83. p1303-1307.

Sweeney, KG. MacAuley, D. and Gray, DP. (1998). Personal significance: the third dimension. *Lancet.* 351. p134-136.

Tamblyn, RM, Jacques, A. Laprise, R. Huang, A. and Perreault, R. (1997). The office of the future project: the integration of new technology into office practice. Academic detailing through the super highway. Quebec Research Group on Medication Use in the elderly. *Clin Performance and Quality in Healthcare.* 5 (2) p104-8.

Tant, D. (1999). Having practice pharmacists is not the only way of reducing prescribing costs. *BMJ.* 318. p872.

Temple, J. (2002). *BMJ*. 324. p674 letter.

Thomas, L. Cullum, N. McColl, E. Rousseau, N. Soutter, J. and Steen, N. (2000). Guidelines in professions allied to medicine. *The Cochrane Database of Systematic Reviews*. 2.

Thomson, MA. Oxman, AD. Haynes, RB. Davis, DA. Freemantle, N. and Harvey, EL. (2005). Local opinion leaders. *Cochrane Database of Systematic Reviews.* 3.

Tully, MP. and Seston, EM. (2000). Impact of pharmacists providing a prescription review and monitoring service in ambulatory care or community practice. *Ann Pharmacother.* 34 (11) p1320-1331.

Van Eijk, M. Avorn, J. Porsius, AJ. and De Boer, A. (2001). Reducing prescribing of highly anticholinergic antidepressants for elderly people: randomized trial of group versus individual academic detailing. *BMJ.* 322. p654-663.

Van Eijk, M. Paes, AH. Porsius, A. Avorn, J. and De Boer, A. (2004). Pre-randomisation decisions and group stratification in a randomised controlled trial to improve prescribing. *Pharmacy and world science.* 26 (4) p227-31.

VanStaa, TP. and Abenhaim, L. (1994). The quality of information recorded on a UK database of Primary Care records: a study of hospitalisations due to hypoglycaemia and other conditions. *Pharmacoepidimiol Drug Safety.* 3. p15-21.

Veldhuis, M. and Wigersma, L. and Okkes, I. (1998). Deliberate departures from good general practice: a study of motives among Dutch general practitioners. *Br J Gen Pract.* 48. p1833-1836.

Veninga, CC. Lagerlov, P. Wahlstrom, R. Muskova, M. Denig, P. and Berhov, J. (1999). Evaluating an educational intervention to improve the treatment of asthma in four European Countries. Drug Education Project Group. *Am J Resp and Crit Care Med.* 160 (4) p1254-62.

Veninga, CC. Denig, P. Zwaagstra, R. and Haaijer-Ruskamp, FM. (2000). Improving drug treatment in general practice. *J Clin Epidemiology.* 53 (7) p762-72.

Virji, A. and Britten, N. (1991). A study of the relationship between patients’ attitudes and doctors’ prescribing. *Fam Pract*. 8. p314-319.

Wakefield, J. Herbert, CP. Maclure, M. Dormuth, C. Wright, JM. Legare, J. Brett-Maclean, P. and Premi, J. (2003). Commitment to change statements can predict actual change in practice. *Journal of Continuing Education in the Health Professions.* 23 (2) p81-93.

Ward, P. Noyce, P. and St Leger, A. (2007). How equitable are GP practice prescribing rates for statins?: an ecological study in four primary care trusts in North West England. *Int J Equ Health.* 6. p2.

Watkins, C. Harvey, I. Carthy, P. Moore, L. Robinson, E. and Brawn R. (2003). The attitudes and behaviours of General Practitioners and their prescribing costs: a national cross sectional survey. *Quality and Safety in Health Care.* 12. p29-34.

Watson, MC. Bond, C. Grimshaw, JM. Mollison, J. Ludbrook, A. and Walker, AE. (2002). Educational strategies to promote evidence based community pharmacy practice: a cluster randomised controlled trial. *Fam Pract.* 19 (5) p529-536.

Watson, M. Gunnell, D. Peters, T. Brookes, S. and Sharp, D. (2001). Guidelines and educational outreach visits from community pharmacists to improve prescribing in general practice: a randomised controlled trial. *J Health Serv Res Policy.* 6 (4) p207-213.

Webb, S. Lloyd, M. (1994). Prescribing and referral in general practice: a study of patients’ expectations and doctors’ actions. *Br J Gen Pract.* 44. p165-169.

Wells, WDE. (1997). Pharmacists are key members of primary health care teams. *BMJ.* 314. p1486.

Wells, WDE. (1998). Having a practice pharmacist can reduce prescribing costs. *BMJ.* 317. p473.

Welschen, I. Kuyvenhoven, MM. Hoes, AW. and Verheij, TJ. (2004). Effectiveness of a multiple intervention to reduce antibiotic prescribing for respiratory tract symptoms in Primary Care: randomised controlled trial. *BMJ*. doi:10.1136/bmj.38182.591238.EB.

Whincup, P. (2002). Low prevalence of lipid lowering drug use in older men with established coronary heart disease. *Heart.* 88. p25-9.

Whitelaw, FG. Taylor, RJ. Nevin, SL. Taylor, MW. Milne, RM. and Watt, AH. (1996). Completeness and accuracy of morbidity and repeat prescribing records held on practice computers in Scotland. *Br J Gen Pract.* 46. p181- 186.

Whiting-0’Keefe, QE. Henke, C. and Simborg, DW. (1984). Choosing the correct unit of analysis in medical care experiments. *Med Care.* 22. p1101-1114.

Anon. Who should provide a clinical pharmacy service to primary care? (1996). *Pharm J*. 257. p493.

Witt, K. Knusden, E. Ditlevsen, S. and Hollnagel, H. (2004). Academic detailing has no effect on prescribing of asthma medication in Danish general practice: a 3 year randomised controlled trial with 12-monthly follow ups. *Family Practice*. 21 (3) p248-53.

Wyatt, TD. Reilly, PM. Morrow, NC. and Passmore, CM. (1992). Short lived effects of a formulary on anti-infective prescribing – the need for continuing peer review. *Fam Pract.* 9. p461-465.

Yeo, GT. De Burgh, SP. Letton, T. Shaw, J. Donnelly, N. Swinburn, ME. Phillips, S. Bridges Webb, C. and Mant, A. (1994). Educational visiting and hypnosedative prescribing in general practice. *Fam Pract.* 11 (1) p57-61.

Zwar, NA. Wolk, J. Gordon, JJ. and Sanson-Fisher, RW. (2000). Benzodiazepine prescribing by GP registrars. A trial of educational outreach. *Australian Family Physician.* 29 (11) p1104-7.
